# Internal cranial anatomy of Early Triassic species of †*Saurichthys* (Actinopterygii: †Saurichthyiformes): implications for the phylogenetic placement of †saurichthyiforms

**DOI:** 10.1186/s12862-018-1264-4

**Published:** 2018-11-01

**Authors:** Thodoris Argyriou, Sam Giles, Matt Friedman, Carlo Romano, Ilja Kogan, Marcelo R. Sánchez-Villagra

**Affiliations:** 1 Palaeontological Institute and Museum, University of Zurich, Karl Schmid-Strasse 4, 8006 Zurich, Switzerland; 2 Department of Earth Sciences, University of Oxford, South Parks Road, Oxford, OX1 3AN UK; 3 Museum of Paleontogy and Department of Earth and Environmental Sciences, University of Michigan, 1109 Geddes Ave, Ann Arbor, MI 48109 USA; 4 Department of Palaeontology, TU Bergakademie Freiberg, Geological Institute, Bernhard-von-Cotta-Str. 2, 09599 Freiberg, Germany; 5 Kazan Federal University, 18 Kremlyovskaya, Kazan, 420008 Russia

**Keywords:** Actinopterygii, †*Saurichthys*, Chondrostei, *Acipenser*, Triassic, Microtomography (μCT), Phylogeny, Cranial fossae, East Greenland, Nepal

## Abstract

**Background:**

**†**Saurichthyiformes were a successful group of latest Permian–Middle Jurassic predatory actinopterygian fishes and constituted important, widely-distributed components of Triassic marine and freshwater faunas. Their systematic affinities have long been debated, with **†**saurichthyiforms often being aligned with chondrosteans, a group today comprising sturgeons and paddlefishes. However, their character-rich endocranial anatomy has not been investigated in detail since the first half of the 20th century. Since then, major advances have occurred in terms of our understanding of early actinopterygian anatomy, as well as techniques for extracting morphological data from fossils.

**Results:**

We used μCT to study the internal cranial anatomy of two of the stratigraphically oldest representatives of †*Saurichthys*, from the Early Triassic of East Greenland and Nepal. Our work revealed numerous previously unknown characters (e.g., cryptic oticooccipital fissure; intramural diverticula of braincase; nasobasal canals; lateral cranial canal; fused dermohyal), and permitted the reevalution of features relating to the structure of cranial fossae, basicranial circulation and opercular anatomy of the genus. Critically, we reinterpret the former **†**saurichthyiform opercle as an expanded subopercle. For comparison, we also produced the first digital models of a braincase and endocast of a sturgeon (*A. brevirostrum*)*.* New information from these taxa was included in a broad phylogenetic analysis of Actinopterygii. †Saurichthyiforms are resolved as close relatives of †*Birgeria*, forming a clade that constitutes the immediate sister group of crown actinopterygians. However, these and other divergences near the actinopterygian crown node are weakly supported.

**Conclusions:**

Our phylogeny disagrees with the historically prevalent hypothesis favoring the chondrostean affinities of †saurichthyiforms. Previously-proposed synapomorphies uniting the two clades, such as the closure of the oticooccipital fissure, the posterior extension of the parasphenoid, and the absence of an opercular process, are all widespread amongst actinopterygians. Others, like those relating to basicranial circulation, are found to be based on erroneous interpretations. Our work renders the †saurichthyiform character complex adequately understood, and permits detailed comparisons with other stem and crown actinopterygians. Our phylogenetic scheme highlights outstanding questions concerning the affinity of many early actinopterygians, such as the Paleozoic–early Mesozoic deep-bodied forms, which are largely caused by lack of endoskeletal data.

**Electronic supplementary material:**

The online version of this article (10.1186/s12862-018-1264-4) contains supplementary material, which is available to authorized users.

## Background

Actinopterygii (ray-finned fishes), with more than 32,000 living species [1], encompass over half of extant vertebrate species and possess an evolutionary history of at least 415 myr [2, 3]. This extant diversity is unevenly distributed among three major clades: Cladistia (bichirs and the reedfish), Chondrostei (sturgeons and paddlefishes), and Neopterygii, the latter containing the depauperate Holostei (gars and the bowfin) and the very speciose Teleostei [4, 5]. The monophyly of these three modern clades is well-supported, and identification of fossil members within them is fairly uncontroversial [6–9] (but see [10]). However, with the exception of some derived fossils that branch close to the crown radiation, for example †*Chondrosteus* in the case of Chondrostei [11], the content of more distant portions of the stems of the three major actinopterygian lineages is highly equivocal. In spite of considerable differences in details, molecular and paleontological timescales place the divergence of these three lineages in the mid-late Paleozoic [4, 5, 12]. Abundant fossil actinopterygians of Paleozoic and early Mesozoic age are known [13–15], but their systematic placement relative to neopterygians, chondrosteans, and cladistians is highly unstable and variable between phylogenetic analyses [12, 16–20]. Although some of this ambiguity doubtlessly reflects genuine character conflict, the limited documentation of anatomy in many fossils of this age presents the chief obstacle.

Set against this backdrop, the latest Permian [21] to Middle Jurassic [22] †saurichthyids represent a case of contested evolutionary history. This group of predatory actinopterygians is characterized by an elongate body, a prominent rostrum, posteriorly situated median fins and an unusual abbreviated-diphycercal tail-fin [23–30]. †*Saurichthys*, the iconic representative of the family, encompasses at least two or more potential subgenera, including †*Sinosaurichthys* [30] and likely †*Saurorhynchus* [27]. The type species, †*Saurichthys apicalis* [31], is known from a fragmentary rostrum. †*Yelangichthys* (†Yelangichthyidae), a durophagous form from the Middle Triassic of China, has been identified as the sister lineage of †saurichthyids [32], and with them forms the †Saurichthyiformes.

†*Saurichthys* is known from thousands of specimens belonging to over 40 nominal species, associated with marine, freshwater and brackish settings and occurring on all continents except Antarctica and South America [14, 25]. Abundant and well-preserved fossils permit investigation of soft-tissue features, with studies revealing reproductive mode and details of ontogeny [33–35], mode of axial elongation [26, 36], swimming mode and efficiency [37], as well as gastrointestinal anatomy [38]. Although the wealth of potential paleobiological information about †*Saurichthys* is unrivalled among early fossil actinopterygians, some basic anatomical aspects of this genus are known in limited detail relative to other taxa.

Key to understanding the systematic placement of †saurichthyids is the character rich internal anatomy of the cranium (which can constitute up to 80% of published character matrices), comprising the braincase and associated dermal bones, suspensorium, and hyoid and branchial arches. Stensiö [29], based on direct observations and serial grinding of mechanically prepared, three-dimensionally preserved fossils from the Early Triassic of Spitsbergen, Svalbard, provided a lengthy, but somewhat idealized, account of the character-rich internal cranial anatomy of †*Saurichthys*. Few additions on the internal cranial anatomy of †*Saurichthys* have been made by subsequent authors [39, 40]. Critically, Stensiö’s [29] observations on †*Saurichthys*, and his conclusion of a close relationship with acipenseriforms, set the stage for most later phylogenetic intepretations of non-neopterygian actinopterygians and the widespread association of †*Saurichthys* with Chondrostei [16–19, 24, 27, 32].

Numerous anatomical similarities have been treated as features supporting a chondrostean placement for †saurichthyids, such as: i) ethmoidal elongation; ii) presence of large craniospinal processes; iii) absence of parabasal canals and a circulus cephalicus; iv) presence of a spiracular canal; v) absence of a lateral cranial canal; vi) absence of a basipterygoid process; vii) posteriorly expanded parasphenoid reaching the basioccipital; viii) absence of gulars; ix) reduced squamation. However, many of these features are either more general in their distribution, or are demonstrably homoplastic within non-neopterygian actinopterygians.

Phylogenetic schemes that resolve †*Saurichthys* outside the chondrostean clade, but with uncertain placement within non-neopterygian actinopterygians, have also been proposed [41, 42]. †*Saurichthys* has additionally been interpreted as a stem neopterygian, on the basis of the reduction of the branchiostegal series and the presence of elongate epaxial rays [18]. However, past solutions were often a product of limited taxon sampling [16–18, 24], and/or were based on matrices aimed at resolving relationships within the †saurichthyid clade and lacking broader taxonomic context [24, 26, 27, 32]. In many cases, terminal taxa taken into account were coded as composites [16–19, 41]. These interpretations were also influenced by critical errors in the coding of characters (see discussion). A more recent analysis, drawing characters from a variety of sources and coding a single, non-composite taxon recovered †*Saurichthys* as the immediate sister taxon to the actinopterygian crown [12], but this study is still hampered by a limited taxonomic sampling of †saurichthyids and lack of data related to their cranial endoskeleton.

Considering the important phylogenetic position †saurichthyids seem to occupy relative to the actinopterygian crown, as well as the unparalleled amount of paleobiological information available for these animals a critical reinvestigation of their internal cranial anatomy and interrelationships is warranted. In this work, we employ μCT in order to study the structure of the skull in two Early Triassic specimens of †*Saurichthys*, which are amongst the stratigraphically oldest representatives of the clade. The main goals of this work are: 1) to provide an up-to-date account of the internal cranial anatomy of †*Saurichthys*; 2) to test the classical models of internal cranial anatomy, which were produced with the use of destructive techniques [29]; and 3) to reappraise the phylogenetic affinities of †saurichthyids among actinopterygians generally, and to chondrosteans specifically, based on a combination of new information from μCT investigation and an expanded character-by-taxon matrix. In addition, to improve the available comparative material, we provide the first digital models of the braincase and endocast of *Acipenser*. Finally, given the lack of nomenclatural consistency in the literature, and aided by our observations on †*Saurichthys* and *Acipenser*, we provide a review and discussion on the evolution and function of several cranial fossae in the actinopterygian braincase.

## Methods

Following [43], fossil taxa are preceded by the dagger symbol (†) throughout the text.

### Comparative materials

#### †Saurichthyiformes

PIMUZ A/I 4648, unnamed †saurichthyid from the Prosanto Formation (Ladinian, late Middle Triassic Canton Graubünden, Switzerland) exhibiting hyoid, lower jaw and opercular anatomy.

#### †Pteronisculus

NHMD_73588_A, †*Pteronisculus gunnari,* physical holotype and scan of specimen including a complete skull with lower jaw, opercular series and pectoral girdle attached (Griesbachian, early Induan, Early Triassic; East Greenland).

#### Acipenseriformes

FMNH 113538, *Acipenser brevirostrum*, scan of braincase and parasphenoid; UMMP teaching collection, *Acipenser* sp., disarticulated skeleton; UMMP teaching collection, *Acipenser* sp., skull with suspensorium, lower jaw, hyoid and branchial arches, and pectoral girdle attached; UMMP teaching collection, *Polyodon spathula*, two complete and partially disarticulated dry skeletons; UMMZ 64250, *Acipenser brevirostrum*, scan of stained head.

#### Holostei

PIMUZ A/I 4171a, skull of *Atractosteus spatula*; UMMP teaching collection, *Amia calva*, skull with suspensorium, lower jaw, hyoid and branchial arches attached.

### Anatomical nomenclature

Our discussion of the neurocranium of †*Saurichthys* focuses on four broad regions (occipital, otic, orbitosphenoid and ethmoid), following Gardiner [44]. Anatomical terminology for general cranial anatomy follows Gardiner [44] and Kogan & Romano [25] for the dermal skull specifically. To aid the reader, we have included abbreviations of anatomical structures depicted in the figures throughout the text. The abbreviations are also explained in the figure legends.

### Tomographic and digital rendering methods

The scan of the Greenland †*Saurichthys* (NHMD_157546_A) was performed using a using a Nikon XT H 225 ST scanner at the University of Bristol Palaeobiology Research Group, Bristol, U.K. The specimen was scanned in three stacks, which were subsequently stitched together. The same parameters were used for each scan, as follows: 220 kV, 110 uA, no filtering. The scan of †*Saurichthys nepalensis* (MNHN F 1980–5) was performed at the Muséum National d’Histoire Naturelle, Paris, France, with a AST-RX scanner. The scan parameters were as follows: 120 kV, 480uA, filtered with 0.5 mm of copper. The scan of *Acipenser brevirostrum* (FMNH 113538) was performed in the CTEES facilty of the University of Michigan using a Nikon XT H 225 ST scanner. The scan parameters were as follows: 75 kV, 290 uA, no filtering. The resulting volumes were segmented using Mimics Research v19.0 (biomedical.materialise.com/mimics; Materialise, Leuven, Belgium). The resulting 3D objects were exported as PLY files and processed in Blender (blender.org) for imaging purposes.

### Phylogenetic dataset assembly and analyses

For our phylogenetic analyses, we modified and expanded the morphological matrix developed by Giles et al. [12] using Mesquite Version 3.2 [45]. We removed a total of three characters (pertaining to the presence or absence of lepidotrichia; hypohyal; pelvic fins), due to their uninformative status. We now treat C.256 (presence and arrangement of scutes anterior to the dorsal fin) as unordered. Twelve new binary and one multistate morphological characters (C.20; C.24; C.44; C.112; C.154; C.170; C.181; C.189; C.204; C.205; C.212-multistate; C.228; C. 268), a third state for C.159 and a fourth state for C.177 were added, resulting to a total of 275 equally weighted characters (see Additional files 1, 2). †*Brachydegma caelatum* was also removed from the matrix, since a major reinterpretation of its anatomy is pending following μCT investigation (Argyriou et al. in prep.). We added five new taxa, giving a total of 97 taxa in our dataset. In order to test the monophyly of saurichthyiforms we included: 1) the Early Triassic †*Saurichthys* from Greenland (NHMD_157546_A); 2) the Early Triassic †*Saurichthys ornatus* (coded after [29]); 3) the Middle Triassic saurichthyiform †*Yelangichthys macrocephalus* (coded after [32]); 4) *Polyodon spathula* was included as an additional member of the chondrostean crown (coded after our observations on UMMP dry skeletons); 5) †*Birgeria stensioei* from the Middle Triassic of Monte San Giorgio (coded after [46, 47]). Finally, we extensively rescored *Acipenser brevirostrum*, †*Birgeria groenlandica* and †*Saurichthys madagascariensis*, and changed the scoring in some additional taxa (rescoring details in Additional files 1, 2).

The maximum parsimony analyses were performed with ‘New Technology Searchʼ algorithms implemented in TNT [48]. The ‘placodermʼ †*Dicksonosteus arcticus* was set as outgroup, but we placed a constraint on the monophyly of osteichthyans using an artificial tree that exhibited the following outgroup relationship: (†*Dicksonosteus* (†*Entelognathus* ((†*Acanthodes* (†*Ozarcus* †*Cladodoides*)) Osteichthyes). Following [49], we used a combination of ‘Ratchet’ and ‘Sectorial Search’ algorithms. Initial trees were produced with a combination of ‘Sectiorial Searchʼ, ‘Ratchetʼ, ‘Drift’ and ‘Tree Fusingʼ (1000 trees by RAS with 100 iterations of each mentioned algorithm), while the number of suboptimal trees to be kept was set to 10 and the relative fit difference was set to 0.1. Initial trees were subjected to 2 × 3 consecutive rounds of analyses. The first round comprised 1000 iterations of ‘Sectiorial Searchʼ, complemented by one run of 1000 iterations of ‘Ratchetʼ and another run of ‘Sectiorial Searchʼ. The second round comprised 1000 iterations of ‘Ratchetʼ, followed by 1000 iterations of ‘Sectiorial Searchʼ, and 1000 iterations of ‘Ratchetʼ. Each run was complemented by 1000 iterations of ‘Tree Fusingʼ. Trees resulting from the two rounds of analyses were combined, and all suboptimal trees were discarded, before the calculation of the strict consensus. From all available trees we visualized the distribution of synapomorphies and we calculated an agreement subtree using the relevant function in TNT. Using all trees produced during the successive rounds of analysis, including suboptimals, we calculated Bremer values for clades. The matrix was re-analyzed with ‘Traditional Searchʼ (1000 iterations) for estimating bootstrap supports. The agreement subtree functions implemented in TNT aided the identification of wildcard taxa [48]. The matrix and trees can be found in Additional file 2.

## Results

### Systematic paleontology

ACTINOPTERYGII Cope, 1887 [50] (sensu [51]).

†SAURICHTHYIFORMES Aldinger, 1937 [52] (sensu [32]).

†SAURICHTHYIDAE Owen, 1860 [53] (sensu [29]).

†*SAURICHTHYS* Agassiz, 1834 [31].

†*Saurichthys* sp.

2008 – †*Saurichthys* cf. *ornatus* Mutter et al. [54]

#### Material

NHMD_157546_A, †*Saurichthys* sp., almost complete skull and lower jaw.

#### Fossil age and locality information

The Early Triassic (Induan: Griesbachian–early Dienerian; see also [55]) Wordie Creek Formation of East Greenland contains six well-demarcated horizons (‘Fish Zones I–V’, ‘Stegocephalian Zone’ [56]) that yielded a plethora of vertebrate fossils, including a sizable fossil fish sample, dominated by actinopterygians [56–61]. The bulk of this material is deposited in the collections of the Natural History Museum of Denmark, and a substantial portion of this collection remains unprepared. †*Saurichthys* remains are comparatively rare in East Greenland (< 30 out of over 2,200 identifiable fish fossils collected), and were only recovered from zones II and V, and potentially zone III [54, 56, 57, 60, 62]. The material from horizon II is laterally compressed and was referred to †*Saurichthys* aff. *S. dayi* on the basis of postcranial anatomy, although it likely corresponds to a new species [62]. ‘Fish Zone V’ is the youngest of the ‘Fish Zones’ on East Greenland and is associated with the former ‘*Proptychites* beds’ [56], which likely correspond to the †*Bukkenites rosenkrantzi* zone of late Griesbachian–early Dienerian (~ 250.4 Ma) age [55]. The latter zone has produced at least two three-dimensionally preserved crania, which were identified as †*Saurichthys* cf. *S. ornatus*, on the basis of external anatomical similarities with younger (Smithian Olenekian, late Early Triassic) material from paleogeographically close localities in Spitsbergen [54]. The present work focuses on the better-preserved NHMD_157546_A from the River 7 locality on Kap Stosch, Hold-with-Hope-Peninsula, which was collected during the 1930s. For additional information on local stratigraphy and locality information the reader is referred to [55, 56].

### Anatomical description

#### General features of the neurocranium

**Fig. 1:**
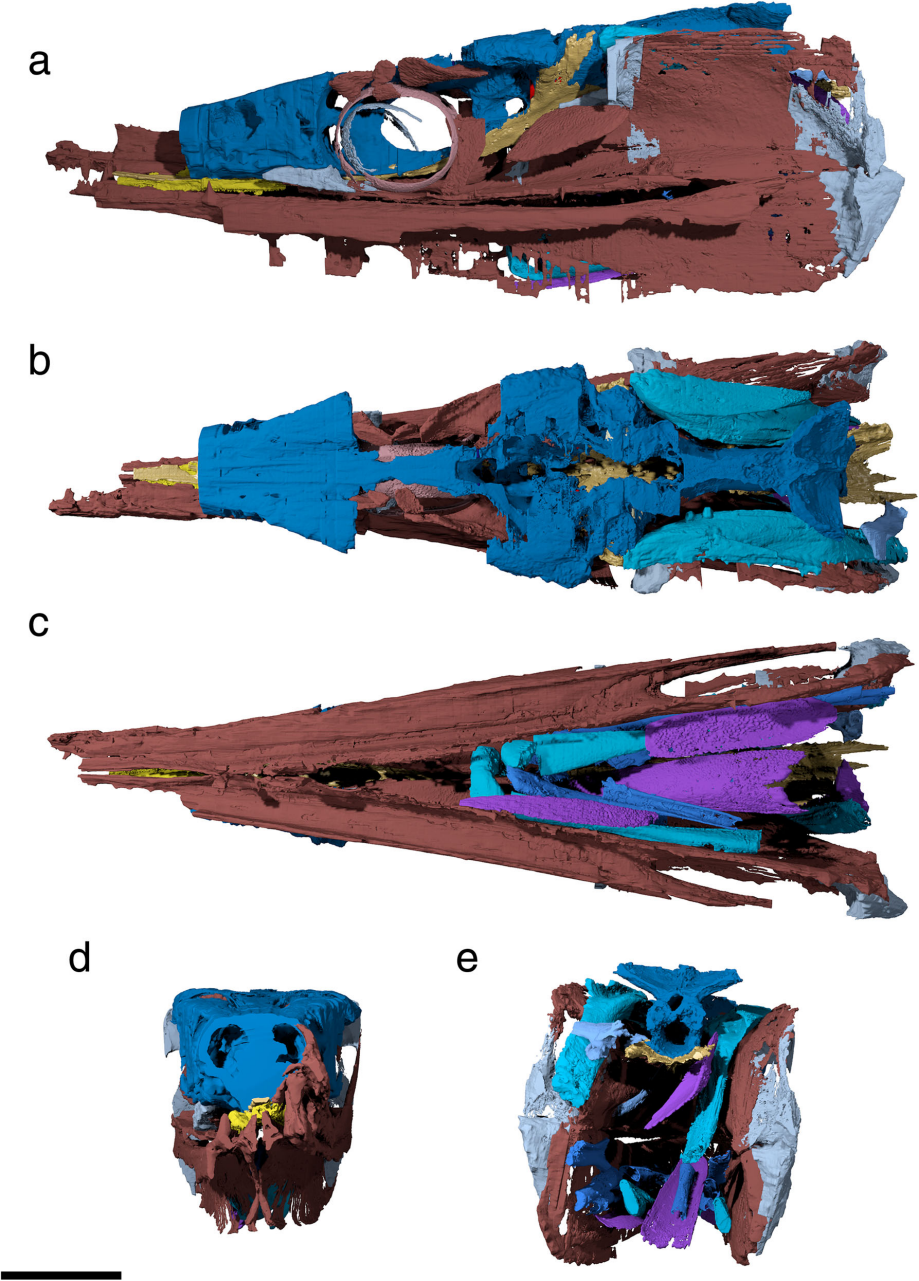
Tomographic renderings of endoskeletal anatomy of †*Saurichthys* sp. (NHMD_157546_A); **a** right lateral (mirrored) view; **b** dorsal view; **c** ventral view; **d** anterior view; **e** posterior view. Blue shades indicate elements of likely endochondral origin (except dermohyal). Earthy–purple shades indicate elements of likely dermal origin. Scale bar equals 1 cm

**Fig. 2:**
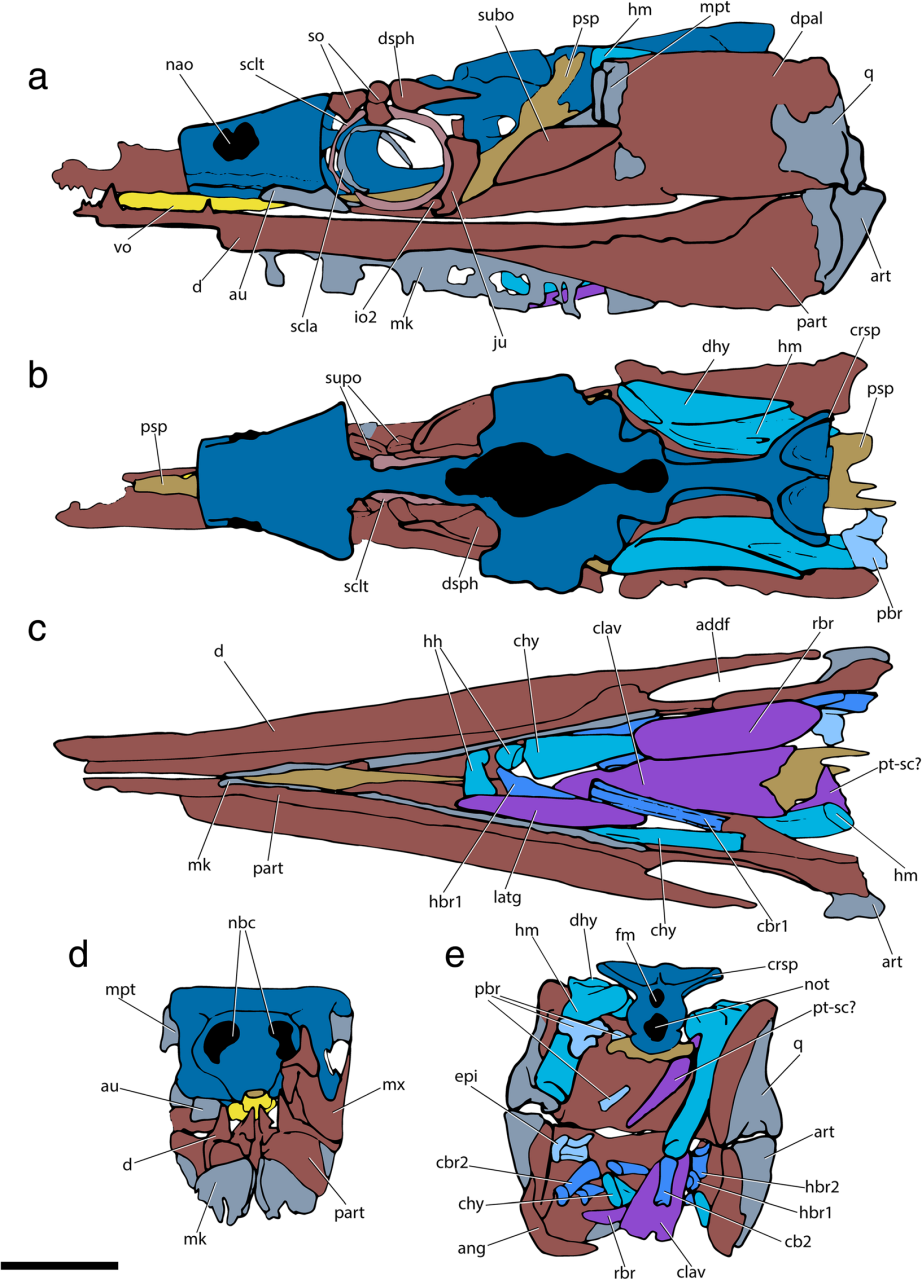
Interpretative drawing of endoskeletal anatomy of †*Saurichthys* sp. (NHMD_157546_A); **a** right lateral (mirrored) view; **b** dorsal view; **c** ventral view; **d** anterior view; **e** posterior view. Blue shades indicate elements of likely endochondral origin (except dermohyal). Earthy–purple shades indicate elements of likely dermal origin. Abbreviations: **addf,** mandibular adductor fossa; **ang,** angular; **art,** articular; **au,** autopalatine; **cbr1,** ceratobranchial 1; **cbr2,** ceratobranchial 2; **chy,** ceratohyal; **clav,** clavicle; **crsp,** craniospinal process; **d,** dentary; **dhy,** dermohyal; **dpal,** dermal palate; **dsph,** dermoshenotic; **epi,** epibranchial; **fm,** foramen magnum; **hh,** hypohyal; **hbr1,** hypobranchial 1; **hbr2,** hypobranchial 2; **hm,** hyomandibula; **io,** infraorbital; **ju,** jugal; **latg,** lateral gular; **mpt,** metapterygoid; **mk,** Meckel’s cartilage; **mx,** maxilla; **nao,** narial opening; **nbc,** nasobasal canal; **not,** notochordal canal; **part,** prearticular; **pbr,** pharyngobranchial; **psp,** parasphenoid; **pt-sc?,** putative posttemporal-supracleithrum; **q,** quadrate; **rbr,** branchiostegal ray; **scla,** sclera; **sclt,** sclerotic ring; **so,** suborbital; **supo,** supraorbital; **vo,** vomer. Scale bar equals 1 cm

**Fig. 3:**
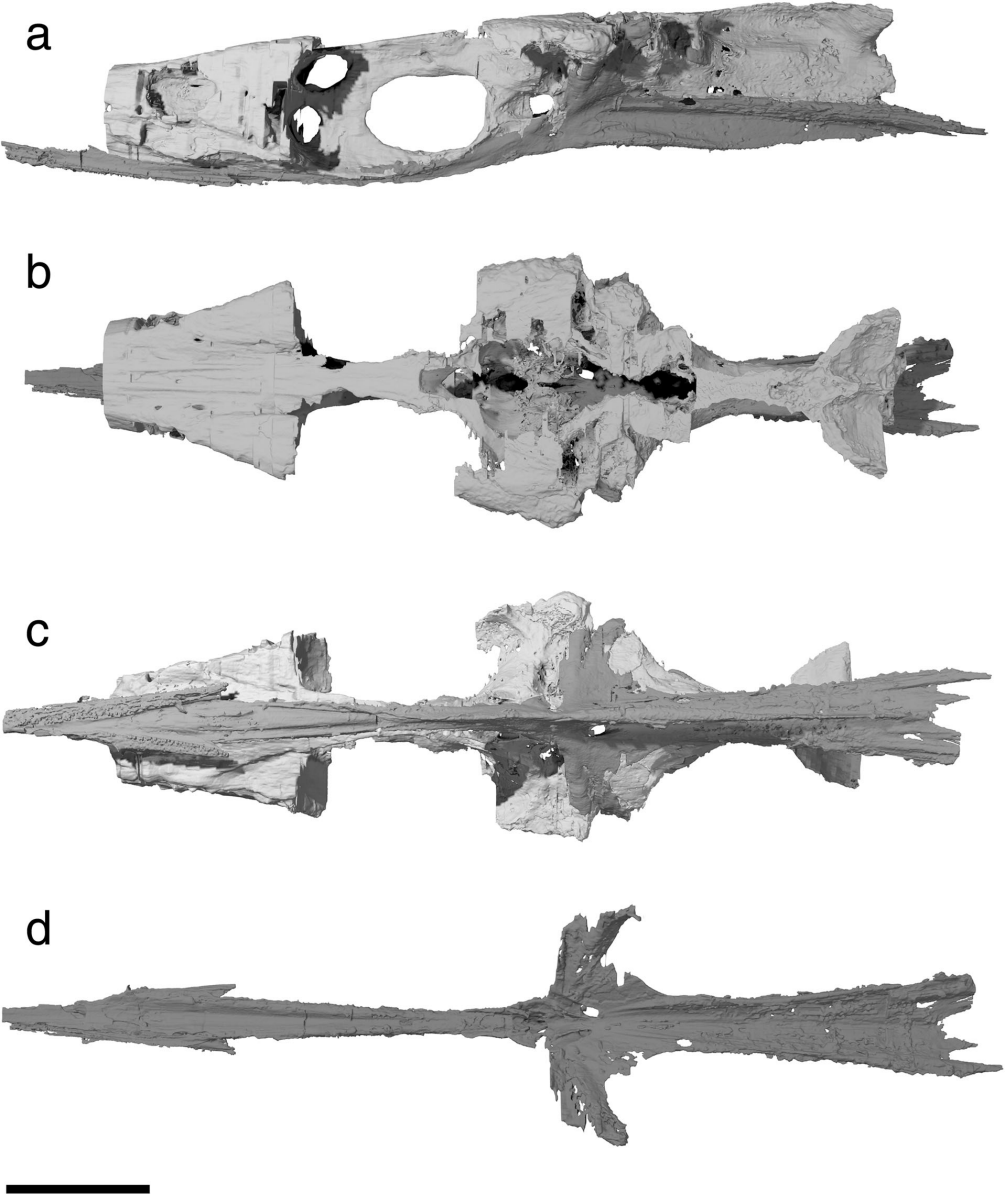
Tomographic renderings of braincase and parasphenoid of †*Saurichthys* sp. (NHMD_157546_A); **a** left lateral view; **b** dorsal view; **c** ventral view; **d** dorsal view of parasphenoid; dark gray shade indicates elements of dermal origin. Scale bar equals 1 cm

**Fig. 4:**
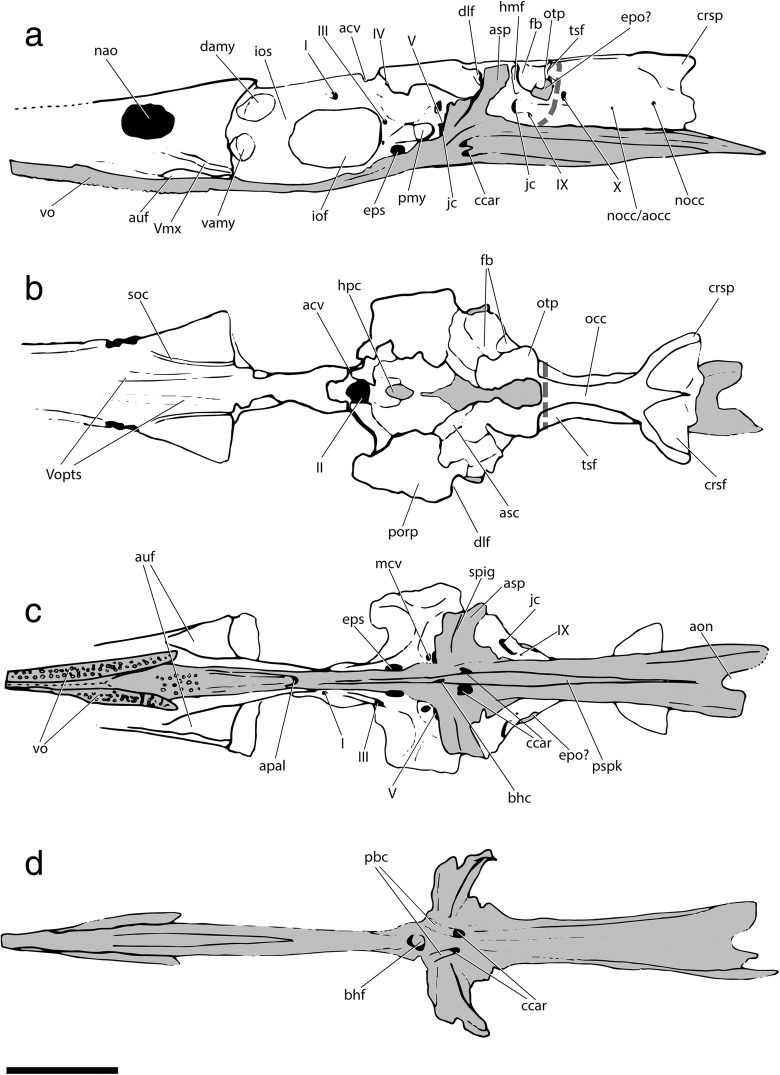
Interpretative drawing of braincase and parasphenoid anatomy of †*Saurichthys* sp. (NHMD_157546_A); **a** left lateral view; **b** dorsal view; **c** ventral view; **d** dorsal view of parasphenoid; gray shade indicates elements of dermal origin. Dashed gray line indicates cryptic oticooccipital fissure. Abbreviations: **I,** olfactory nerve; **II,** optic nerve; **III,** oculomotor nerve; **IV,** trochlear nerve; **V,** trigeminal nerve; **Vmx,** maxillary ramus of trigeminal nerve; **Vopts,** superficial ophthalmic ramus of trigeminal nerve; **IX,** glossopharyngeal nerve; **X,** vagus nerve; **acv,** anterior cerebral vein; **aon,** aortic notch; **apal,** palatine artery; **aps,** pseudobranchial artery; **asc,** anterior semicircular canal; **asp,** ascending process of parasphenoid; **auf,** autopalatine fossa; **bhf,** buccohypophyseal opening; **ccar,** common carotid artery; **crsf,** craniospinal fossa; **crsp,** craniospinal process; **damy,** dorsal anterior myodome; **dlf,** likely origin of dilatator and/or hyomandibular protractor muscles; **epo?,** epiotic-like ossification; **fb,** fossa bridgei; **hmf,** hyomandibular facet; **hpc,** hypophyseal chamber; **iof,** interorbital fenestra; **ios,** interorbital septum; **jc,** jugular canal; **mcv,** mid-cerebral vein; **nao,** narial opening; **nocc,** spinooccipital nerve; **nocc/aocc,** spinooccipital nerve or occipital artery; **occ,** occipital crest; **otp,** otic process; **pmy,** posterior myodome; **porp,** postorbital process; **pspk,** parasphenoid keel; **soc,** trace of supraorbital canal; **spig,** spiracular groove; **tsf,** tectosynotic fossa; **vamy,** ventral anterior myodome; **vo,** vomer. Scale bar equals 1 cm

The specialized neurocranial morphology of †*Saurichthys* is dominated by elongate occipital and ethmoidal (rostral) regions, as well as large orbital spaces (Figs. 1, 2, 3 and 4). In dorsal view the neurocranium is bullet-shaped, attaining its maximum width at the level of the postorbital process. In lateral view, the orbitotemporal and ethmoidal regions are much longer than the occipital and otic regions.

#### Occipital region

**Fig. 5:**
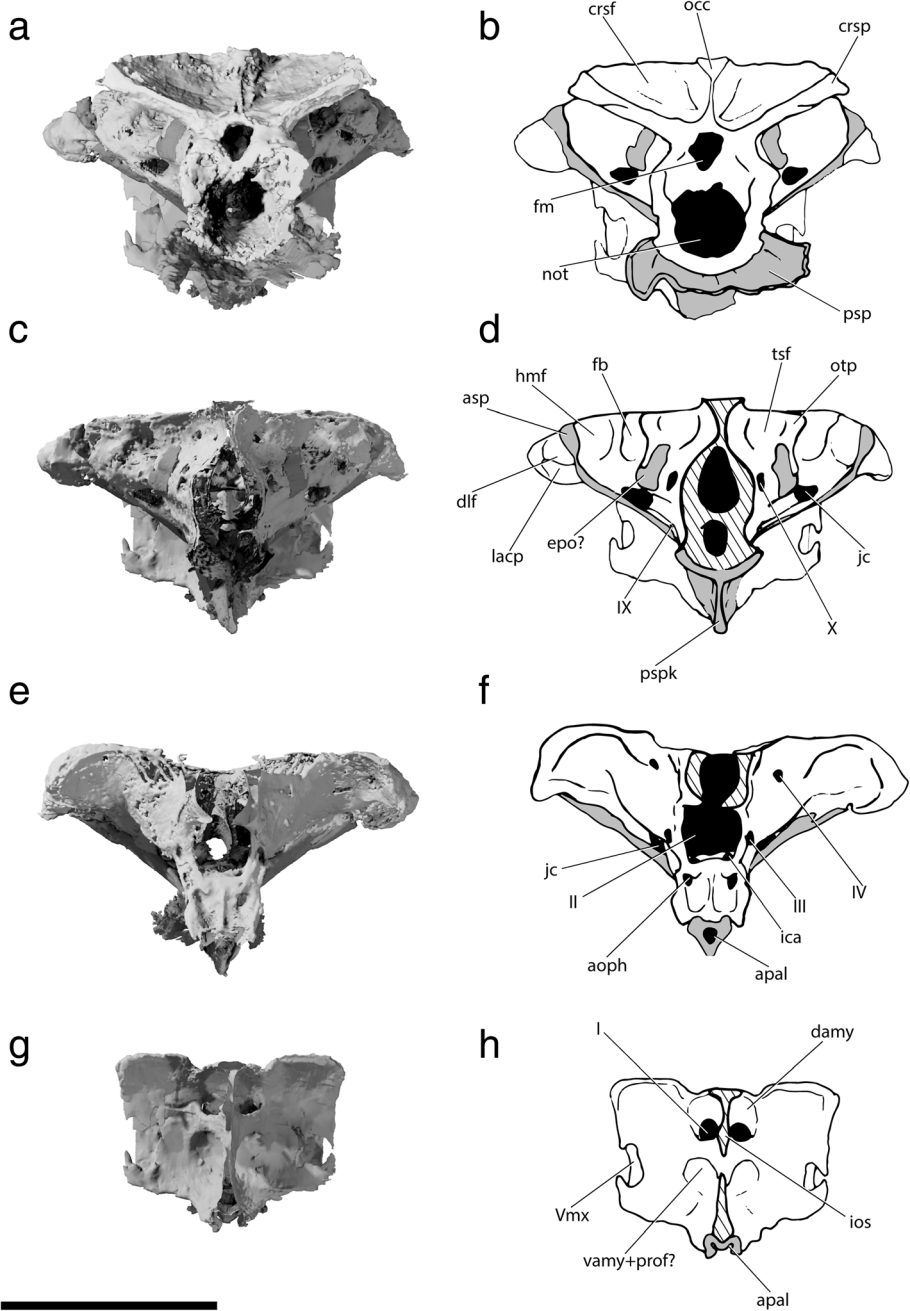
Coronal views of tomographic renderings of different regions of the braincase and parasphenoid of †*Saurichthys* sp. (NHMD_157546_A); **a** posterior view of occipital region; **b** interpretative drawing of **a**; **c** posterior view of otic region; **d** interpretative drawing of **c**; **e** anterior view of orbitotemporal region; **f** interpretative drawing of **e**; **g** posterior view of ethmoidal region; **h** interpretative drawing of **g**; gray shade indicates elements of dermal origin. Abbreviations: **I,** olfactory nerve; **II,** optic nerve; **III,** oculomotor nerve; **IV,** trochlear nerve; **Vmx,** maxillary ramus of trigeminal nerve; **IX,** glossopharyngeal nerve; **X,** vagus nerve; **apal,** palatine artery (parabasal canal); **aps,** pseudobranchial artery; **asc,** anterior semicircular canal; **asp,** ascending process of parasphenoid; **crsf,** craniospinal fossa; **crsp,** craniospinal process; **damy,** dorsal anterior myodome; **dlf,** likely origin of dilatator and/or hyomandibular protractor muscles; **epo?,** epiotic-like ossification; **fb,** fossa bridgei; **hmf,** hyomandibular facet; **ica,** ascending branch of internal carotid artery; **ios,** interorbital septum; **jc,** jugular canal; **lacp,** potential origin of levator arcus palatini muscle; **occ,** occipital crest; **oph,** opthalmic artery; **otp,** otic process; **psp,** parasphenoid; **pspk,** parasphenoid keel; **tsf,** tectosynotic fossa; **vamy + prof?,** ventral anterior myodome and potential course of profundus nerve. Scale bar equals 1 cm

**Fig. 6:**
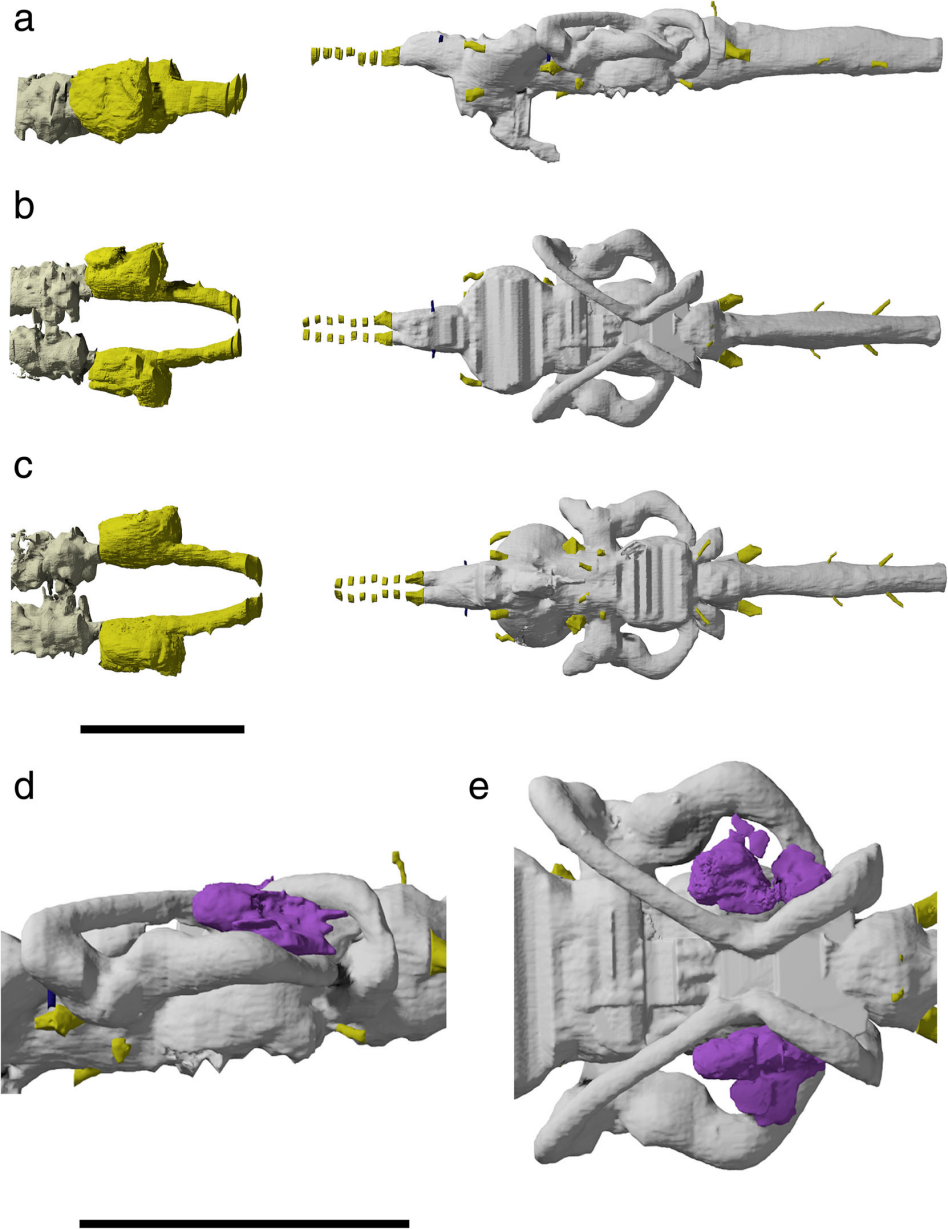
Tomographic renderings of brain, osseus labyrinth and nasobasal canal endocasts of †*Saurichthys* sp. (NHMD_157546_A); **a** left lateral view; **b** dorsal view; **c** ventral view; **d** left lateral closeup of bony labyrinth and intramural diverticula; **e** closeup of dorsal view of bony labyrinth and intramural diverticula. Origin of major cranial nerve canals in yellow, canals for veins in blue, intramural diverticula in purple, nasobasal canals in beige. Scale bars equal 1 cm

**Fig. 7:**
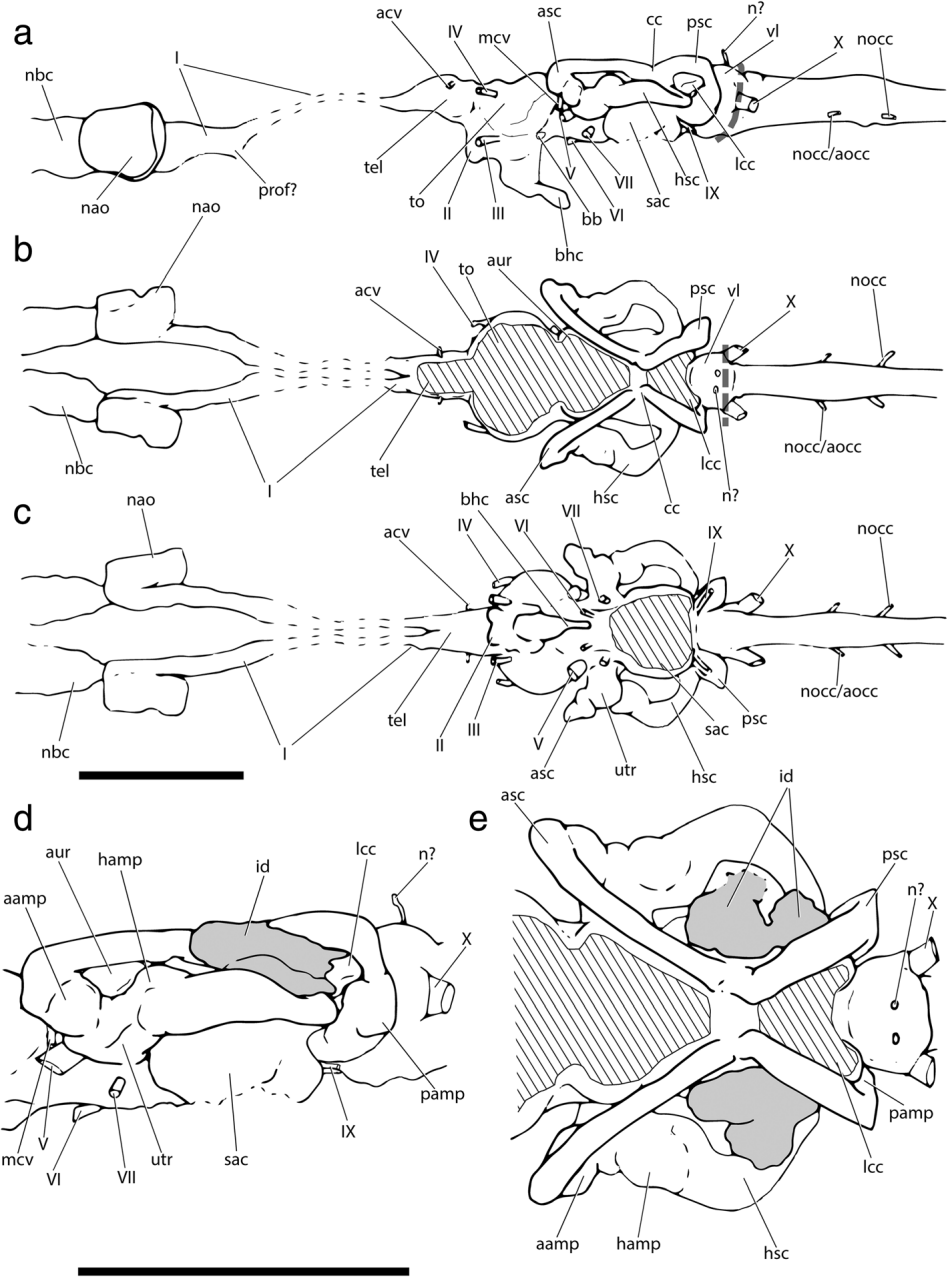
Interpretative drawings of brain, osseus labyrinth and nasobasal canal endocasts of †*Saurichthys* sp. (NHMD_157546_A); **a** left lateral view; **b** dorsal view; **c** ventral view; **d** left lateral closeup of bony labyrinth and intramural diverticula; **e** closeup of dorsal view of bony labyrinth and intramural diverticula. Dashed gray line indicates cryptic oticooccipital fissure. Abbreviations: **I,** olfactory nerve; **II,** optic nerve; **III,** oculomotor nerve; **IV,** trochlear nerve; **V,** trigeminal nerve; **VI,** abducens nerve; **VII;** facial nerve; **IX,** glossopharyngeal nerve; **X,** vagus nerve; **aamp,** ampulla of anterior semicircular canal; **acv,** anterior cerebral vein; **asc,** anterior semicircular canal; **aur,** cerebellar auricle; **bb,** bony bar (dorsum sellae); **bhc,** buccohypophyseal canal; **cc,** crus communis; **hamp,** ampulla of horizontal semicircular canal; **hsc,** horizontal semicircular canal; **id,** intramural diverticulum; **lcc,** lateral cranial canal; **mcv,** mid-cerebral vein; **n?,** putative dorsal ramus of IX or X; **nbc,** nasobasal canal; **nocc;** spinooccipital nerve; **nocc/aocc;** spinooccipital nerve or occipital artery; **pamp,** ampulla of posterior semicircular canal; **prof?,** putative course of profundus nerve; **psc,** posterior semicircular canal; **sac,** saccular recess; **tel,** telencephalon; **to,** optic tectum; **utr,** utricular recess; **vl,** vagal lobe. Scale bars equal 1 cm

The occipital region (Figs. 1, 2, 3, 4, and 5a) is delineated by the craniospinal processes posteriorly (‘crsp’), and the cryptic oticooccipital fissure anteriorly. Despite being externally covered by perichondral bone, the oticooccipital fissure persists as a weakly mineralized belt, forming a break in the perichondral and endochondral lining of the endocavity (Figs. 4a, b, 6a, b, 7a, b; Additional file 3: Figure S1A: ‘otcf’). The oticooccipital fissure begins dorsolaterally, intersects the vagus (‘X’) foramen and extends ventrally to below the level of the saccular recess of the inner ear. The anterodorsal surface of the occipital region is poorly mineralized, but a posterior dorsal fontanelle was likely absent, as evidenced by the presence of dorsally-directed, mineralized canals, tentatively interpreted as passages for the dorsal rami of the vagus (Figs. 6a, b, 7a, b: ‘n’). There are no vestibular fontanelles. The ventral floor of the braincase is weakly mineralized, and the condition of the ventral otic fissure cannot be assessed.

The narrow foramen magnum (‘fm’) is ovoid in cross-section and is the most dorsal of the two openings on the posterior face of the occipital region. The notochordal canal (‘not’) lies ventral to the foramen magnum, and is much wider than the latter and approximately circular in cross-section. No thickened notochordal calcification was observed. The two canals communicate posteriorly through the parachordal notch, which terminates slightly posterior to the level of origin of the craniospinal processes. Anterior to this point, the notochordal canal and the foramen magnum are completely enclosed in bone and separated by a continuous horizontal shelf. The notochordal canal extends until almost the anterior margin of the occipital region, but its radius decreases abruptly anterior to the level of origin of the craniospinal processes. Two small canals issue from the notochordal canal and open laterally on each side of the specimen. An aortic canal is absent.

The prominent craniospinal processes originate from the dorsal half of the occipital region, and expand posterolaterally. The posterior face of each craniospinal process bears a deep craniospinal fossa (‘crsf’). The two fossae are separated on the midline by a shallow occiptal crest (‘occ’), which extends along the dorsal margin of the occipital region and widens anteriorly. The laterodorsal part of the braincase between the craniospinal processes and the otic region bears a paired concavity, which extends anteriorly to the posteromedial surface of the otic region, and is mesial to the otic crest formed by the posterior semicircular canal. We consider this concavity to be an expanded tectosynotic fossa (‘tsf’, see also discussion). This fossa sits adjacent to an expanded muscle attachment shelf on the hyomandibula. A common canal for both roots of the first spinooccipital nerve (Spinooccipitalis α of [29]) opens laterally below the craniospinal process. A canal that transmitted either another spinooccipital nerve (ventral root of the N. Spinooccipitalis z of [29]), or the occipital artery, opens anterior and slightly ventral to the previous canal (‘nocc/aocc’).

##### Remarks

The oticooccipital fissure is externally closed in several actinopterygian taxa, including †*Saurichthys*, †*Amphicentrum*, Cladistia, Chondrostei, living Holostei and crown Teleostei [44, 63, 64]. This contrasts with the open oticooccipital fissure of most Paleozoic–early Mesozoic actinopterygians and early neopterygians [39, 40, 44, 58, 59, 63, 65–69]. The discovery of a cryptic oticooccipital fissure allows, for the first time, the mapping of the boundary between the occipital and otic regions in †saurichthyids.

**Fig. 8:**
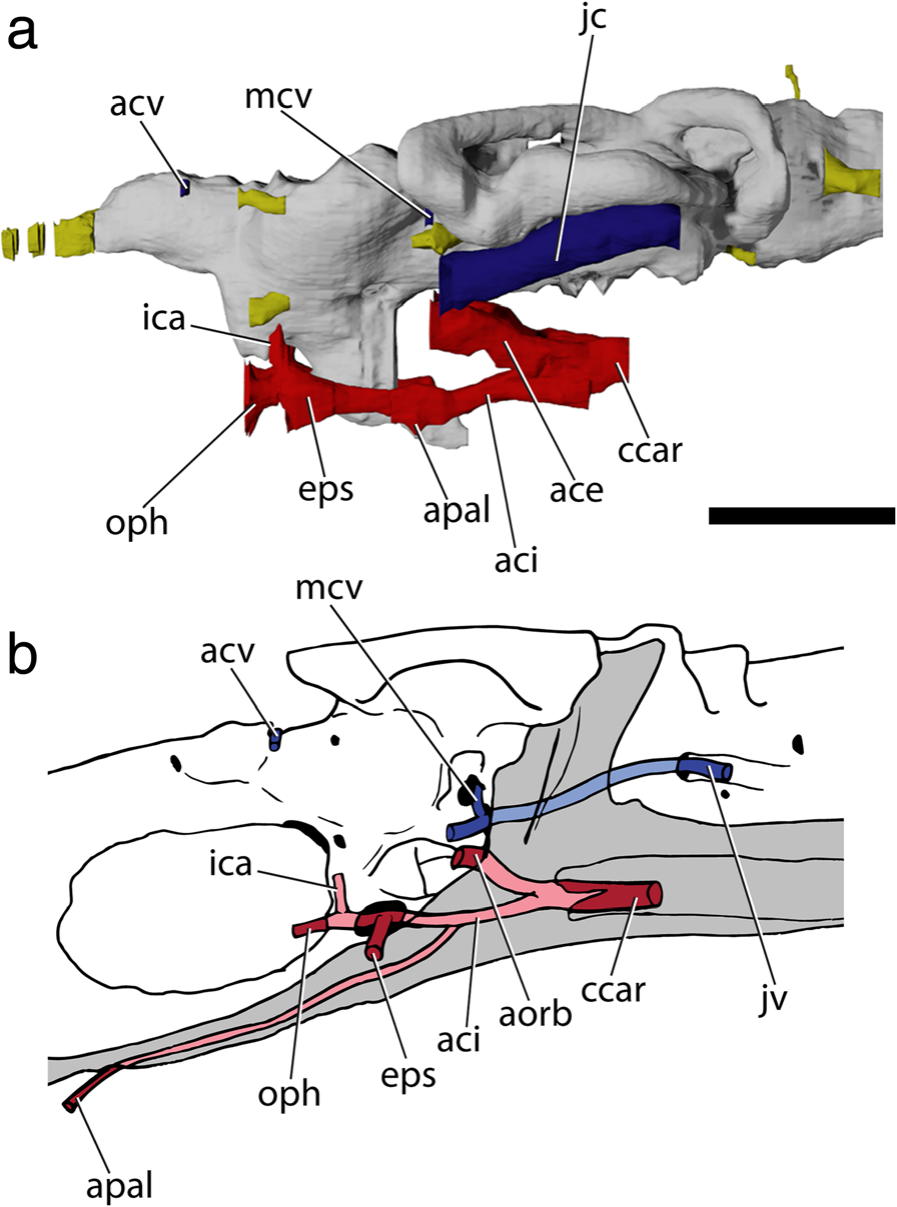
Basicranial circulation of †*Saurichthys* sp. (NHMD_157546_A); **a** digital rendering of brain and osseus labyrinth endocasts, with major cranial nerves in yellow, arterial canals in red and venal canals in blue; **b** Simplified schematic of skull in lateroventral view showing the passage of major blood vessels. Abbreviations: **aci,** common branch of internal carotid artery; **acv,** anterior cerebral vein; **aorb,** orbital artery; **apal,** palatine artery (parabasal canal); **eps,** efferent pseudobranchial artery; **ccar,** common carotids; **ica,** ascending branch of internal carotid; **jc,** jugular canal; **jv,** jugular vein; **mcv,** mid-cerebral vein; **oph,** ophthalmic artery. Scale bars equal 0.5 cm

†*Saurichthys* resembles living neopterygians in the sense that the dorsal aorta and lateral dorsal aortae extend ventral to the elongated posterior stalk of the parasphenoid (e.g., [70, 71]). Presumed similarities in vascularization between †*Saurichthys* and acipenseriforms are often emphasized in character descriptions [16, 29, 32]. However, there are notable differences between the latter two groups. The most conspicuous difference can be found in the course of the lateral dorsal aortae, which bifurcate posterior to the occiput and extend ventral to the parasphenoid in †saurichthyids (Figs. 4, 5, 8). In acipenserifoms they are embedded in a groove on the ventral surface of the first few abdominal vertebrae, extend mostly dorsal to the parasphenoid, are flanked by the deep parasphenoid notch, and bifurcate anterior to the occiput [72, 73] (Additional file 3: Figure S3, S4). In some specimens of *Acipenser*, the dorsal aorta can be embedded in a short aortic canal immediately before it bifurcates to efferent branchial arteries (Additional file 3: Figure S4: ‘abreff’).

The occipital region of †saurichthyids and most non-neopterygians [6, 29, 44, 58, 59, 63, 65–69, 74, 75] bears craniospinal processes. Based on comparison with acipenseriforms [73, 75, 76], the only living examples exhibiting craniospinal processes, the latter processes form fossae that must have accommodated the first few epaxial muscle segments [44]. An expanded, laterally-facing tectosynotic fossa is present in acipenseriforms (Additional file 3: Figure S2), and it hosts the origin of the hyoid and opercular retractors and the branchial levator muscles [73, 76, 77]. We hypothesize a similar arrangement in †*Saurichthys*, based on fossa orientation and the arrangement of the hyomandibula. Due to difficulties in mapping different regions of the neurocranium in adult acipenseriforms, it is unclear whether the tectosynotic fossa crosses to the occipital region as it does in †*Saurichthys*.

**Fig. 9:**
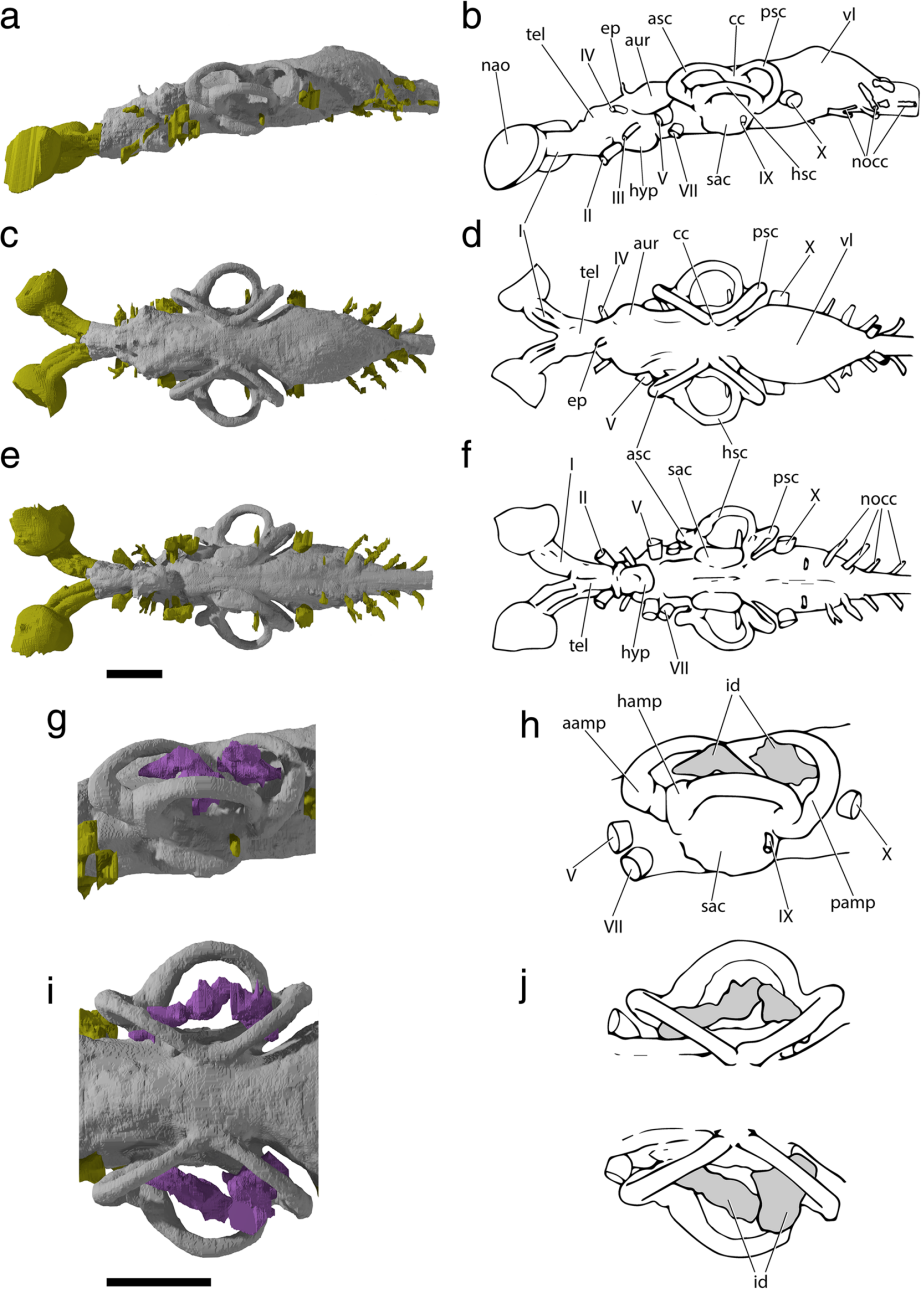
Brain and labyrinth endocast anatomy of *Acipenser brevirostrum* (FMNH 113538). **a** lateral view; **b** interpretative drawing of **a**; **c** dorsal view; **d** interpretative drawing of **c**; **e** ventral view; **f** interpretative drawing of **e**; **g** left lateral closeup of bony labyrinth and intramural diverticula; **h** interpretative drawing of **g**; **i** closeup of dorsal view of bony labyrinth and intramural diverticula; **j** interpretative drawing of ***I****. major* cranial nerves in yellow, intramural diverticula in purple. Abbreviations: **I,** olfactory nerve; **II,** optic nerve; **III,** oculomotor nerve; **IV,** trochlear nerve; **V,** trigeminal nerve; **VII,** facial nerve; **IX,** glossopharyngeal nerve; **X,** vagus nerve; **asc,** anterior semicircular canal; **aur,** cerebellar auricle; **cc,** crus communis; **ep,** epiphysis; **hsc,** horizontal semicircular canal; **hyp,** hypophyseal chamber; **nao,** narial opening; **nocc,** spinooccipital nerve; **psc,** posterior semicircular canal; **sac,** saccular recess; **tel,** telencephalon; **vl,** vagal lobe. Scale bar equals 2 cm

†*Kansasiella* and †*Saurichthys* are reconstructed with two spinooccipital foramina [29, 65], whereas only one is present in the lateral occipital region of †*Mimipiscis*, †*Pteronisculus*, †*Australosomus*, †*Kentuckia* and †*Lawrenciella* [44, 58, 59, 67, 78]. Yet, such attributions in fossils should be treated with caution, since these foramina could also have transmitted blood vessels. Acipenseriforms exhibit three spinooccipital nerves [73, 77]; Fig. 9, Additional file 3: Figure S3), although a fourth, blind-ending canal is present anterior to the remaining spinooccipital nerves in the endocast of *Acipenser* we examined. *Erpetoichthys*, *Amia* and gars exhibit two spinooccipital nerve foramina, while *Polypterus* shows three [70, 75, 79–81].

#### Otic and orbitotemporal regions

The otic region (Figs. 3, 4, 5c, d) includes the portion of the braincase enclosing the bony labyrinth, the anterior tip of which terminates slightly beyond the level of the broad postorbital process (‘porp’). Ventrally, it extends up to the posteroventral margin of the myodome. The orbitotemporal region extends anteriorly up to the posterior wall of the ethmoidal region. There is no clear boundary between the otic and orbitotemporal regions, so they are considered collectively here. The flat dorsal surface of the otic and orbitotemporal regions is poorly mineralized and not well resolved in our scan. The anterior fontanelle appears extensive, but is tentatively reconstructed. The posterior tip of the postorbital process is the widest part of the braincase, but the width decreases abruptly at the level of the orbits.

In lateral view, the anterior portion of the tectosynotic fossa (Figs. 3a, b, 4a, b, 5c, d) is bounded medially by the occipital crest and laterally by the process containing the posterior semicircular canal (‘otp’). Anterolaterally to the tectosynotic fossa, and roughly constrained by the planes of the three semicircular canals, there is a depressed area, which corresponds to the fossa bridgei (‘fb’). The latter exhibits a deep posterior subdivision, which opens posterolaterally, towards the anteromedial surface of the hyomandibula. The anterior and medial walls of the posterior subdivision of the fossa bridgei connect to perichondrally-lined, intramural diverticula in the braincase (Figs. 6d, e, 7d, e: ‘id’). The posterior diverticulum extends medially towards the cranial cavity, while the anterior one extends anteriorly, reaching past the level of the crus communis. The posterior opening of the fossa bridgei is succeeded laterally by a posterolaterally facing, subtriangular, shallow hyomandibular facet. The hyomandibular facet (‘hmf’) is separated from the broad postorbital process by the ascending process of the parasphenoid (‘asp’). The presence of an enclosed spiracular canal could not be verified due to limited contrast in tomograms, and may have been absent. Anterolaterally, the tip of the ascending process of the parasphenoid gives way to a shallow, posteriorly facing dilatator fossa (‘dlf’) on the caudal surface of the postorbital process, which is likely to have hosted the hyomandibular protractor muscle. The levator arcus palatini likely originated from the broad fossa of the ventral part of the postorbital process.

A foramen for the vagus nerve, and potentially the posterior cerebral vein [29], opens on the posterolateral surface of the otic region. The jugular vein extended through a depression beginning immediately ventral to the exit of the vagus. The glossopharyngeal nerve (‘IX’) exited ventral to the jugular depression. The jugular depression continues anteriorly and slightly dorsally to become the jugular canal (‘jc’; trigeminofacialis chamber in [29]), which pierces the lateral commissure and opens on the posteroventral part of the orbitotemporal region. The canal for the facial nerve (‘VII’) opens into the jugular canal through its posteromedial wall. Dorsomedial to the anterior opening of the jugular canal, there is a large opening for the trigeminal nerve (‘V’), and potentially the profundus and the anterior trunks of the facial nerve. Slightly anterodorsal to the latter, there is a vertical canal for the mid-cerebral vein. A lateral pillar (alisphenoid pedicel) is absent.

The external (Fig. 8: ‘ace’) and internal carotid arteries (‘aci’) split from the common carotids (‘ccar’) upon entering the parasphenoid. Then, they extended anterodorsally along a canal formed between the parasphenoid and the braincase, to merge with the anterior opening of the jugular canal. From that point, each external carotid likely bifurcated to a posterior (hyomandibular, not reconstructed) and an anterior (orbital, ‘aorb’) branch. The internal carotids continued anteriorly along parabasal canals below the lateral openings of the posterior myodome. At this point the palatine artery (‘apal’) branched off and continued its anterior course through a parabasal canal, completely enclosed within the parasphenoid. The remaining internal carotid branches enter the ventral part of the orbital region. A foramen for the efferent pseudobranchial artery (‘eps’) opens on each side, anteroventrally to the anterior margin of the posterior myodome (Additional file 3: Figure S2A). Anterior to the pseudobranchial foramina, each internal carotid bifurcated into the (greater) ophthalmic artery (‘oph’) and an ascending (‘ci’) branch. The ophthalmic arteries extended anteriorly and exited the braincase, forming troughs immediately ventral to the optic foramen, while the ascending branches enter the brain cavity through the lower margin of the optic foramen.

The median posterior myodome (Figs. 3a, 4a: ‘pmy’) is well developed and situated in front of the ascending process of the parasphenoid, anteroventral to the anterior opening of the jugular canal. The anterior wall of the posterior myodome is in communication with the hypophyseal chamber. The course of the pituitary vein could not be observed. It was likely confluent with the hypophyseal chamber or with the paired canal for the abducens nerve (‘VI’), which opens on the roof of the myodome. There is no basipterygoid process. The cranial cavity becomes markedly convex above the myodome to accommodate the expanded optic tecta. A foramen for the trochlear nerve (‘IV’) is located on the dorsal margin of this convexity. A foramen for the anterior cerebral vein (‘acv’) opens anterior to the trochlear foramen, followed anteroventrally by the foramen for the olfactory nerve (‘I’), on each side of the specimen. The anterior face of the orbitotemporal region is dominated by the median foramen for the optic nerve (‘II’), which opens onto the posterior margin of the large interorbital fenestra. The optic foramen is flanked on each side by the foramina for the oculomotor nerve (‘III’). The canals for the (greater) ophthalmic artery and potentially the exit of the anterior cerebral artery opens ventral to the optic foramen (Figs. 5e, f, 8: ‘oph’). The olfactory nerve exited the cranial cavity above the mid-length of the optic fenestra. Upon exiting the braincase, each tract extended in a shallow groove along the dorsal margin of the interobital fenestra (‘iof’) to enter the ethmoidal region through a paired, funnel-shaped foramen. The interorbital fenestra is greatly enlarged, reducing the thin interorbital septum to its anterior and anterodorsal parts.

##### Remarks

An expanded anterior fontanelle is present in most post-Devonian non-neopterygian actinopterygians in which the condition can be assessed [58, 59, 65, 67, 79, 82]. A fossa bridgei is present in most Carboniferous or younger actinopterygians, but unlike in †*Saurichthys*, it is posteriorly delimited by an endochondral wall [29, 44, 58, 59, 63, 67, 83]. Based on orientation and proximity to the hyomandibula, we hypothesize that the elimination of the posterior wall of the fossa bridgei in †*Saurichthys* is linked to the attachment of the hyomandibular retractor muscle, and not to the attachment of epaxial musculature, which is the case in many neopterygians [63]. The opening of intramural diverticula in the fossa bridgei is observed in †*Kansasiella* [65], †*Saurichthys* (Figs. 6d, e, 7d, e) and *Acipenser* (Fig. 9g–j, Additional file 3: Figure S3). In the latter two taxa, where the condition can now be assessed, the diverticula are subdivided into two distinct portions on each side and show a similar arrangement. However, the contact between the two portions is contained within the braincase in †*Saurichthys*, but happens in the fossa bridgei in *Acipenser*. In *Polyodon*, but also in †*Pteronisculus* and †*Boreosomus*, the lateral cranial canal opens in the floor of the fossa bridgei at a topologically equivalent position [58, 59, 84]. This topological correspondence could be suggestive of homology between intramural diverticula and parts of the lateral cranial canal.

The position of the hyomandibular facet of †*Saurichthys*, dorsal to the jugular canal, is reminiscent of the condition seen in Devonian actinopterygians [44, 69]. However in the latter, the facet is oriented laterally, rather than posteriorly as in †*Saurichthys*. The posterior orientation of the †saurichthyid dilatator fossa, which in analogy with modern taxa must have carried the hyoid protractor muscle [73, 76, 77], is similar to that of gars, likely reflecting the elongate geometry of their skulls. It differs from that of most neopterygians [63], in both its position (anterior to hyomandibular facet versus anterodorsal in most neopterygians) and orientation (posterior versus lateral in most neopterygians).

The jugular canal of †*Saurichthys* resembles that of †*Kansasiella*, differing from that of †*Mimipiscis*, †*Lawrenciella*, *Acipenser*, and several fossil holosteans in not having the orbital artery entering the jugular canal posteriorly, but rather entering it ventrally along its course [44, 63, 65, 67]. †*Saurichthys* differs from †*Pteronisculus*, †*Kentuckia*, the Greenland †‘*Perleidus*’ and early teleosts in not exhibiting separate foramina for the exit of the hyomandibular trunk of the facial nerve above the posterior exit of the jugular canal [58, 63, 78]. The profundus nerves also form separate foramina, dorsal–dorsomedial to the anterior opening of the jugular canal in many fossil actinopterygians [44, 58, 63], but likely share the same exit with other nerves in †*Saurichthys*, †*Australosomus*, polypterids, and acipenseriforms [59, 73, 79]. The presence of a median posterior myodome in †*Saurichthys* resembles the condition in †*Lawrenciella*, †*Pteronisculus*, †*Boreosomus*, †*Australosomus*, and neopterygians [44, 58, 59, 63, 67]. †*Yelangichthys*, however, exhibits a paired posterior myodome [32]. In stem osteichthyans, sarcopterygians, †*Mimipiscis*, *Polypterus*, and acipenseriforms the posterior myodome is absent [18, 44, 74, 79, 85, 86]; (Additional file 3: Figure S3).

An endochondral or dermal basipterygoid process is absent in acipenseriforms, †*Saurichthys*, †*Australosomus*, extant polypterids, †*Caturus*, *Amia*, and likely also in †*Birgeria* [6, 7, 29, 59, 74, 79, 83]. A gentle thickening formed by the canal of the pseudobranchial artery was described as an endochondral basipterygoid process in †*Yelangichthys* [32]. However, its small size and shape contrasts sharply with the well-developed and acute endochondral basipterygoid processes of generalized actinopterygians [44, 58, 65], leading us to also consider it absent.

#### Ethmoidal region

In dorsal view, the ethmoidal region of NHMD_157546_A widens rapidly before tapering again anteriorly, forming the core of the elongate rostrum of †saurichthyids. The posterior face of the ethmoidal region is concave. Near its contact with the postnasal wall, the interorbital septum (‘ios’) exhibits a dorsal and a ventral fenestra, the dorsal (‘damy’) and ventral (‘vamy’) anterior myodomes, which must have accommodated the superior and the inferior oblique muscles of the eyes, respectively. The olfactory nerve tracts enter the ethmoidal region dorsomedially through a funnel-shaped foramen on each side of the interorbital septum. Posteromedially, the two foramina coalesce with the anterodorsal fenestra of the interorbital septum. A pair of canals likely carrying the branches of the profundus nerve and/or the origin of the inferior oblique muscle (‘vamy+prof?’) merges ventrally with the olfactory canals near their point of entry in the ethmoidal region. No other foramina are present on the posterior wall of the ethmoidal region.

The dorsal face of the ethmoidal region is mostly flat, bearing two shallow, longitudinal depressions on each side (Figs. 3b, 4b), which must have transported the superficial ophthalmic ramus and the ramus ophthalmicus lateralis of the trigeminal nerve (‘Vopts’) and the supraorbital sensory canal (‘soc’). The two external nares open laterally (‘nao’). A groove extends along the lateroventral margin of the ethmoidal region, probably hosting the maxillary trunk of the trigeminal nerve (‘Vmx’). The ventral ethmoidal surface bears a median longitudinal ridge to which the parasphenoid attaches. This ridge is flanked by a shallow longitudinal depression on each side. A shallow, V-shaped fossa for the articulation of the autopalatine (‘auf’) is present on both posterolateral margins of the ventral ethmoidal region (Figs. 3c, 4c). The endoskeletal anatomy of the rostrum is not well resolved in our scan, but we note the presence of wide nasobasal canals (Figs. 6, 7: ‘nbc’) beginning at the anterior margin of the nasal cavity and extending anteriorly along the preserved length of the rostrum. The area immediately posterior to the nasal cavities is weakly mineralized, exhibiting asymmetrical, pocket-like spaces.

##### Remarks

See remarks section for †*Saurichthys nepalensis* below.

#### Brain and inner ear endocasts

The roof of the brain endocast (Figs. 6, 7) and the floor of the saccular recess (‘sac’) of NHMD_157546_A are incompletely mineralized and cannot be reconstructed. The remainder of the endocast shows increased anatomical complexity (non tube-like), reflecting the position and relative development of different sensory centers, unlike in e.g., teleosts, where there is almost no correspondence between endocast and brain anatomy [18, 87]. The brain endocast is markedly elongate and narrow in dorsal view, except in the area of the optic tectum (‘to’). Anteriorly, it terminates above the mid-length of the interorbital fenestra. The different sensory regions appear serially arranged.

The rhombencephalic region, including the cerebellum, constitutes more than two thirds of the endocast length, reaching anterior to the crus communis (‘cc’). A spinooccipital nerve canal (‘nocc’) and a canal for an additional spinooccipital nerve or the occipital artery stem from posterior to anterior on the base of the rhombencephalon, on each side of the specimen. Anteriorly, the rhombencephalic region gains height and leads to a dorsally bulging globular structure between the posterior semicircular canals. The vagus stems from the base of this globular structure, which we thus interpret as the vagal lobe (‘vl’) of the rhombencephalon (e.g., [88]). Two mineralized canals (‘n?’), one on each side, stem from the dorsal surface of the vagal lobe, and could be associated with dorsal rami of the IX or X cranial nerves. Their dissociation from the osseus labyrinth endocast (sinus superior) precludes their attribution to endolymphatic ducts. Immediately anterior to the vagal lobe, the lateral cranial canal (‘lcc’) forms a laterally-bulging, blind-ended diverticulum, terminating medially to the loop of the posterior semicircular canal (‘psc’). Anterior to the lateral cranial canal, the brain endocast is markedly constricted by the overarching development of the bony labyrinth, whose crura communes converge medially, above the hindbrain part of the endocast.

The cerebellar auricles (‘aur’) are poorly developed and expand laterally, in front of the crura communes, being dorsally restricted by the anterior semicircular canals (‘asc’). The facial nerve exits below the junction between the anterior and the horizontal semicircular canals, to enter the jugular canal. The stem of the abducens nerve exits from the ventral surface of the endocast, at the level of the anterior tip of the cerebellum, and enters the posterior myodome. The trigeminal nerve exits at the same level, at about mid-height of the brain endocast. A downward-facing canal for the median cerebral vein (‘mcv’) is situated at the boundary between each cerebellar auricle and the optic tectum.

The optic tectum is well-developed laterally. The trochlear nerve branches off anteriorly from the anterolateral surface of the optic tectum. Ventrally, there is no differentiation between the latter and the diencephalon. The posterior margin of the hypophyseal recess is not mineralized; hence, the extent of the saccus vasculosus cannot be assessed. The dorsum sellae is reduced to a bony bar (‘bb’), separating the hypophyseal recess from the overlying mesencephalon. The buccohypophyseal canal (‘bhc’) extends posteroventrally through the parasphenoid, but the course of the pituitary vein is not observable. The optic nerve exits through an enlarged median optic foramen below the boundary between the optic tectum and the telencephalon (‘tel’). The posterior boundary of the telencephalon is marked by a gentle constriction, separating it from the bulge of the tectal and the underlying diencephalic regions. The telencephalon is short. The olfactory bulbs stem from the anteroventral part of the telencephalon. The two tracts of the olfactory nerve originate at the anterior tip of the telencephalon and are well separated along their length by the interorbital septum, being uninvested for much of the course through the orbit. They diverge laterally upon entering the ethmoidal region, leading to sizable nasal cavities.

The bony labyrinth of NHMD_157546_A is well ossified, apart from the ventral part of the saccular recess. Medially, in the absence of an ossified boundary, it is continuous with the rest of the endocranial cavity. The semicircular canals are large and robust, with the posterior and especially the anterior ones being dorsoventrally shallow. This compression is natural and not due to post-mortem distortion. The posterior semicircular canal is the shortest of the three and is somewhat dorsoventrally flattened. A small constriction precedes the sizable posterior ampulla (‘pamp’). The anterior canal is the largest of the three; it is flattened dorsoventrally, forming a sharp anterior angle. The region around the anterior ampulla (‘aamp’) is thicker and is separated by both the dorsal part of the canal and the utricular recess by means of gentle constrictions. The utriculus (‘utr’) appears as a lateral projection of the endocast and is somewhat flat rather than globular. The ampulla of the horizontal canal (‘hamp’) extends dorsal to the utriculus. The horizontal canal (‘hsc’) forms a hemi-elliptical curve. It enters the cranial cavity slightly ventral to the level of the posterior ampulla. The sinus superior is short. The saccular recess is laterally convex, but its full ventral extent is not visible due to the absence of mineralization. The stem of the glossopharyngeal nerve is situated on the boundary between the sacculus and the ampullary space of the posterior semicircular canal.

##### Remarks

The anatomy of non-neopterygian actinopterygian brain endocasts is thought to mirror that of the contained soft tissues [18, 87], due to the presence of only a single layer of meningeal tissue separating the latter from the braincase [89] Descriptions of partial brain and/or inner ear endocasts were provided for †*Saurichthys ornatus*, †*S. elongatus*, †*S. hamiltoni* and †*S. minimahleri* [29, 90]. The digital endocast presented here is the first to depict the brain and inner ear cavities of the same individual in all views, and conveys information missing in previous studies. This is a valuable addition to the small number of fossil actinopterygian endocasts described to date (see supplement to [87] and [3, 69, 82, 91] for more recent entries). Surprisingly, endocast information is still lacking for extant non-teleostean actinopterygians, with the exception of *Acipenser brevirostrum* (Fig. 9) and *Erpetoichthys* (partial endocast in supplement to [12]).

In most Paleozoic–early Mesozoic species, the area of the vagal lobe is confluent with the posterior dorsal fontanelle. Nevertheless, a prominent vagal swelling, like that of †*Saurichthys*, was reconstructed for †*Lawrenciella*, †*Kansasiella*, and †*Pteronisculus* [58, 65, 67], and is also present in the endocast of *Acipenser* (Fig. 9). In life, however, this part of the brain of sturgeons is narrow and rod shaped, and does not fill the vagal space [73]:fig. 270a. A pronounced mismatch between endocast and brain morphology at the level of the vagal lobe has also been demonstrated for the lungfish *Neoceratodus* [92], suggesting that paleoneurological information from this region of the endocast of bony fishes should be treated with caution.

Primitively for actinopterygians, the lateral cranial canal was a blind-ending pocket extending from the endocavity through the posterior semicircular canal [3, 44, 65, 67, 69], and a similar arrangement is also seen in †*Saurichthys* and possibly in extant polypterids [12]. In *Acipenser*, the lateral cranial canal is absent (Fig. 9), but in *Polyodon* it is present and extends laterally through the loop of the posterior semicircular canal, to connect with the fossa bridgei [84]. This is suggestive of increased variation of this feature even amongst closely related taxa. In fossil holosteans and stem teleosts, the lateral cranial canal wraps around the sinus superior to form an additional connection with the endocavity, through the loop of the anterior semicircular canal [63, 83, 91]. The lateral cranial canal is lost in extant holosteans and crown teleosts [63, 83]. The function of the lateral cranial canal is unknown, but an association with the lateral development of an epimyelencephalic hemopoetic organ has been suggested [85, 93].

In †*Saurichthys*, the cerebellum appears small, due to the extensive development of the optic tecta. An increase in tectal development relative to the cerebellum is also commonly seen in neopterygians, and is particularly pronounced in teleosts [91, 94]. Primitively, in the endocasts of †*Mimipiscis*, †*Raynerius*, †*Pteronisculus*, and to a lesser extent in those of †*Kansasiella* and †*Lawrenciella*, the cerebellar auricles are broader than the optic tecta [58, 65, 69, 87]. In *Acipenser*, the auricular space is also broader than the tectal space (Fig. 9), but the optic tectum remains poorly differentiated, despite a clear separation between the two sensory centers in the actual brain [73]. In *Erpetoichthys*, the auricles are poorly differentiated, but still broader than the optic tecta [12]:ext. fig. 9. A poor differentiation of tectal and auricular spaces is also seen in †*Boreosomus* [58]. In †*Saurichthys*, †*Mimipiscis* [87]*,* and †*Pteronisculus* [58], the middle cerebral vein enters the endocast below the cerebellar auricles. In †*Kansasiella* and †*Lawrenciella*, it reaches the dorsolateral surface of the auricles [65, 67, 82]. The arrangement of this vessel is unknown in other taxa. The stem of the trochlear nerve lies in a dorsolateral position on the optic tectum in †*Saurichthys*, †*Mimipiscis* [87], †*Pteronisculus* [58], and, albeit less so, in †*Kentuckia* [87]. In †*Kansasiella*, †*Lawrenciella*, and †*Mesopoma*, it extends from the ventrolateral part of the optic tectum [18, 65, 67, 82].

A well-developed hypophyseal chamber with a clearly differentiated and prominent saccus vasculosus and a ventrally-to-anteroventrally directed buccohypophyseal duct characterize all known Paleozoic actinopterygians, as well as †*Pteronisculus* and †*Australosomus* [18, 58, 59, 65, 67, 82, 87]. †*Saurichthys* shares with sturgeons and bichirs a posteroventrally directed hypophyseal void, differing from that of other non-neopterygian actinopterygians [18, 94]. In neopterygians, the hypophyseal chamber is almost vertical, but the space of the saccus vasculosus is reduced [39, 91]. A rod-like bony bar, which likely corresponds to the dorsum sellae, drives laterally through the endocast*,* above the saccus vasculosus. This is not seen in any actinopterygian other than †*Saurichthys*.

The olfactory bulbs are merged with the telencephalon in the endocast of †*Mimipiscis* [87], *Polypterus*, and *Acipenser* [18, 94], but are better marked by a dorsal to lateral constriction in †*Saurichthys*, †*Kansasiella*, †*Lawrenciella*, †*Mesopoma*, and extant neopterygians [18, 65, 67, 82, 94]. Primitively, the olfactory nerves are not carried in a single tract, with paired tracts present in actinopterygian outgroups (e.g., [85] and also in †*Mimipiscis* [87]). This condition re-evolved in acipenseriforms (Fig. 9). †*Saurichthys* also shows distinct olfactory tracts, but these are carried in shallow grooves on the lateral surface of the interorbital septum, as in gars (pers. obs. on PIMUZ A/I 4171a). In most Paleozoic–Triassic actinopterygians and *Amia*, the olfactory tracts are transmitted to the ethmoidal region via a median endochondral tube [39, 58, 59, 65, 67, 70, 82, 87, 91].

The overall morphology of the osseus labyrinth of †*Saurichthys* is broadly similar to that of generalized non-neopterygian actinopterygians [87], with a few notable modifications. The large degree of medial convergence of the crura communes is the most distinct feature of the osseus labyrinth of †*Saurichthys*. A reduced level of crural convergence, but a greater degree of superimposition on the brain cavity, occurs in †*Meemania*, †*Mimipiscis* and to a lesser degree in †*Raynerius* [3, 69, 87]. Crural convergence is seen in some neopterygians [91] and polypterids [12], but superimposition is typically absent in other actinopterygians [12, 18, 39, 58, 59, 65, 67, 87, 91]; Fig. 9). In sarcopterygians [95], and less so in †*Mimipiscis*, †*Kentuckia* [87], †*Pteronisculus* [58] and fossil neopterygians [91], there is a ventrally expanded utricular recess. This feature is less pronounced in †*Saurichthys*, polypterids [12], *Acipenser* (Fig. 9) and in other non-neopterygians [59, 67].

#### Parasphenoid and associated dermal bones

The parasphenoid of †*Saurichthys* is cross-shaped in ventral view (Figs. 3c, d, 4c, d), bearing a well-developed posterior stalk that underlies the occipital region and projects posterior to the braincase. The posterior margin of the parasphenoid is notched at the midline, presumably for the passage of the aorta, although the exact shape is obscured due to breakage. Ventrally, there is a prominent median keel (‘pspk’) that extends from slightly anterior to the posterior notch to the level of the ascending processes, where the foramina for the passage of the common carotids (‘ccar’) into the braincase are located. Anterior to the foramina for the common carotids, the keel of the parasphenoid blends gently into the convex ventral surface of the anterior process of the bone. The ventral keel of the parasphenoid is laterally concave on both sides, marking the external course of the common carotids. The branching of the common carotids from the dorsal aorta must have occurred immediately posterior to the ventral keel. The ascending processes (‘asp’) of the parasphenoid extend dorsally and posteriorly, passing over the lateral commissure, to terminate anterolateral to the hyomandibular facets, near the dorsal margin of the braincase. The lateral surface of each ascending process bears a spiracular groove (‘spig’). The anterior process of the parasphenoid is narrower than the posterior one, but is elongate; it can be followed anteriorly all the way below the preserved part of the ethmoidal region, where it overlies the vomers. The buccohypophyseal canal (‘bhc’) opens on the ventral keel of the parasphenoid. A median parasphenoid canal runs through the buccohypophyseal canal, reaching the level of the anterior margin of the interorbital fenestra, where it opens ventrally (‘apal’). This canal must have accommodated the palatine branch of the internal carotid artery and we consider it to be homologous with the parabasal canals of other actinopterygians. A small patch of tiny teeth occurs on the parasphenoid, slightly anterior to the palatine opening.

The paired vomers are elongate and underlie the parasphenoid. Their posterior tips lie slightly rostral to the anterior margin of the orbit, whereas their anterior tips could not be located due to breakage. Each vomer forms an elongate toothplate that bears numerous tiny teeth. The two vomers seem to form a midline suture, whose posterior end is located at the level of the anterior margin of the anterior narial opening. No teeth are observed along the suture line.

##### Remarks

The well-developed posterior stalk as well as the high ascending processes distinguish the parasphenoid of †*Saurichthys* from the primitive actinopterygian condition, exemplified by the lozenge-shaped parasphenoid of †*Raynerius* and †*Mimipiscis* [44, 69]. Ascending processes are typically more developed in post-Devonian actinopterygians, but in many generalized forms the posterior stem still stops short of the occipital region and rarely underlies the ventral otic fissure [39, 40, 44, 52, 58, 59, 67, 96, 97]. The parasphenoid crosses the ventral otic fissure in several Carboniferous and younger actinopterygians, e.g., in †*Amphicentrum*, †*Eurynotus*, †*Sphaerolepis*, †*Errolichthys*, †*Birgeria*, and early neopterygians like †*Watsonulus*, whereas in polypterids and most neopterygians it reaches the level of, and sutures with, the basioccipital and when this can be assessed incorporated vertebrae [7, 9, 40, 59, 63, 64, 79, 98–100]. At least in Early Triassic †saurichthyids [29] and in sturgeons [73–75], the parasphenoid extends well past the occiput, underlying a variable number of rigidly-connected vertebrae (Additional file 3: Figure S3). As with †*Saurichthys*, the posterior stem of the parasphenoid also bears a notch (albeit deeper) in *Polypterus*, *Acipenser*, *Polyodon*, *Lepisosteus*, *Amia,* as well as in fossils such as †*Amphicentrum*, and †*Birgeria* [6, 7, 9, 29, 59, 64, 79].

A closer comparison between the parasphenoid of †*Saurichthys* and that of *Acipenser* reveals conspicuous differences in basicranial circulation that contradict orthodox hypotheses of a close relationship between the two. The parasphenoid of *Acipenser* lacks the enclosed arterial system [73] seen in †*Saurichthys* (Fig. 8). In *Acipenser*, the two variably present ventral foramina on the posterior process of the parasphenoid serve as the exit of the aortic branch that later gives off the first and second efferent branchial arteries and the common carotids [73](Additional file 3: Figure S4). These foramina have been erroneously homologized with the foramina serving as the entrance for the common carotids in †*Saurichthys* [16]. Like in most sturgeons, the common carotids run and bifurcate below the parasphenoid and enter the neurocranium at different points in *Polyodon* [72]. Furthermore, a buccohypophyseal opening is absent in acipenseriforms, and their anterior parasphenoid process terminates underneath the posterior ethmoidal region, giving way to a pair of edentulous vomers [6, 74]. In some Middle Triassic †saurichthyids from China and Switzerland, as well as in †*Saurorhynchus*, the efferent pseudobranchial artery exits through the foramina located near the base of the ascending processes of the parasphenoid [22, 23, 30]. Foramina or notches for internal carotid branches are present in the parasphenoid of †*Boreosomus* [58], most Mesozoic holosteans and stem teleosts [63].

The paired vomers and associated toothplates of NHMD_157546_A, and other †saurichthyids [29, 30] seem to reflect the primitive actinopterygian condition, as seen in anatomically generalized Paleozoic (e.g., †*Mimipiscis* and †*Moythomasia*) [44] and Mesozoic (e.g., †*Pteronisculus* and †*Australosomus*) [58, 59] taxa. This paired vomerine architecture is also encountered in extant holosteans [7, 9]. The presence of a median vomer and associated toothplate in the adult has evolved independently in several clades, such as Cladistia [79], inclusive of †scanilepiforms [12], some stem neopterygians (e.g., †*Luganoia*) [101], and teleosts [102]. A single vomer, with a toothplate that bears larger teeth along its midline, has been observed in the †saurichthyiform †*Yelangichthys* [32]. Given the broader distribution of this feature, we consider this condition as an apomorphy of †*Yelangichthys*. Acipenseriforms possess a series of paired or median, vomer-like elements, which may vary in number and which lie immediately anterior to the parasphenoid [6, 74]. The homology of these elements is yet unclear, though the posterior-most ossifications have been considered as vomers [6, 74].

#### Palatoquadrate and associated dermal ossifications of the cheek

**Fig. 10:**
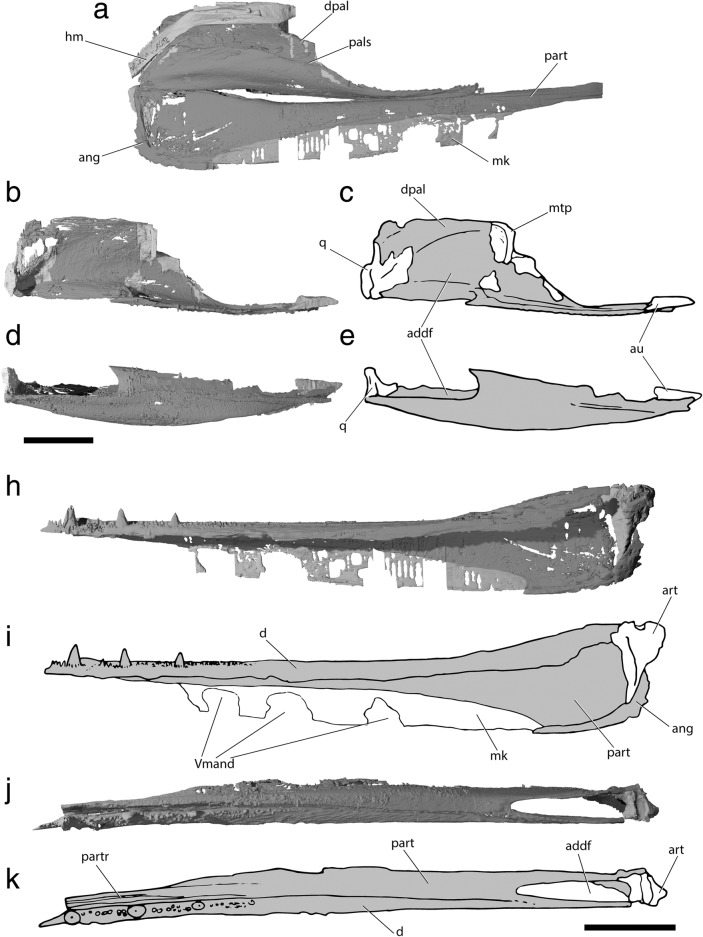
Palatal and lower jaw anatomy of †*Saurichthys* sp. (NHMD_157546_A); **a** digital rendering of left palate, lower jaw and hyomandibula in life association, medial view; **b** digital rendering of right palate in lateral view; **c** interpretative drawing of **b**; **d** digital rendering of right palate in ventral view; **e** interpretative drawing of **d**; **h** digital rendering of left mandible in lateral view; **i** interpretative drawing of **h**; **j** digital rendering of left mandible in dorsal view; **k** interpretative drawing of **j**. Gray shades indicate elements of dermal origin, white shades indicate endochondral origin. Abbreviations: **Vmand,** openings for mandibular trunk of trigeminal; **addf,** adductor fossa; **ang,** angular; **au,** autopalatine; **d,** dentary; **dpal,** dermal palate; **hm,** hyomandibula; **mk,** meckelian cartilage; **mpt,** metapterygoid; **pals,** palatal shelf; **part,** prearticular; **partr,** prearticular ridge; **q,** quadrate. Scale bars equal 1 cm

The palate of †*Saurichthys* (Fig. 10a–e) consists of rigidly connected dermal and endochondral ossifications that hosted the enlarged mandibular adductor muscle. The palatoquadrate is endochondrally ossified in at least two, and potentially three, parts. The quadrate (‘q’) forms the posteroventral margin of the endochondral palate, and the metapterygoid (‘mtp’) forms the dorsal margin. These two elements were previously described in †*Saurichthys* as being fused into a quadratometapterygoid [29], but no endochondral connection between the two was evident in the scan of NHMD_157546_A. An independent autopalatine (‘au’) forms the anterior margin of the endochondral palate. The quadrate forms the posterior margin of the adductor mandibulae fenestra (‘addf’) and bears two convex ventral condyles for articulation with the articular bone of the lower jaw. Medially, it exhibits a dorsoventrally oblique groove, where the ventral limb of the hyomandibula was accommodated. The metapterygoid is neither fenestrated, nor does it show any kind of anterodorsally expanded articular process. The only direct articulation between each palate and the neurocranium is seen anteriorly, where the independently ossified, triangular, pad-shaped autopalatine inserts to a similarly shaped fossa on the posterolateral floor of the ethmoidal region of the braincase.

Due to thinness, strong fusion among individual elements and breakage, the margins between individual bones of the dermal palate could not be reconstructed. The dermal palate (‘dpal’) forms a medially convex, cleaver-shaped apparatus. Laterally, it is rigidly connected to the maxilla. The posteroventral margin of the palate forms the anterior and medial surfaces of the large adductor foramen. The medial surface of the dermal palate, which likely corresponds to the area occupied by the entopterygoid [29], bears a prominent median shelf (‘pals’) along the length of its posterior half, which was likely associated with the palatine levator muscle, or with other ligaments connecting it to the parasphenoid. The anterodorsal margin of the bone is concave, without forming evident articular processes. The lingual surface of the dermal palate, anterior to the adductor fossa, bears sparsely arranged tiny teeth. Anteriorly, the part corresponding to the dermopalatine [29] forms a ventromedial crest that bears better defined, tiny teeth and occludes with the dorsomedial surface of the prearticular crest.

**Fig. 11:**
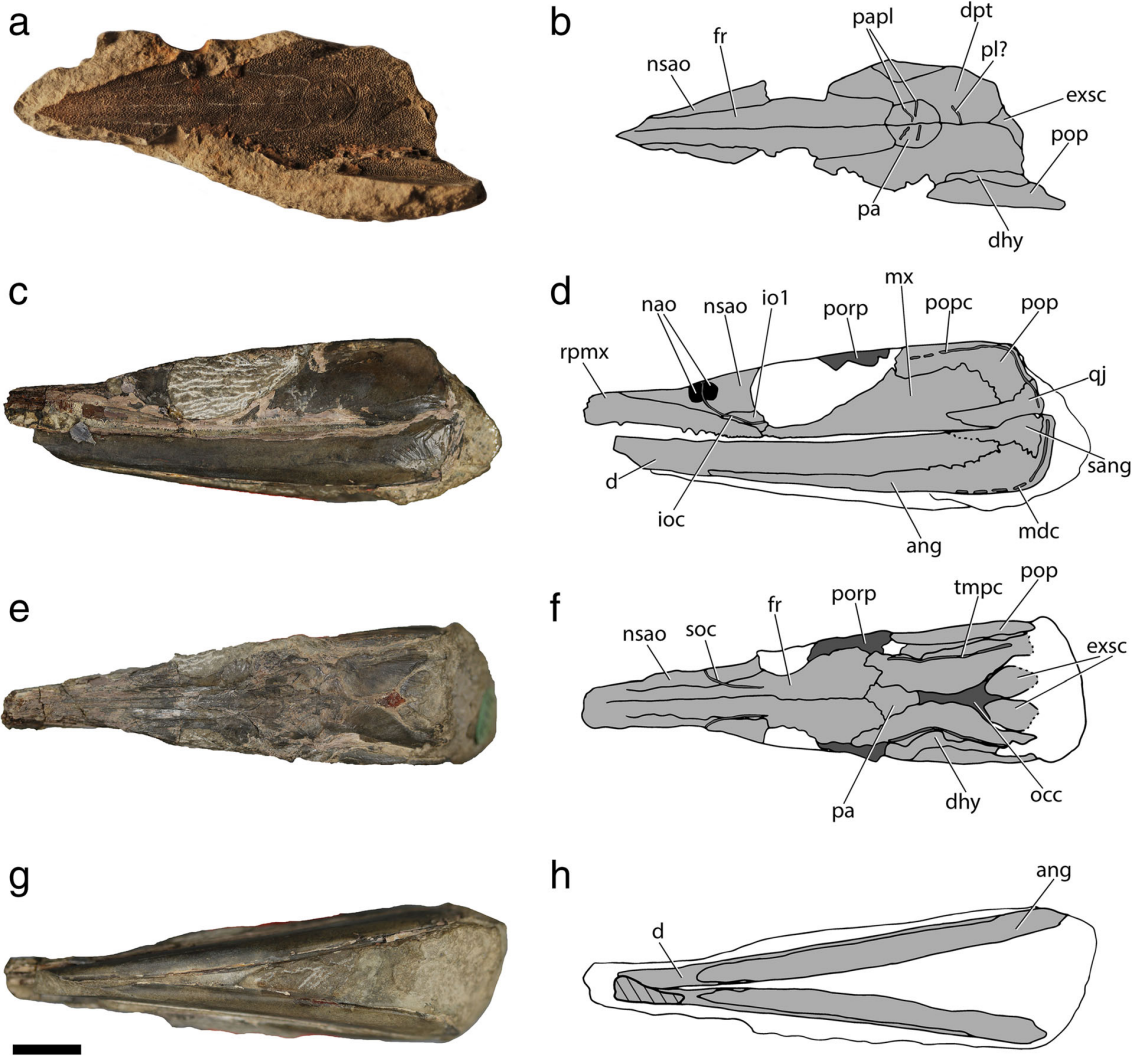
External anatomy of †*Saurichthys* sp. (NHMD_157546_A). **a** partial counterpart showing dorsal dermatocranium; **b** interpretative drawing of **a**; **c** skull and mandible in lateral view; **d** interpretative drawing of **c**; **e** skull and mandible in dorsal view; **f** interpretative drawing of **e**; **g** skull and mandible in ventral view; **h** interpretative drawing of **g**. Light gray shade indicates elements of dermal origin, dark gray shade indicates exposed regions of the chondrocranium. Abbreviations: **ang,** angular; **d,** dentary; **dhy,** dermohyal; **dpt,** dermopterotic; **exsc,** median extrascapular; **fr,** frontal; **ioc,** infraorbital canal; **la,** lachrymal; **mdc,** mandibular canal; **mx,** maxilla; **nao,** narial opening; **nsao,** nasalo-antorbital; **occ,** occipital crest; **pa,** parietal; **papl,** parietal pit line; **pl?,** putative pit line on dermopterotic; **pop,** preopercle; **popc,** preopercular canal; **porp,** postorbital process; **qj,** quadratojugal; **rpmx,** rostropremaxilla; **sang,** surangular; **soc,** supraorbital canal; **tmpc,** temporal canal. Scale bar equals 1 cm

The maxilla is cleaver-shaped (Fig. 11), forming an expanded posterior plate to which the arcuate preopercle (posterodorsally) and the quadratojugal (posteroventrally) suture to form a rigid unit. As with most other superficial dermal elements, the dermal bones of the cheek are poorly preserved in the specimen. The preoperculum is boomerang shaped, forming two distinct limbs, a horizontal and a more robust vertical one, separated by a dorsovental constriction of the bone. The course of the preopercular canal could not be clearly traced. The dorsal and posterior surfaces of the bone meet almost at a right angle, forming a rounded posterodorsal corner.

##### Remarks

A single, endochondral palatal ossification persists throughout ontogeny in most non-neopterygian actinopterygians, such as †*Cheirolepis*, †*Moythomasia*, the Madagascan †*Pteronisculus*, †*Australosomus*, and seemingly some neopterygians [44, 59, 103, 104]. In other non-teleostean actinopterygians, the adult palatoquadrate consists of distinct bones (or cartilages) arising from different ossification centers and exhibiting several variations [104], none of which includes a separate autopalatine and quadratometapterygoid ossifications as postulated by Stensiö [29] for †*Saurichthys*. The palatoquadrate of NHMD_157546_A likely conformed to the tripartite ossification pattern seen in polypterids, acipenseriforms, †*Birgeria stensioei*, †*Watsonulus*, *Amia*, and many teleosts [46, 47, 58, 74, 98, 104].

A high posterior extension of the palate is the plesiomorphic condition seen in Devonian actinopterygians [44, 103], and retained in most generalized forms of the Paleozoic and the Mesozoic [58, 59], including †*Saurichthys*. In †*Saurichthys*, †*Fukangichthys*, †*Birgeria*, and †*Woodichthys*, the dorsal part of the palatoquadrate forms no evident processes for articulation with the neurocranium [12, 59, 97]. In Devonian actinopterygians, the metapterygoid bears a circular opening for articulation with the basipterygoid process of the neurocranium [44, 103], whereas, in stratigraphically younger forms, the metapterygoid forms a notch (e.g., †*Australosomus*, [59]) or two processes (†*Pteronisculus*, *Amia*, [58, 104]) for articulation with the neurocranium and/or the attachment of ligaments connecting to the parasphenoid. As in other non-neopterygian actinopterygians [44, 58, 59, 79], the maxilla of †*Saurichthys* is non-kinetic. The shape of the preopercle of NHMD_157546_A is similar to the preopercle of †*Saurichthys* cf. *elongatus* from the Early Triassic (late Smithian) of Idaho [28] in exhibiting a dorsoventrally-wide horizontal limb. An anterior thickening of the preopercle is absent in †*Saurichthys ornatus*, or in any other species from Spitsbergen [29].

#### Dermal bones of the skull roof and rostrum

Our observations on NHMD_157546_A (Fig. 11) are in agreement with those of Mutter et al. [54], although further information on the skull roof is provided here, following our examination of the dorsal counterpart of the fossil. The superficial layers of the dermal bones, which bear ganoin ornamentation and the sensory canals, are missing from NHMD_157546_A, but are preserved in the counterpart. A single pair of elliptically shaped median extrascapulars (‘exsc’) is present on the posteromedial part of the skull roof, giving way anterolaterally to a pair of elongate dermopterotics (‘dpt’). The latter converge on the midline, but their anterior and posterior ends flare laterally. Part of the endochondral occipital crest (‘occ’) appears between the posterior part of the dermopterotics and the median extrascapulars, due to removal of the superficial layers of the bone during preparation. A field that contained the paired parietal bones (‘pa’) is present between the anterior part of the dermopterotics and the frontals (‘fr’). There is one semicircular parietal on each side of the midline. Each parietal seems to carry two pit lines (‘papl’), one extending posteromedially from the anterolateral part of the bone, and one extending medially from the lateral edge of the bone. The frontals are elongate and roughly triangular and taper rostrally, but their anterior tip is not preserved due to breakage in NHMD_157546_A. The frontals bear a lateral notch posteriorly for accommodating the supraorbital elements.

Anterior and lateral to the orbit, the frontals give way to the so-called nasalo-antorbitals (‘nsao’), which cover the posterolateral part of the rostrum, and encompass both narial openings (‘nao’) and the horizontal and ascending portions of the infraorbital sensory canal (‘ioc’), and its commissure with the supraorbital sensory canal. The ventrolateral margin of the preserved rostrum is occupied by the tooth-bearing rostropremaxilla (‘rpmx’). It is unclear whether the rostropremaxilla is paired or median, due to breakage. We did not find evidence for an ethmoidal sensory canal, but it is unclear if it was destroyed during preparation. The rostropremaxilla is ornamented with subvertical striae, as seen in some parts where the superficial ornamentation is preserved, and is also intimately connected with the maxilla (‘mx’). It comprises a dorsoventrally oriented lamina and a medially directed shelf, extending below the ethmoidal region and connecting medially with the vomers. The ventromedial part of the lamina bears two rows of sparsely arranged teeth. There is one marginal row of ventrally directed tiny conical teeth, and another row of ventromedially directed tiny teeth, the latter being interrupted at points by large laniaries. The median shelf of the rostropremaxilla forms a rostrocaudally directed groove for the accommodation of the largest teeth of the dentary. Large teeth also develop on that groove.

##### Remarks

One pair of (median) extrascapulars is present in most Early Triassic †saurichthyids and in the Middle Triassic †*Sinosaurichthys*, but an independent ossification is not apparent in other Middle Triassic or younger forms [23, 25, 29, 30, 40]. A second extrascapular pair is present in at least some specimens of †*Saurichthys madagascariensis* [25]. One or two pairs (median and lateral) of extrascapulars, typically carrying the supratemporal sensory commissure, are usually present in non-neopterygian actinopterygians [20, 44, 52, 58, 59, 105]. An increased number of extrascapular elements is seen, for example, in †*Chondrosteus* (one median, three paired; [11]). Living non-neopterygian actinopterygians display important inter- and intraspecific variations, with three to four paired extrascapular-like elements present on the skull of *Polypterus* [9, 80], one to two paired extrascapulars and a median extrascapular in *Acipenser* [74], and two paired extrascapulars in *Polyodon* [6].

In most actinopterygians, including most †saurichthyiforms, a single pair of parietals meets at the midline [17, 23, 25, 30, 32, 44, 52, 58]. Two pairs of parietals are present in †*Saurichthys ornatus* from Spitsbergen [29], suggesting that a different specific attribution of the Greenland specimen is warranted. A large number of tiny parietals is present in †*Saurichthys piveteaui* [39]. Additional variation in the number and shape of the parietals can also be seen in the sympatric †*Saurichthys* species from the Upper Buntsandstein (Anisian) [90].

Primitively, in Devonian and some Carboniferous actinopterygians, the supratemporal sensory canal is carried by two bones, the supratemporal posteriorly and the intertemporal anteriorly [44, 69, 96, 97, 106, 107]. In younger forms, like †*Saurichthys*, †*Birgeria*, †*Pteronisculus*, †*Australosomus*, †*Fukangichthys*, fossil neopterygians, and all extant actinopterygians, a single bone, the dermopterotic, occupies this position [6, 7, 9, 20, 29, 30, 58, 59, 74, 80, 98, 102]. One pair of frontals is present on the actinopterygian skull roof, bearing the supraorbital sensory canal and enclosing the pineal opening, when present [17, 44, 52, 58, 59, 69].

Uniquely among actinopterygians, †saurichthyiforms possess likely compound nasalo-antorbitals [23, 29, 30, 32], a term established on the fact that these bones carry both narial openings and the triradiate canal, formed by the horizontal, the ascending and the ethmoidal rami of the infraorbital sensory canal. The traditional terminology is retained herein. In most other non-neopterygian actinopterygians, the anterior nares are situated between the nasal and adjacent bones (either rostral or postrostral, e.g. in †*Mimipiscis*, †*Birgeria*, †*Pteronisculus*), and the posterior nares are located between the nasal and the antorbital (e.g. †*Birgeria*), or between the nasal and the orbital opening (e.g., †*Mimipiscis*, †*Boreosomus*, †*Pteronisculus*) [44, 58, 59]. The triradiate canal is primitively accommodated in the premaxilla [44], but in many late Paleozoic and younger taxa (e.g., †*Kalops*, †*Birgeria*, †*Teffichthys*, *Amia*) it is accommodated in an independent ossification, the antorbital [7, 59, 108, 109]. A commissure between the infraorbital and supraorbital canals accommodated between the nostrils occurs in some generalized genera like †*Kalops* and †*Boreosomus* [58, 108], in †saurichthyiforms [23, 29]; Fig. 11c, d), and stem (e.g., †*Teffichthys* [109]) and crown neopterygians [7, 9]. A commissure between the two sensory canals is absent in †*Birgeria* [59]. In Acipenseriformes, the infraorbital sensory canal does not form an ascending ramus [6, 74]. In *Polypterus*, the connection between the infraorbital and supraorbital canals takes place anterior to the single narial opening, through a likely compound element formed by the premaxilla and the rostral [80].

The prominent †saurichthyid rostrum is formed mainly by the rostropremaxilla(e), whose ontogenetic origin remains unknown. Due to the acuteness of the snout, it is unclear if these elements are paired [23, 29, 30] or unpaired [25]. The presence of an anterior ramus of the infraorbital sensory canal and teeth in the rostropremaxillae of most †saurichthyids [23, 29, 30], combined with their topology and posterior development, suggests that the premaxilla, and potentially the rostral, plays an integral part in the development of the rostrum. This also seems to also apply to †*Birgeria*, although in the latter taxon the rostropremaxilla additionally borders the anterior narial opening [59]. In primitive actinopterygians, the anterior-most rostral is often expanded ventrally, bears the ethmoidal commissure and teeth, and forms the anterodorsal tip of the oral rim, for instance in †*Moythomasia* [44]. The rostral is flanked by paired premaxillae, bearing the anterior and the ascending rami of the infraorbital sensory canal [44]. Loss, fusion or fragmentation of those elements is common in non-neopterygians (e.g., in †*Wendyichthys*, †*Cyranorhis*, †*Pteronisculus*, †*Australosomus* and in acipenseriforms, the premaxilla is probably absent, [40, 58, 59, 74, 106]), but a detailed discussion is beyond the scope of this work.

A pair of postrostral elements, situated between the frontals and the rostropremaxilla(e), is potentially present in Early Triassic †*Saurichthys* species from Spitsbergen [29].

A higher number of postrostrals were tentatively reconstructed for †*S. stensioi* and †*S. piveteaui* from Madagascar [39, 40]. Postrostrals are unknown in most other †saurichthyids [22, 23, 25, 30], although a single pair was tentatively reconstructed for †*Saurorhynchus acutus* [110].

#### Circumorbital bones and ossifications of the orbit

Most circumorbital bones of NHMD_157546_A have been pushed medially inside the orbits and are still covered by matrix (Figs. 1a, 2a). As a result, they were not previously described [54]. The dorsal margin of the orbit is formed by one or two supraorbitals (incompletely preserved and broken on both sides of the skull, ‘so’) and the dermosphenotic (‘dsph’). Mutter et al. [54] misidentified the externally exposed postorbital process of the braincase as the dermosphenotic. The dermosphenotic is anteroposteriorly elongate and laterally convex and bears a broad ventral articulation surface for the attachment of the jugal. The jugal (‘ju’), being anteriorly concave and posteriorly convex, forms the posterior margin of the orbit. It starts vertically below the dermosphenotic, but forms a gentle anterior curve and tapers towards its articulation with the second infraorbital (‘io2’). The latter is talon shaped and forms the posterior part of the ventral orbital margin. The infraorbital canal passes anteriorly to an elongate first infraorbital (Fig. 11: ‘io1’), wedged between the nasalo-antorbital and the anterior prossess of the maxilla. A single, well-developed, sub-triangular anamestic suborbital (‘subo’) bone covers the space between the jugal and the expanded posterodorsal process of the maxilla. All the above dermal bones are ornamented with tubercles that are sometimes connected to form short, vermiform ridges.

A thin sclerotic ring (Figs. 1a, b, 2a, b: ‘sclt’) is preserved in situ on both sides of the skull. The number of individual ossifications could not be confidently determined. The diameter of the sclerotic ring is only slightly smaller than that of the enlarged orbital space. The outer dorsal and ventral surfaces of the sclerotic ossicles are ornamented with randomly arranged turbercles, whereas the inner surface is smooth. Traces of the cartilaginous sclera (‘scla’) are also preserved, contained within the sclerotic rings and curving towards the midline of the skull.

##### Remarks

Amongst †saurichthyids, supraorbitals seem to be restricted to Early Triassic forms [25, 29, 39, 40] (Figs. 1a, 2a), and are unknown from stratigraphically younger species [22, 23, 27, 30]. Supraorbitals are primitively absent in actinopterygians [18, 40, 44, 58, 59, 96], but are also absent in *Polypterus* and *Amia* [7, 80]. One supraorbital is present in †*Discoserra*, *Acipenser*, †*Watsonulus*, and gars, but three or more are seen in forms like †*Kalops*, †scanilepids, stem neopterygians (†‘subholosteans’) such as †*Luganoia* and †*Peltopleurus*, and some stem teleosts [9, 12, 20, 74, 101, 102, 108, 111, 112]. The dermosphenotic of †*Saurichthys* resembles that of e.g., †*Pteronisculus,* †*Boreosomus* and *Acipenser* [58, 74] in lacking a posterior process. This contrasts with both the primitive actinopterygian condition and that seen in e.g., †*Birgeria groenlandica* and extant forms like *Polyodon*, where the dermosphenotic forms a posterior process [6, 59].

NHMD_157546_A resembles the Early Triassic †saurichthyids from Spitsbergen [29] and Madagascar [39] in exhibiting three infraorbitals. This seems to be the primitive condition in the group. All Devonian and most Carboniferous actinopterygians exhibit two infraorbitals: a jugal (forming the posteroventral margin of the orbit) and a first infraorbital (or lachrymal, forming the anteroventral margin of the orbit) [44, 96, 105]. Additional infraorbitals, often more than one, are seen in many stratigraphically younger forms like †*Boreosomus* [58] and †*Birgeria* [59]. At least two infraorbitals are present in *Acipenser* [74], whereas numerous small, canal-bearing ossicles are seen in *Polyodon* [6]. Only a single infraorbital bone is present in *Polypterus*, with the infraorbital canal largely borne by the maxilla [80]. †Scanilepids have two infraorbitals [19, 20].

The numbers of suborbital bones vary greatly in post-Devonian actinopterygians, with Early Triassic †saurichthyids having one [29] (Fig. 1a, 2a), †*Pteronisculus* having two or more, †*Boreosomus* having five [58], and †*Birgeria* having more than 10 [59]*.* No suborbitals are known in post-Early Triassic †saurichthyids [22, 23, 27, 30]. Suborbitals are absent in extant Acipenseriformes [6, 74]. A series of small anamestic bones homologous to suborbitals, but referred to as ‘spiraculars’, separate the cheek from the orbit and the dorsal skull roof in extant polypteriforms [80]; three of these elements are typically present in †scanilepids [12]. In *Amia* the suborbitals are also absent, whereas in Lepisosteiformes they are greatly reduced in size and multiplied to form a mosaic on the lateral surface of the cheek [7, 9]. Numerous suborbitals are present in stem teleosts, but are absent in extant taxa [102].

#### Lower jaw

The lower jaws are almost straight (Figs. 10h–k, 11). Three dermal bones are seen on the lateral surface of each mandible. The posterolateral corner is occupied by the elongate, triangular surangular (‘sang’). The angular (‘ang’), on the posteroventral corner of the jaw, is more elongate and reaches the level of the external nares anteriorly. Though damaged during preparation, a faint groove along its ventral margin indicates the course of the mandibular sensory canal (‘mdc’). Posteriorly, it wraps around the posterior surface of the articular and reaches the posteromedial surface of the lower jaw. The dentary (‘d’) is the largest and the main dentigerous bone of the lower jaw. It begins posteriorly between the angular and the supraangular, and in †saurichthyids it usually extends to the symphysis. Only its dorsal part is visible in our tomograms. The dentary curves medially to form a medial dermal lamina, which supports an elongate dental lamina along its preserved length. The tooth plate is occupied by patches of tiny teeth, starting from below the otic region of the neurocranium and becoming more numerous and better developed anteriorly. Starting from the level below the nostril and extending to the tip of the preserved part of the jaw, a single file of coarsely-spaced, caniniform teeth interrupt the continuity of smaller tooth patches. Although few caniniform teeth are actually preserved in our specimen, we can deduce that they occur in alternate positions between the two jaws, forming a dental basket. The base of the caniniform teeth is made of crenelated dentine (plicidentine), while the apex is formed by an acrodin cap, equal or slightly shorter than a fifth of the tooth height. The pulp cavity is wide and terminates slightly above the mid-height of the tooth, but does not reach into the acrodin cap (Additional file 3: Figure S2B, C). Caniniform teeth in the upper jaw seem to share the same structure.

The large prearticular (‘part’) covers most of the dorsomedial aspect of the lower jaw posteriorly, and tapers anteriorly. A dorsolateral projection of the bone articulates between the medial dermal lamina of the dentary and the overlying dental lamina. Miniscule teeth appear at the same level as the teeth of the dentary. More anteriorly, below the mid-length of the orbit, the prearticular forms a dorsomedial crest, which becomes more prominent at the level of appearance of the caniniform teeth of the dentary. This crest is largely edentulous and occluded with the vomers.

The endochondral articular (‘art’) is triangular in shape and bears a dorsal glenoid fossa with two pits for the articulation of the condyles of the quadrate. Anteroventrally, the articular passes to the very thin and weakly ossified meckelian cartilage (‘mk’). It is unclear if the two elements were connected. The meckelian cartilage is ventral to the prearticular and partially covered by the latter bone, taking the form of an internal lining. A series of wide, circular ventral openings is present and can be associated with the innervation from the trigeminal nerve (‘Vmand’). A large fenestra for the mandibular adductor muscle is present on the posterodorsal corner of the bone, immediately anterior to the articular, and is bounded by the articular, the dentary and the prearticular bones.

##### Remarks

A surangular in the lower jaw seems primitively present in Devonian actinopterygians [69, 105, 113], and is common in Permian–Triassic taxa such as †*Saurichthys*, †*Pteronisculus*, †*Australosomus*, †*Birgeria*, †*Fukangichthys*, and early crown neopterygians, like †*Watsonulus* and †‘pholidophorids’ [12, 29, 58, 59, 98, 102]. Loss of the surangular has occurred multiple times in non-neopterygians, like e.g., †*Mimipiscis*, †*Gogosardina*, †*Amphicentrum*, †*Aesopichthys*, acipenseriforms and *Polypterus* [44, 64, 80, 114, 115]. †*Saurichthys,* like most fossil non-neopterygian actinopterygians, lacks a coronoid process in the lower jaw for the attachment of the mandibular adductor muscle [44, 58, 59]. By contrast, cladistians (inclusive of †*Fukangichthys*), †*Birgeria* and neopterygians bear a dermal coronoid process. The components of this structure vary between groups, suggesting multiple independent origins [12, 59, 80, 98]. The lower jaw dentitions of Early Triassic †saurichthyids have neither been described nor adequately illustrated [29], hampering further comparison with the Greenland specimen. Plicidentine has arisen multiple times in modern lineages of hyper-piscivorous actinopterygians, but is also present in †*Cheirolepis* [116]. The expanding list of taxa exhibiting plicidentine, which now includes †*Saurichthys*, suggests that the distribution of this feature is controlled by function, rather than phylogeny.

#### Operculogular series

The opercular series is largely not preserved in NHMD_157546_A. Only a single branchiostegal ray is preserved in this specimen (Fig. 1c, 2c: ‘rbr’), underlying the posterior part of the ceratohyal. The branchiostegal is lozenge shaped, with rounded anterior and posterior ends. Its ventral face is ornamented with well-developed tubercles, but bears an unornamented field along its posteromedial margin. On the opposite (right) side of the branchiostegal, and anterior to it, there is a flat, splint-like dermal element, underlying the anterior part of the right ceratohyal and extending anteriorly slightly past its rostral end. Its ventral face is also ornamented with tubercles. There is no sign of a lateral field for the insertion/overlap of the branchiostegal element, allowing us to identify the splint-like element as a lateral gular (‘latg’).

##### Remarks

One pair of branchiostegals is known in Early Triassic [25, 29] and Middle Triassic †saurichthyids [23, 25, 29]. A second pair has been identified in the Middle Triassic †*Saurichthys yangjuanensis* [36].

The number of branchiostegal rays varies among Paleozoic ctinopterygians, being usually higher than 10 [44].

The single pair of splint-shaped gulars of the Greenland †*Saurichthys* seems to correspond to the primitive condition in the clade. Gulars were previously thought to be absent in †saurichthyids. Given the large sample sizes investigated, they are likely lost in Middle Triassic and younger forms (e.g., [22–24, 27, 30, 36]. Most non-teleostean actinopterygians exhibit gulars, with the primitive pattern corresponding to the presence of one median gular and a pair of lateral gulars, like in †*Cheirolepis*, †*Mimipiscis*, †*Raynerius*, †*Pteronisculus,* †*Birgeria*, †*Watsonulus* and some Triassic ‘†pholidophorids’ [44, 58, 59, 69, 96, 98, 102, 103]. Acipenseriforms, †*Chondrosteus*, ginglymodians and most crown teleosts have no gulars [6, 9–11, 74].

Despite the limitations of the material described here, a comment on the phylogenetically and functionally important opercular bones of †saurichthyids is warranted. The largest bone of the †saurichthyid opercular series is historically identified and treated as an operculum [22, 23, 25, 27, 29, 30, 117]. Nevertheless, Stensiö also considered the possibility of a more complex evolutionary history for this bone through fusion of separate elements [29]. In most actinopterygians, the opercle forms an anteromedial process and fossa, which articulates with the opercular process of the hyomandibula [7, 9, 58, 59, 79]. In primitive forms like †*Cheirolepis*, †*Mimipiscis*, †*Moythomasia* and †*Raynerius*, the opercle articulates directly with the posterior face of the ‘knee’ of the hyomandibula [44, 68, 69, 103]. In fossil chondrosteans with a reduced opercle, like †*Chondrosteus*, †*Peipiaosteus* and †*Stichopterus*, the latter bone is not in contact with the hyomandibula, but sits on the dorsal side of an enlarged subopercle [11, 118].

**Fig. 12:**
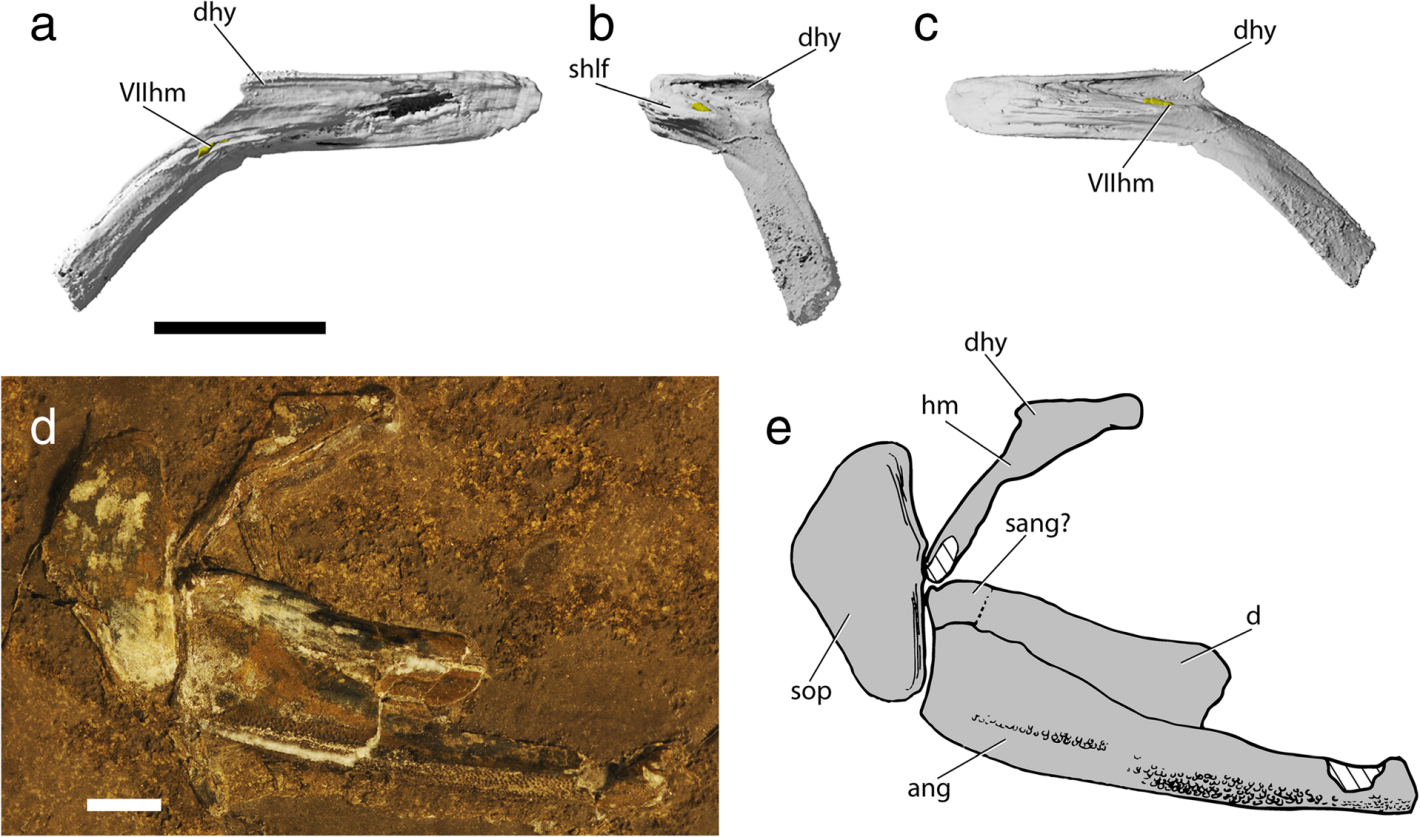
Hyomandibular and opercular anatomy of †saurichthyids. Digital rendering of right hyomandibula of †*Saurichthys* sp. (NHMD_157546_A) in: **a** lateral view; **b** posteromedial view; **c** medial view; **d** right hyomandibula, subopercle and mandible of an unidentified †saurichthyid (PIMUZ A/I 4648) from Prosanto Formation (Ladinian, Switzerland) in life position; **e** interpretative drawing of **d**. Gray shade indicates elements of dermal origin. Abbreviations: **VIIhm,** hyomandibular trunk of facial nerve; **d,** dentary; **dhy,** dermohyal; **hm,** hyomandibula; **sang?,** putative surangular; **sop,** subopercle. Scale bar equals 1 cm

Additional †*Saurichthys* material from the Middle Triassic of Switzerland (Fig. 12d, e), as well as a review of figured specimens (e.g. [29]: pls. 11, 14, 22, 27, 28) reveals that the articulation between the so-called ‘operculum’ and the hyomandibula occurs much more ventrally than previously thought, at the ventral tip of the latter bone. This mode and topology of articulation implies that the ‘opercle’ is actually an expanded subopercle (‘sop’), and is broadly comparable to that of Chondrostei, where the expanded subopercle articulates with the posteroventral cartilaginous head of the hyomandibula [6, 11, 74]. However, †*Saurichthys* is the only known actinopterygian whose subopercle forms an anteromedial articular process and fossa for articulation with the hyomandibula [23, 29]. In other actinopterygians, the subopercle articulates with the posterior surface of the preopercle and the ventral surface of the opercle, and is ligamentously attached to the ventral limb of the hyomandibula [7, 70, 79].

This inference gains additional support with the identification of an additional opercular element in †*Saurichthys ornatus* and †*S. hamiltoni* from the Early Triassic of Spitsbergen (unlabeled in [29]: pls. 11, 27, 28) and †*Saurichthys madagascariensis* (termed as antoperculum in [39]: Fig. 10; [25]: Fig. 6). This small dermal bone wedged between the preopercle, the dermohyal (present although not labelled) and the expanded subopercle is situated at the level of the back of the ‘knee’ of the hyomandibula, and is topologically equivalent and likely homologous to the opercle. An expansion of the subopercle at the expense of the opercle has occurred several times in Actinopterygii, with early chondrosteans [11, 118], †*Canobius* [119], †*Styracopterus* [120], and †*Teffichthys* [109] being some examples of seemingly independent acquisition of this character.

#### Hyoid and branchial arches

The slender, boomerang-shaped hyomandibula (Figs. 10a, 12a–c: ‘hm’) has a well-defined, horizontal anterodorsal limb and a posteroventral limb. The dorsal surface of the dorsal limb is flat and wide, potentially serving as the insertion point of the retractor muscle. A dermohyal (‘dhy’) is firmly fused on the dorsolateral to lateral surface of the anterodorsal limb of the hyomandibula. No ornamentation of the dermohyal is apparent in the scan. However, the compactness of the dermohyal ossification contrasts sharply with the cancellous endochondral nature of the main body of the hyomandibula, testifying to its dermal origin. The dermohyal expands dorsally, forming a lateral wall with a T-shaped cross section on the hyomandibula. The dorsal surface of the dermohyal was accommodated between the preopercle and the dermopterotic in life. The posterodorsal tip of the dermohyal stands out from the body of the hyomandibula, forming an angular projection. This projection was previously erroneously identified as an opercular process in †*Saurichthys curionii* [23]. An opercular process is absent from the hyomandibula of NHMD_157546_A. A canal for the hyomandibular trunk of the facial nerve (‘VIIhm’) starts at the posteromedial part of the dorsal limb and exits laterally to the ‘knee’ of the bone. Additional ossifications intercalated between the hyomandibula and the ceratohyal (e.g., interhyal, symplectic) were not observed.

**Fig. 13:**
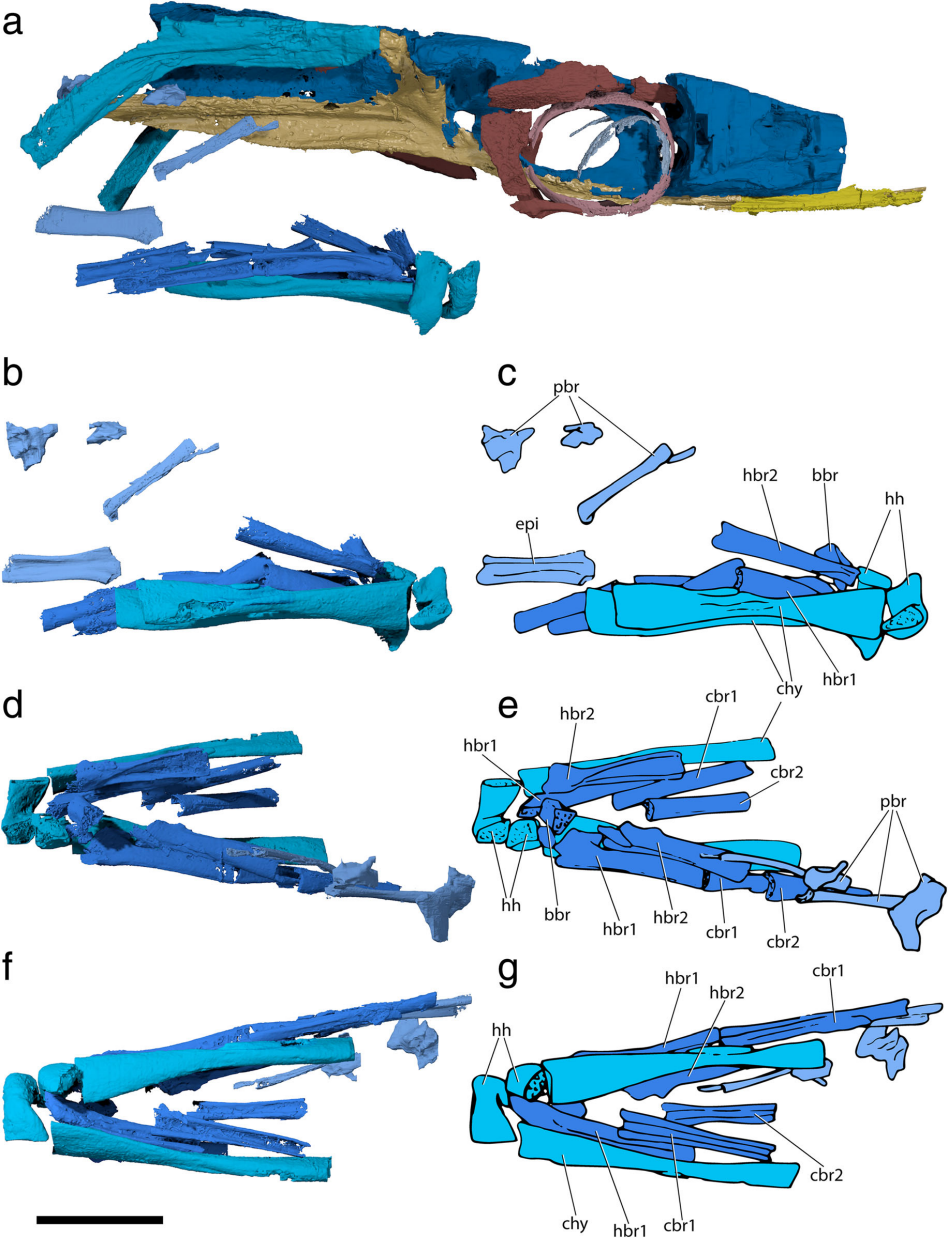
Hyoid and gill skeleton of †*Saurichthys* sp. (NHMD_157546_A). **a** Digital rendering of braincase and associated hyoid and branchial ossifications in left lateral view (mirrored); **b** ventral hyoid and gill ossifications in right lateral view; **c** interpretative drawing of **b**; **d** ventral hyoid and gill ossifications in dorsal view; **e** interpretative drawing of **d**; **f** ventral hyoid and gill ossifications in ventral view; **g** interpretative drawing of **f**. Abbreviations: **bbr,** basibranchial; **cbr1,** ceratobranchial 1; **cbr2,** ceratobranchial 2; **chy,** ceratohyal; **epi,** epibranchial; **hbr1,** hypobranchial 1; **hbr2,** hypobranchial 2; **hh,** hypobranchial; **pbr,** pharyngobranchial. Scale bar equals 1 cm

A single ceratohyal (Fig. 13: ‘chy’) is present on either side of NHMD_157546_A. The ceratohyal is slender, slightly twisted around its long axis and of elongate hourglass shape. The lateral surface of the bone bears a shallow groove for the afferent hyoidean artery. The hypohyals (‘hh’) are slightly dislocated from their natural position. They are strongly curved medially, and they likely articulated with the first basibranchial element. Their median part is thicker than their posterior part, the latter forming an elliptical head for articulation with the ceratohyal. No basihyal was observed.

The branchial skeleton of †*Saurichthys* is only partially preserved and largely disarticulated (Fig. 13). A rod-like and grooveless endochondral structure on the left side likely corresponds to the first infrapharyngobranial. Posterior to the rod like bone there are two dorsoventrally short and robust pharyngobranchials (‘pbr’). They form a medial shelf for the passage of the efferent branchial arteries. Immediately ventral to the posterior tip of the rod-like bone, there is a dislocated epibranchial (‘epi’), which was likely the first of the series. Its dorsal tip bears two surfaces for articulation of the pharyngobranchials, but no uncinate processes. The epibranchial bears a lateral groove for the corresponding efferent artery. The ventral elements of the first two branchial arches are preserved. The ceratobranchials (‘cbr’) are straight, exhibiting a conspicuous ventral groove for their corresponding efferent arteries. The hypobranchials (‘hbr’) are imperforate, straight for the most part and deeply grooved ventrally for the passage of the efferent arteries. The grooves disappear slightly before the anterior articular head of the bones. The first hypobranchials are hatchet shaped, with their anterior tip forming a broad, median expansion for articulation with the corresponding basibranchial element. They lack facets for articulation with the hypohyals. The mesial head of the second hypobranchial is narrower. Nothing remains of the more posterior arches. One basibranchial (out of the expected three [29]) is preserved. It has a subtriangular cross-section, a flat dorsal surface and weak ventral keel. No articulation surfaces for the hypobranchials were identified on the basibranchial.

##### Remarks

†*Saurichthys* shares a similar hyomandibular morphology (boomerang shaped; single head for articulation with the braincase; lack of opercular process; canal for the facial nerve (VII); fused dermohyal) with Devonian actinopterygians like †*Mimipiscis*, †*Moythomasia*, †*Howqualepis* and †*Raynerius* [44, 69, 96]. In †*Saurichthys*, the dermohyal occupied a more dorsal position, being wedged between the preoperculum and the dermopterotic, rather than between the preoperculum, the dermopterotic (or homologues), and the operculum as in other early actinopterygians. It is possible that this is due to the hypothesized changes to the opercular series outlined above, and/or the elongation of the posterior portion of the †saurichthyid skull. The hyomandibula of chondrosteans lacks both an opercular process, and a dermohyal [6, 11, 74]. *Polypterus*, †*Fukangichthys*, †*Pteronisculus*, †*Boroesomus*, †*Australosomus* and neopterygians bear a distinct opercular process [7, 9, 12, 58, 59, 79, 98]. The dermohyal is not fused to the hyomandibula in other post-Devonian actinopterygians, including *Polypterus* [18, 47, 58, 59, 66, 79]. It is generally absent in crown neopterygians [7, 98], although it is present in crownward members of the stem lineage like †*Luganoia* and †*Peltopleurus* [101], and likely also in gars [9]. The presence of a facial nerve canal on the hyomandibula is widespread in Actinopterygii (e.g., [44, 98]), but is absent in polypterids, †*Fukangichthys*, acipenseriforms, †*Cheirolepis*, and †*Boreosomus* [6, 12, 58, 68, 74, 79].

In Devonian actinopterygians, †*Fukangichthys* and *Polypterus*, the ceratohyal consists of a single ossification [12, 44, 79], but in †*Pteronisculus* and neopterygians there is a smaller posterior ceratohyal ossification [7, 9, 58, 98]. A groove for the afferent hyoidean artery is a plesiomorphic osteichthyan feature retained in many fossil actinopterygians like †*Raynerius*, †*Mimipiscis*, †*Moythomasia*, †*Pteronisculus*, †*Australosomus*, and †*Fukangichthys* [44, 58, 59, 69]. It is absent in *Polypterus*, *Polyodon* and †*Chondrosteus* [11, 74, 79]. However, a shallow depression is seen in the posterolateral half of the ceratohyal of *Acipenser* (TA pers. obs on *Acipenser*, UMMP unnumbered teaching collection specimen). †*Watsonulus* [98] also shows a groove, but this feature is absent in living holosteans and teleosts [7, 9].

Current knowledge about the fossil record of actinopterygian gill skeletons is limited, largely because such structures are rarely preserved, and, where present, are difficult to access without recourse to destructive methods (but see [121]). The overall anatomy of the †*Saurichthys* gill skeleton does not appear to differ significantly from that of generalized Permian–Triassic actinopterygians like †*Pteronisculus* [58]. A ventral gill skeleton of a †saurichthyid from Spitsbergen, figured by Stensiö ([29]: Pl. 7), preserves four ceratobranchials. Stensiö’s reconstruction ([29], fig. 26), however, depicts five ceratobranchials, but no further evidence was provided. Five ceratobranchials are primitively present in actinopterygians, with the fifth being usually less well-developed [44, 58, 69]. Cladistians have only four gill arches, missing the fifth arch completely [122], which is likely an apomorphic feature of the clade, inclusive of †*Fukangichthys* [12].

The morphology of most branchial elements is slightly modified in †*Saurichthys*, becoming more elongate, straight and more slender, to follow the pattern of cranial elongation seen in the clade. In †*Pteronisculus*, there is an expanded infrapharyngobranchial, suspending the third and fourth branchial arches [58]. In †*Mimipiscis*, the hypobranchials are proximally perforated [44], but this feature was not observed in other actinopterygians like †*Raynerius* [69] or †*Saurichthys*. The hypobranchials of †*Saurichthys* form a single, median articulation with the corresponding basibranchial elements and show no ventromedial processes, like those present in the second and third hypobranchials of *Amia* and other neopterygians [7, 71] (TA pers. obs. on *Amia calva*, UMMP unnumbered teaching collection specimen).

The ventral branchial skeleton of †*Saurichthys ornatus* from Spitsbergen exhibits three distinct basibranchial ossifications [29]. Only a single basibranchial is preserved in the Greenland †*Saurichthys*, but is dorsally displaced, anteroposteriorly short and bears no lateral ossification surfaces for the hypobranchials, differing from the massive, single basibranchial copula of Devonian actinopterygians [44, 69], and *Polypterus* [79]. †Saurichthyids seem to bear three basibranchial ossifications [29] like †*Pteronisculus* [58]. At least two basibranchials are present in ‘†*Elonichthys*’ [123], and two basibranchials were described in †*Fukangichthys* [12]. †*Australosomus* exhibits four basibranchial ossifications, with the posterior-most basibranchial being longitudinally pierced by a paired canal for the fourth afferent branchial arteries [59]. Living chondrosteans have no ossifications in their ventral gill skeleton. Instead, there is an enlarged, cartilaginous anterior basibranchial that articulates with hypobranchials 1–3, and a posterior cartilaginous basibranchial that articulates with the fourth hypobranchials [6, 74]. However, there is considerable variation within sturgeons, and one or two additional basibranchial cartilages might be present in some individuals [74]. In *Amia,* only the posterior part of the anterior basibranchial ossifies, while the two posterior basibranchials remain cartilaginous [7]. Two basibranchials are present in *Lepisosteus*, with only the anterior part of the second basibranchial known to ossify [9]. The basibranchial series of teleosteans comprise between three and five distinct ossifications [124].

#### Dermal bones of the pectoral girdle

Only two elements of the pectoral girdle are preserved in the Greenland †*Saurichthys*, both disarticulated from their adjacent bones. Posterodorsally, there is an angled, anamestic dermal element (Fig. 1c, e, 2c, e: ‘pt-sc’). This enigmatic bone forms an unornamented anteriorly–anteromedially expanding process and a lateroventrally–ventrally expanding lamina, which bears tubercles. A clavicle (‘clav’) is preserved ventrally, and has been displaced to punch through the gill skeleton. It is thin, with an elongate triangular shape, pointing anteriorly, and is strongly convex laterally. Its mesial surface is slightly thickened and was likely abutting its antimere in life.

##### Remarks

In Early Triassic †saurichthyids and in †*Yelangichthys*, there are two canal-bearing, dermal bones, the posttemporal and the supracleithrum, connecting the cleithrum with the skull [25, 29, 32]. The arched bone in NHMD_157546_A resembles the compound posttemporal-supracleithrum of Middle Triassic †saurichthyids [23, 30], however the latter bone is always canal bearing. The absence of a canal in NHMD_157546_A could either be a peculiarity of the specimen/species, or could imply that a presupracleithrum is present. The latter ossification is absent or unknown in most †saurichthyids, but has been tentatively reconstructed in the Anisian species †*Sinosaurichthys longimedialis* [30]. Well-developed triangular clavicles are typically present in all non-neopterygian actinopterygians (e.g., [6, 44, 58, 59, 74, 80]), and also in some early neopterygians such as †*Watsonulus* [98]. Clavicles become much reduced or lost in holosteans and early teleosteans [7, 9, 102, 125].

### Systematic paleontology

†*Saurichthys nepalensis* Beltan and Janvier, 1978 [126].

#### Material

MNHN F 1980–5, †*Saurichthys nepalensis*, partial skull.

#### Fossil age and locality information

Fossil fishes from the Early Triassic of the Himalayas are rare and poorly known [14, 126, 127]. The Early Triassic deposits of the Annapurna, Nepal, have only produced a single actinopterygian fossil (MNHN F 1980–5): the holotype of †*Saurichthys nepalensis* [126]*.* The skull was found in the Thini Gaon area, but was lying amongst debris and the precise geological horizon remains unknown. The surrounding matrix was tentatively correlated, on the basis of lithological similarities, with lowest Triassic (‘lower Scythian’; ~ 251 Ma) ammonoid-bearing facies that occur in the area [126]. Additional details of Triassic stratigraphy of the Annapurna, including Thini Gaon, are given by Garzanti et al. [128]. The holotype of †*S. nepalensis* corresponds to a fragmented skull, preserving only the anterior orbital region and the posterior rostroethmoidal region. During preparation for the initial description, the skull was immersed in 5% formic acid [126]. Although this procedure damaged the specimen, the almost complete removal of the matrix resulted in excellent contrast using μCT.

### Anatomical description

#### Ethmoidal region

**Fig. 14:**
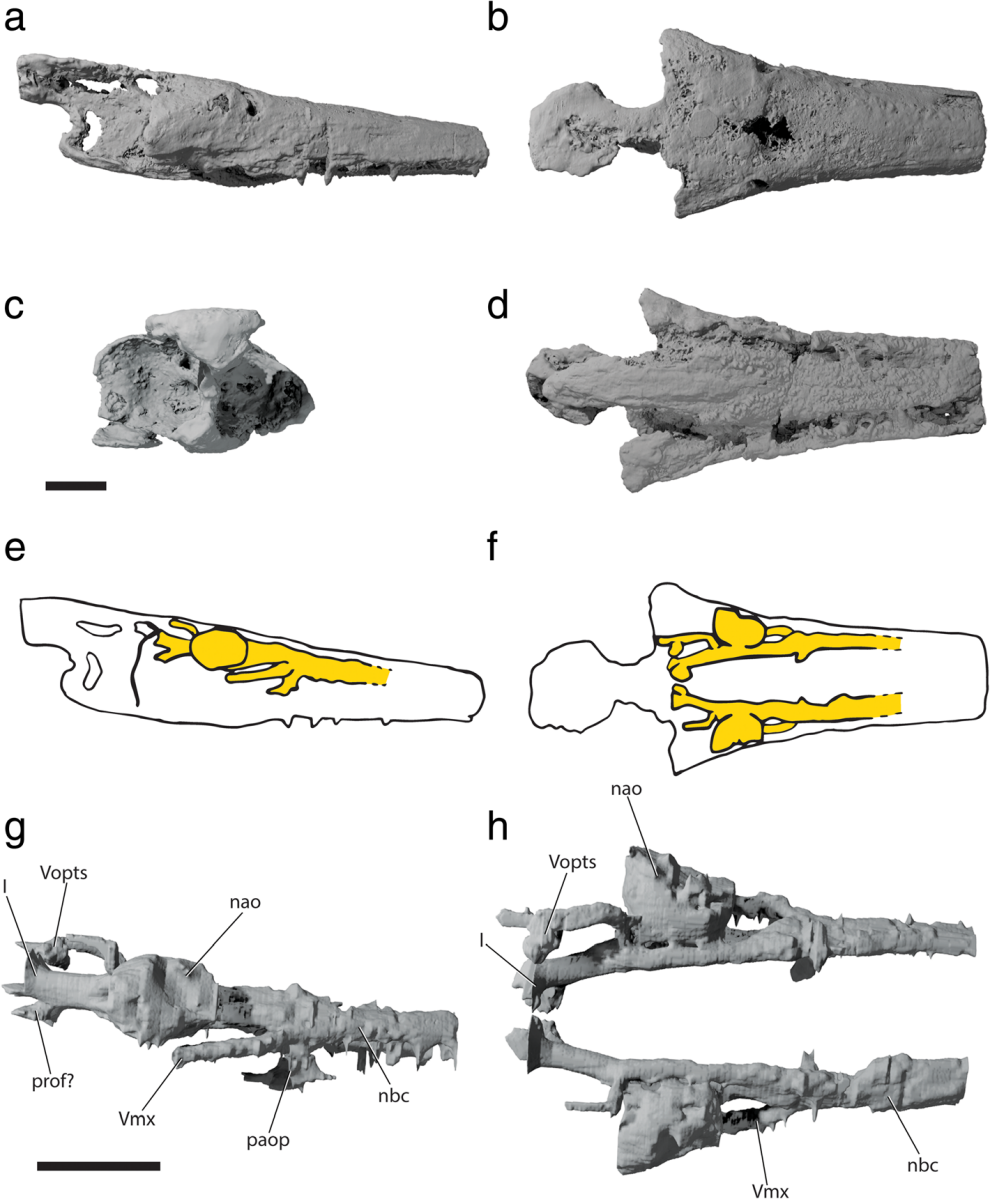
Ethmoid region of †*Saurichthys nepalensis* (MNHN F 1980–5). Digital rendering of complete specimen in: **a** right lateral view; **b** dorsal view; **c** posterior view; **d** ventral view; schematic exhibiting the arrangement of rostral canals (in yellow) in: **e** lateral view; **f** dorsal view; digital rendering of rostral canals in: **g** lateral view; **h** dorsal view. Abbreviations: **I,** olfactory nerve; **Vmx,** maxillary ramus of trigeminal nerve; **Vopts,** superficial ophthalmic ramus of trigeminal nerve; **nao,** narial opening; **nbc,** nasobasal canal; **paop,** palatal opening of nasobasal canals; **prof?,** putative course of profundus nerve. Scale bars equal 1 cm

The ethmoidal region of †*Saurichthys nepalensis* (MNHN F 1980–5; Fig. 14) differs in some respects from that of the Greenland †*Saurichthys* (NHMD_157546_A). More specifically, in †*S. nepalensis*, the interorbital septum is not ossified along the course of the olfactory tracts, although this may well be an artefact of preservation or preparation. The interorbital fenestra is much smaller and kidney-shaped, rather than oval. The anteroventral myodome is paired and not median. The remainder of the ethmoidal region is otherwise very similar to that of NHMD_157546_A. In terms of internal anatomy (Fig. 14e–h), the olfactory nerve lobes (‘I’) diverge laterally towards the external nares, upon entering the ethmoidal region. They give off multiple branches that connect with the nasal cavities and openings (‘nao’). At the level of the posterior tip of the nasal cavity, each dorsal-most branch receives a canal of posterodorsal origin, which must have carried the superficial opthalmic nerve (‘Vopts’). Two to three thicker branches on each side, including the ones carrying the latter nerve, continue anteriorly past the nasal cavity, to form the nasobasal canals (‘nbc’). These canals continue anteriorly along the preserved length of the rostrum. They connect with a lateral groove for the maxillary ramus of the trigeminal nerve (‘Vmx’) via a canal, slightly anterior to the nasal cavities. At the same point, a canal leading to the floor of the ethmoidal region branches off (‘paop’). More anteriorly, the nasobasal canals extend gradually to the laterodorsal surface of the braincase, but appear to be contained within the dermal bones, without connecting to the lateral surface of the skull.

##### Remarks

Primitively, in most fossil non-neopterygian actinopterygians, but also in †parasemionotids and †caturids, there are two paired anterior myodomes (dorsal and ventral) notching the posterior wall of the ethmoidal region [44, 58, 63, 66, 98]. In †*Lawrenciella* and †*Kansasiella*, there is a paired anterodorsal myodome, but the anteroventral myodome is median and situated on the interorbital septum [65, 67], as in †*Saurichthys nepalensis*. The aforementioned conditions are likely dependent on the development of the interorbital septum and the orbit. We consider the anterodorsal and anteroventral myodomes, paired or median, to be homologous across taxa. The fenestrations present on the anterior part of the interorbital septum of the Greenland †*Saurichthys* are therefore deemed homologous to the anterodorsal and anteroventral myodomes of most fossil actinopterygians. †*Yelangichthys* exhibits paired anterodorsal and anteroventral myodomes [32], and this may correspond to the primitive condition for the group. Anterior myodomes are absent in acipenseriforms and lepisosteiforms, potentially due to the reduction in orbit size [63].

To date, the internal anatomy of the anterior ethmoidal region in fossil non-neopterygian actinopterygians is virtually unknown, as this region of the braincase is often not mineralized. The nasobasal canals of †*Saurichthys* correspond topologically to the the fenestrae exonarinae anterior in †*Youngolepis* [129] and the nasobasal canals of other Devonian sarcopterygians, such as †*Eusthenopteron* [85] and †*Gogonasus* [130], and to those tentatively reconstructed in †*Mimipiscis* [44]. In these taxa the nasobasal canals begin their course at the anterior margin of the nasal cavity. Actinopterygian nasobasal canals differ from the rostral tubules of sarcopterygians, as the latter issue posterior to the nasal cavities and form a mesially extending, web-like structure (e.g., [95, 131]). Although soft tissue contents remain unknown, the relationship of the nasobasal canals of †*Saurichthys* with branches of the trigeminal nerve, and their communication with the floor of the rostrum, are indicative of at least gustatory functions. They must have also contained blood vessels supplying the growing rostrum. These canals are for the first time confidently reconstructed and described in †*Saurichthys*, or any other fossil actinopterygian.

### Phylogenetic analysis

**Fig. 15:**
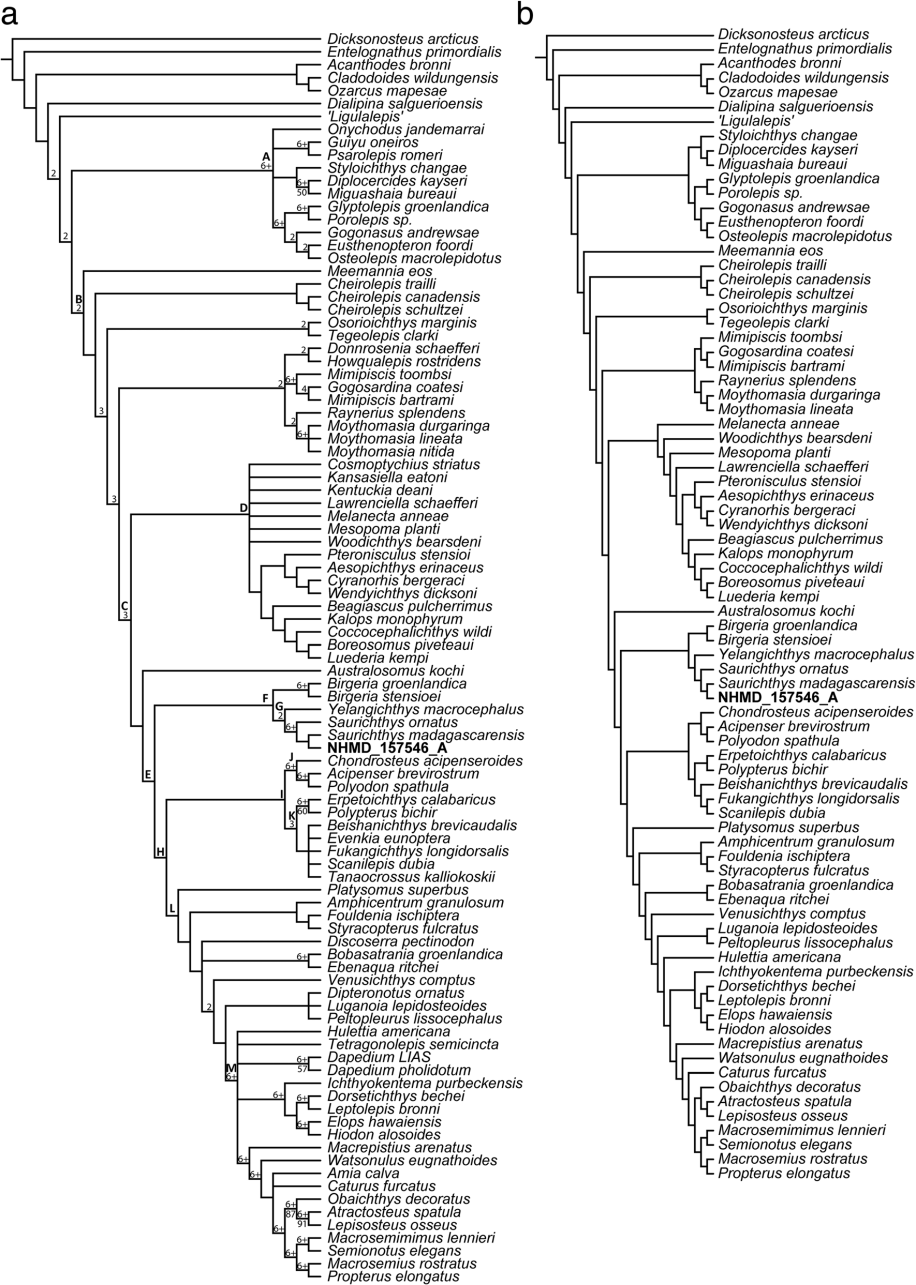
Results of phylogenetic analysis (maximum parsimony). **a** strict consensus of the 2430 MPTs (1421 steps, C.I: 0.217, R.I: 0.645) for 97 taxa and 275 characters of equal weight. Bremer decay indices above 1 are placed above nodes. Bootstrap values above 50% are placed below nodes. Synapomorphies common to all MPTs for selected nodes are as follows: **a** (Sarcopterygii): C.26(1), C.36(0), C.60(1), C.134(1); **b** (Actinopterygii): C.44(1), C.46(0), C.202(1); **c** (post-Devonian Actinopterygii): C.58(0), C.72(0), C.93(2,3), C.124(1), C.133(1), C.139(2), C.141(2), C.144(1), C.146(1), C.186(0), C.191(1), C.194(1), C.201(1), C.221(1), C.243(1); **d** (generalized Carboniferous–Triassic forms): no common synapomorphies; **e** ((†Saurichthyiformes + †*Birgeria*) + crown Actinopterygii): C.68(1), C.102(1), C.148(0), C.155(0), C.159(2), C.177(2), C.184(1),C.244(0); **f** (†Saurichthyiformes + †*Birgeria*): C.53(2), C.66(1), C.213(0), C.215(0), C.240(2); **g** (†Saurichthyiformes): C.20(1), C.31(1); **h** (crown Actinopterygii): C.44(0), C.105(1), C.133(0), C.224(0); **i** (Chondrostei + Cladistia): C.34(1), C.130(0), C.139(0), C.186(1), C.218(0), C.220(0); **j** (Chondrostei): C.69(0), C.92(1), C.104(1), C.107(1), C.160(1), C.177(3), C.185(1), C.212(2), C.221(0); **k** (Cladistia): C.3(0), C.95(1), C.103(1), C.131(1), C.231(1), C.265(0), C.267(1); **l** (total group Neopterygii): C.7(0), C.29(1), C.118(1), C.142(0), C.179(0); **m** (crown Neopterygii): C.74(1), C.75(1), C.115(1), C.121(1), C.182(1). **b** agreement subtree of 2430 MPTs containing 80 taxa

The maximum parsimony analysis produced, after the deletion of suboptimal trees, a total of 2430 most parsimonious trees (MPTs) of 1421 steps (C.I: 0.217, R.I: 0.645). In the strict consensus (Fig. 15a), Actinopterygii is monophyletic, but weakly supported (Bremer decay index [BDI] = 2), with †*Meemania* and †cheirolepidids being successive sister groups to the remaining members of the group. †*Osorioichthys* and †*Tegeolepis* are resolved as a deeply diverging clade on the actinopterygian stem, followed by a clade formed by the remaining Devonian taxa (BDI = 2). All post-Devonian actinopterygians form a clade (BDI = 3), supported by 15 synapomorphies. Post-Devonian taxa are divided in two, albeit weakly supported clades. The first clade contains all Paleozoic-early Mesozoic anatomically generalized forms, whose monophyly is supported by characters that cannot be assessed in most taxa. †*Australosomus* is resolved as the sister taxon to the clade that contains †saurichthyiforms + †*Birgeria* and crown actinopterygians. The immediate sister group relationship between †saurichthyiforms + †*Birgeria* and the actinopterygian crown is supported by eight synapomorphies, none of which is unambiguous: i) absence of complete enclosure of spiracle by canal-bearing bones (C.68); ii) palatoquadrate forming separate ossifications (C.102); iii) absence of vestibular fontanelles (C.148); iv) dorsal aorta open in a groove (C.155); v) lateral dorsal aortae bifurcating below parasphenoid (C.159); vi) posterior stem of parasphenoid extending to basioccipital (C.177); vii) presence of an aortic notch in parasphenoid (C.184); viii) absence of a triradiate scapulocoracoid (C.244).

Our analyses recovered †saurichthyiforms (inclusive of †*Yelangichthys*) as a clade (BDI = 2), with †*Yelangichthys* being the sistergroup to †saurichthyids, on the basis of: i) both nostrils accommodated within single ossification (C.21); ii) frontals broad posteriorly, but tapering anteriorly (C.31). Amongst †saurichthyids, †*Saurichthys madagascariensis* and NHMD_157546_A form a clade to the exclusion of †*Saurichthys ornatus*. †Saurichthyiforms cluster with †*Birgeria* (BDI = 1), sharing the following characters: i) presence of more than two infraorbitals (C.53); ii) head of dermohyal projecting above opercle (C.66); iii) absence of peg-and-socket articulation on scales (C.213); iv) absence of an anterodorsal process on scales (C.215); v) absence of an anocleithrum (C.240). The placement of the clade containing †saurichthyiforms and †birgeriids as sistergroup to the actinopterygian crown has very low nodal support (BDI = 1).

Within the actinopterygian crowngroup, cladistians (†scanilepiforms + polypterids; see [12]) are resolved as sister to chondrosteans. This unusual, and poorly supported (BDI = 1) topology is based on six synapomorphies: i) presence of a posterior junction between supraorbital and infraorbital canals (C.34); ii) presence of a broad interorbital septum (C.130); iii) absence of a posterior myodome (C.139); iv) anterolaterally diverging olfactory lobes (C.186); v) absence of fringing fulcra (C.218); vi) hyomandibula imperforate (C.220). Chondrostei receive high nodal support (BDI ≥ 6), but support for Cladistia is moderate (BDI = 3). A number of Paleozoic–early Mesozoic taxa, most of which are deep-bodied, form branches at the base of the neopterygian stem. †*Platysomus* is the deepest-diverging taxon on the neopterygian stem (BDI = 1), and is united with the remaining neopterygian total group by: i) premaxilla not contributing to the orbit (C.7); ii) quadrate parietals (C.29); iii) vertical preopercle (C.118); iv) presence of a basipterygoid process (C.142); v) absence of a buccohypophyseal canal (C.179). †*Peltopleurus*, †*Luganoia*, and †*Dipteronotus* form a clade at the neopterygian stem. The neopterygian crown is well supported (BDI ≥ 6), on the basis of: i) maxillary kinesis (C.74); ii) peg-like process on maxilla (C.75); iii) subopercle forming anterodorsal process (C.115); iv) interopercle present (C.121); vi) internal carotids piercing parasphenoid (C.182). The interrelationships of crown neopterygians, however, are not clear due to the uncertain placement of †*Tetragonolepis*, †*Hulettia* and †dapediids relative to teleosts or holosteans.

## Discussion

### Phylogenetic position of †Saurichthyiformes and implications of new anatomical data

The new anatomical features of the cranial endoskeleton of †*Saurichthys* described herein allow us to reconsider characters previously used to assess the relationships of the genus with other actinopterygians. †Saurichthyiforms exhibit a combination of primitive (e.g., contact of infraorbital and supraorbital canals between external nares; co-ossified neurocranium; craniospinal processes; dermohyal fused on hyomandibula; absence of an opercular process on hyomandibula) and derived (e.g., external elimination of the oticooccipital fissure; absence of an endoskeletal aortic canal; absence of vestibular fontanelles; absence of endoskeletal basipterygoid process; posterior elongation of parasphenoid; separate ossifications of palatoquadrate) characters, which collectively indicate a close phylogenetic proximity to the base of the actinopterygian crown.

†*Yelangichthys* is confirmed as a †saurichthyiform [32]. The recently proposed immediate sister-group relationship between †*Saurichthys* and crown actinopterygians [12] is favored in our analysis, although nodal support is very low. In contrast to the previous analysis [12], †saurichthyiforms and †*Birgeria* form a clade. Although the two taxa have been previously recovered in a clade [18], most phylogenies resolved †*Birgeria* as the most stemward member of Chondrostei [16, 19, 20, 32]. We note that the †*Birgeria* + †saurichthyiform relationship presented here is weakly supported, and could be challenged in the future. Amongst the key factors uniting the latter two groups are the absence of a peg-and-socket articulation and the absence of an anterodorsal process on scales. The endoskeletal anatomy of †*Birgeria* appears to be dissimilar to that of †*Saurichthys*, for example in the presence of an open oticooccipital fissure; the reduction of craniospinal processes; the absence of a dorsal fontanelle; and the apparent differentiation of braincase ossifications [59, 132].

Contrary to many previous analyses, we did not recover a close relationship between †saurichthyids and Chondrostei [16–19, 24, 29, 32], despite their broad similarity in neurocranial and dermal anatomy. Many of the characters uniting the two groups are now found to be widespread around the base of the actinopterygian crown (see above). In addition, other previously evoked similarities between the two groups can now be dismissed. †Saurichthyids were erroneously thought to share with acipenseriforms a rudimentary posttemporal fossa [16], but this feature is absent in both groups (as well as stem actinopterygians and polypterids). Our reinterpretation of the basicranial circulation pattern in †*Saurichthys* is also of particular importance. The common carotids are now shown to penetrate the parasphenoid posteroventrally to the ascending processes in †saurichthyids, and conceivably in †*Yelangichthys*, forming a complete circulus cephalicus and parabasal canals. These features were previously believed to be absent, as for acipenseriforms [16, 19, 29, 32]. We note the presence of a lateral cranial canal, suborbitals, fused dermohyals, and lateral gulars in Early Triassic †saurichthyids, all of which were previously coded as absent, favoring a chondrostean topology [16, 18, 19]. The presumed increased height and width of the ascending processes of the parasphenoid, and their broad overlap of the lateral commissure in †saurichthyids, †*Birgeria* and chondrosteans, were combined into a single character state in past analyses, setting them apart from the condition seen in taxa like *Amia*, or †*Pteronisculus* [16, 17, 19]. We found evidence to support a single character to capture these complex anatomies lacking, given the fact that acipenseriforms possess thin ascending processes [74, 84], reaching the spiracular opening like in many other stem and crown actinopterygians [7, 58, 59]. The dorsoventral extent of the ascending processes could be of phylogenetic importance, but it remains difficult to assess in laterally-flattened fossils.

Despite the poorly supported tree topology, we observed some similarities between †saurichthyids and acipenseriforms, which appear as homoplasies in this study. Amongst these is the apparent functional resemblance of the tectosynotic fossa, which in both clades seems to perform the same function (attachment of hyoopercular and branchial musculature). We also noted the presence of intramural diverticula opening to the fossa bridgei in both taxa, though these features must have also been widespread in generalized actinopterygians [65]. These features could influence future phylogenies, when more neurocranial data from fossils become available. The reduction of the opercular bone and the corresponding process on the hyomandibula appear as homoplasies under our phylogenetic scheme. Other features, such as the absence of peg-and-socket articulation and the lack of an anterodorsal process on scales, appear as parallelisms between chondrosteans and †saurichthyids + †*Birgeria*, but these characters are difficult to assess in fossils.

### Shape of the actinopterygian tree and directions for future research

The interrelationships of Devonian actinopterygians remain unchanged from the latest analysis involving a previous version of this matrix [12]. †*Meemania* and †*Cheirolepis* are successively crownward members of the actinopterygian stem, an arrangement also well established by other works (e.g., [3, 68, 69, 133]). The clustering of post-Devonian actinopterygians, albeit weakly supported, is congruent with Giles et al. [12] and might reflect a bottleneck in actinopterygian evolution related to the Hangenberg Event [134, 135], or simply a need to re-examine the anatomy of these taxa using modern investigative techniques. Our strict consensus exhibits a Carboniferous-Triassic generalized actinopterygian clade, though nodal support is very low.

The divergence age of crown actinopterygians appears congruent with the hypothesis of Giles et al. [12], as it only contains Carboniferous or younger taxa. However, in our phylogenetic hypothesis, the interrelationships of crown actinopterygians are rearranged. We recovered cladistians and chondrosteans as a clade, in spite of morphological [12, 16–20, 41, 44, 133], and strong molecular [4, 5, 136] evidence supporting cladistians as sister group to Actinopteri (the historical group containing chondrosteans and neopterygians to the exclusion of cladistians [41]). We note that our topology is weakly supported. Moreover, cladistians and chondrosteans constitute particularly long phylogenetic branches, lacking early representatives from the Paleozoic, or the Triassic in the case of the latter. Neurocranial data from early chondrosteans are almost absent [11, 118], and there is a considerable gap of knowledge related to the basicranial circulation, endocast and posterior neurocranial anatomy of early cladistians [12].

In contrast to Giles et al. [12], †*Platysomus* branches from the neopterygian, rather than the chondrostean stem, with other Paleozoic-Mesozoic deep-bodied taxa also branching deep on the neopterygian stem. †*Amphicentrum* forms a clade with the †styracopterids (see †eurynotiforms [120]), but this clade was previously found to branch outside the actinopterygian crown [12]. A close relationship between †eurynotiforms and other deep-bodied taxa is also implied by previous phylogenies [17, 20, 107, 112], but see [120]. However, these forms show conspicuous phylogenetic fluidity, alternating in positions amongst the actinopterygian, the chondrostean, and the neopterygian stem [12, 17, 20, 107, 112, 133, 137], and their endoskeletal anatomy requires investigation. Previous anatomical information for these forms is largely limited to homoplasic features of their external dermal skeleton [61, 119, 138, 139], with the exception of †*Amphicentrum* [64]. †*Peltopleurus*, †*Dipteronotus* and †*Luganoia* are consistently affiliated with the neopterygian stem [12, 17, 20, 137]. This longstanding hypothesis is also reflected in our trees. Endoskeletal data from stem neopterygians is limited [39, 63], but given their likely systematic position, such knowledge seems pivotal for understanding the early evolution of neopterygian anatomy. The monophyly of the neopterygian crown and its immediate sister groups [12] remains unchallenged in our phylogenetic scheme, despite a loss of resolution within the crown.

### Cranial fossae diagnosis, function and evolution in Actinopterygii

**Fig. 16:**
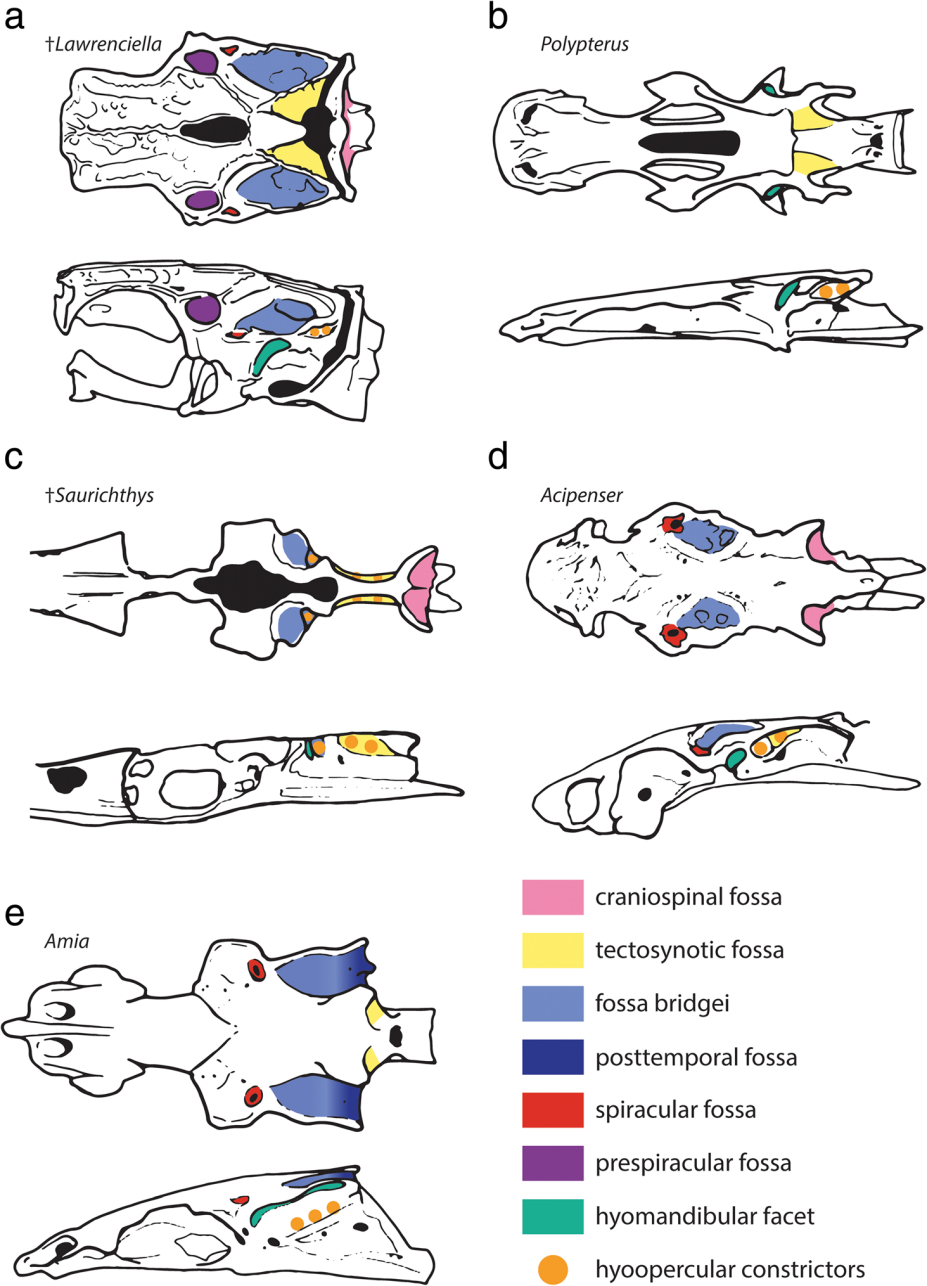
Cranial fossae of the occipital and otic regions and hyoopercular muscle attachment fields of selected actinopterygians. **a** †*Lawrenciellla schaefferi* (redrawn from [67]); **b** *Polypterus* (redrawn from [79]); **c** †*Saurichthys* (based on NHMD_157546_A); **d** *Acipenser brevirostrum* (based on FMNH 113538); **e** *Amia calva* (redrawn from [70]). **Craniospinal fossa**: fossa on the posterior surface of the craniospinal processes, for the accommodation of epaxial muscle segments. Absent when craniospinal processes are absent; **Tectosynotic fossa:** paired fossae bounded laterally by the arch of the posterior semicircular canal. Non-homologous across taxa. **Fossa bridgei**: depression roughly constrained by the planes of the three semicircular canals. Absent when the dermatocranium is fused to the dorsal part of the neurocranium (e.g., in *Polypterus*); **Posttemporal fossa**: on the posterior part of the otic region, but lateral to the posterior semicircular canal. In most neopterygians it is confluent with the fossa bridgei, which opens posteriorly to receive epaxial segments; **Spiracular fossa**: depression formed around the dorsal exit of the spiracle; **Prespiracular fossa**: small fossa lateral to the spiracle and the anterior semicircular canal, dorsal to the horizontal semicircular canal and near to the dorsolateral margin of the braincase. Present in some late Paleozoic–early Mesozoic generalized actinopterygians; **Hyoopercular constrictor fields**: Origins of the hyoid and opercular constrictor muscles. Hypothesized in fossil taxa. These attachment fields migrate according to the changes in the orientation of the suspensorium. In †*Saurichthys* the hyoopercular musculature likely originated in the deeper, posterior part of the fossa bridgei and the tectosynotic fossa. In *Amia* there is no fossa developed, but the origin of the muscle corresponds topologically to the subtemporal fossa in †*Australosomus* and many fossil neopterygians. Drawings not to scale

Cranial fossae, located on the occipital and otic regions of actinopterygian braincases, constitute important anatomical landmarks that convey both phylogenetic and functional signals. Despite this, the available terminology is not always established on a solid anatomical basis or homology, leading to the perplexing use of various terms in the literature, which in turn has affected the shape of published trees (see [16–18, 63, 93]). We hereby attempt a re-diagnosis of cranial fossae (Fig. 16), on the basis of their topology, function and their relationships with other cranial landmarks. The scheme presented herein should be treated as a working hypothesis.

#### Craniospinal fossa

This term, coined here, refers to the paired fossae on the posterior surface of the craniospinal processes of most Paleozoic–early Mesozoic actinopterygians. These fossae are confined within the occipital region and likely served for the origin of the first few epaxial muscle segments, as in acipenseriforms [73, 76]. In fossil forms with reduced or absent craniospinal processes, the trunk musculature must have attached to the otic region [59, 63], as in modern polypterids or neopterygians (e.g., [63, 70, 75, 81]). The craniospinal fossae of †*Saurichthys* and acipenseriforms have been previously homologized with the posttemporal fossae in the otic region of neopterygians [9, 16, 17, 32], solely on the basis of their function. However, the formation of the craniospinal fossa in the occipital region, and the posttemporal fossa in the otic region of actinopterygians, dispels any notion of homology between the two (see also [18]).

#### Tectosynotic fossa

The anterior–anterolateral boundary of the tectosynotic fossa is always formed by the otic process of the posterior semicircular canal. However, given the differences in anatomy and orientation of this fossa among sarcopterygians and actinopterygians, as well as among different groups of actinopterygians, the tectosynotic fossa cannot be considered homologous across taxa. It still constitutes an important anatomical landmark, which can convey functional information. A tectosynotic fossa is present in Devonian sarcopterygians such as †*Eusthenopteron*, †*Youngolepis* and †*Diplocercides* (=†*Nesides*), where it likely accommodated epaxial muscles [85, 93, 129]. In chondrosteans, and likely in †*Saurichthys*, the latter fossa accommodates the poorly differentiated dorsal hyoid and opercular retractors (the latter modified to attach to the subopercle), and the underlying branchial levators [73, 76, 77]. Due to the poor development of the otic process in *Acipenser*, the tectosynotic fossa contacts an anterolaterally situated fossa, which hosts part of the hyoid retractor muscle (Additional file 3: Figure S3). The first epaxial muscle segments attach in a shallow topological equivalent of the tectosynotic fossa in polypterids and gars [75, 79, 81]. A very shallow, paired tectosynotic fossa in the otic region of *Amia*, mesial to the posterior semicircular canal, hosts epaxial muscle segments early in ontogeny, which later migrate to the posttemporal fossa [93]. A paired depression occupies a similar position in the posterodorsal part of the otic region in †*Lawrenciella*, †*Kansasiella* and †*Australosomus*, but is oriented towards the posterior dorsal fontanelle [59, 65, 67, 82] and it is, thus, unlikely to have served for muscle attachment.

#### Fossa bridgei

Stensiö [29] coined the term fossa bridgei to describe a paired depression seen on the dorsal part of the braincase of living acipenseriforms, constrained by the planes of the three semicircular canals (Additional file 3: Figure S3). He homologized it with the posteriorly opening depression seen in the posterodorsal otic region of †*Saurichthys*, a view which is accepted here (Figs. 3a, b, 4a, b, 5b). Stem osteichthyans and Devonian actinopterygians like †*Mimipiscis*, †*Moythomasia*, and †*Raynerius*, as well as polypterids, lack a fossa bridgei, as the dermal bones of the skull roof are firmly attached to the dorsal chondrocranium [44, 69, 79, 86]. A fossa bridgei is present in Carboniferous and younger actinopterygians [29, 58, 59, 63, 65, 67, 82], but in crown neopterygians it becomes confluent with the posttemporal fossa [63]. The absence of a fossa bridgei appears to be the primitive condition in Actinopterygii, but this fossa was secondarily lost in polypterids.

#### Posttemporal fossa

This fossa in the otic region of neopterygians (e.g., in †*Dorsetichthys* or †caturids) is primitively delimited by the posterior and horizontal semicircular canals medioventrally, and the dermal skull roof laterally, whereas a bony wall separates it anteriorly from the fossa bridgei [63, 83]. The anterior expansion of the posttemporal fossa in other neopterygians (e.g., *Amia*) [63, 70], and likely also in †*Amphicentrum* [64], eliminated the wall separating it from the fossa bridgei, and the two fossae became confluent. This modification has been linked to the anterior expansion of the epaxial musculature [63].

#### Spiracular fossa

This fossa (=anterior fossa bridgei [58]) lies anterolaterally to the fossa bridgei and contains the dorsal opening of the spiracular canal, and is present only when the latter is developed. The spiracular fossa can be partially confluent with the fossa bridgei. Examples can be seen in †*Lawrenciella*, †*Pteronisculus*, †*Boreosomus*, †*Australosomus*, *Acipenser*, †*Dorsetichthys* and *Amia* [58, 59, 63, 67, 70].

#### Prespiracular fossa

This term corresponds to a depression situated anteromedial to the spiracular fossa, on the postorbital process. It has only been described in †*Lawrenciella* [67, 82], but topologically equivalent depressions are also seen in the reconstructions of †*Boreosomus*, and putatively †*Pteronisculus* [58]. Its function is unknown, but this feature might prove to have phylogenetic value.

#### Hyoopercular retractor muscle origin

The origins of the hyoopercular retractors and the branchial levator muscles of actinopterygians can often be identified in the form of fossae on the neurocranium. The hyoopercular fossae of most actinopterygians differ significantly from those of †*Saurichthys* and the acipenseriforms. In most Paleozoic–early Mesozoic actinopterygians such as †*Mimipiscis*, †*Moythomasia*, †*Raynerius*, †*Kentuckia*, †*Lawrenciella* and †*Australosomus*, the hyoid and opercular retractors, and potentially parts of the branchial levators, originated in a laterally facing, shallow fossa (=fossa parampullaris [93]) on the dorsolateral–lateral part of the otic region, immediately posterodorsally to the hyomandibular facet, lateral to the posterior semicircular canal, and always dorsal to the jugular depression [44, 58, 67, 69, 78, 82]. The same arrangement was likely present in †*Kansasiella*, †*Pteronisculus* and †*Boreosomus*, but the origin of the hyoopercular constrictors is not well-delineated in these taxa [58, 65]. In modern acipenseriforms, a fossa situated lateral to the posterior semicircular canal hosts part of the hyoid constrictor [73, 76, 77] (Additional file 3: Figure S3). The same fossa is putatively also developed in †*Saurichthys*, but in the latter it became confluent with the fossa bridgei. In *Polypterus*, the branchial levators attach to the lateral wall of the opisthotic ridge, though the hyoid and opercular retractors are accommodated in a fossa dorsal to the opisthotic ridge, shared between the opisthotic and the parietal [79]. In actinopterygians with a subvertical suspensorium, like †*Australosomus* and early neopterygians, the hyoid musculature migrated ventrally and was hosted in the subtemporal fossa, which, when developed, lies ventral to posteroventral to the hyomandibular facet [59, 63]. Given our phylogenetic scheme, this condition must have appeared more than once. The subtemporal fossa is not developed in *Amia*, but the hyoid retractor originates from a topologically homologous location on the lateral wall of the otic region [70, 85]. In gars, due to the peculiar morphology of the hyomandibula, the dorsal hyoid and opercular constrictors originate from the dorsal otic region [140].

## Conclusions

The employment of μCT for the detailed study of three-dimensionally preserved crania of †*Saurichthys* sp. (formerly †*Saurichthys* cf. *ornatus*) and †*S. nepalensis*, as well as a re-evalution of the dermal anatomy of other †saurichthyids, uncovered a large number of anatomical features (e.g., cryptic oticooccipital fissure; patterns of basicranial circulation; brain and inner ear endocast; nasobasal canals; fused dermohyal on hyomandibula; reduction of the opercle; identification of the subopercle as the principal component of the opercular series). New information from †saurichthyids, and modern sturgeons, allowed us to test their long-proposed affinities within a broad osteichthyan context. The historical chondrostean topology of †saurichthyiforms is not confirmed by our analyses. Instead, the latter cluster with †*Birgeria*, forming the immediate sister group to crown actinopterygians. However, given the low nodal support near the base of the actinopterygian crown, the recovered tree topology might be challenged by future discoveries. Still, †*Saurichthys*, which may now be considered as one of the very few Permian–Triassic ray-fins whose endoskeletal anatomy is known in sufficient detail, constitutes a valuable model for morphological comparison with not only other penecontemporaneous fossils, but also with recent taxa. The herein described character complex is essential for understanding character transformations that characterize early members of the actinopterygian crowngroup.

The discrepancies between our interpretation, and those of previous workers [23, 29, 39], highlight the need for revision of many classical works of actinopterygian endoskeletal anatomy. The Permian–Triassic actinopterygian diversity, which is currently dominated by classical and largely authoritative interpretations of anatomy [29, 39, 58, 59, 63, 132, 141], is an ideal target for μCT-aided anatomical reinvestigations. Special emphasis should be given to systematically volatile forms like †*Birgeria*. As in the case of †*Saurichthys,* older interpretations are limited by the use of traditional methodologies. Future work and addition of new fossils is expected to help us achieve some better resolution of stem and early crown actinopterygian interrelationships. On a concluding note, we would like to stress the importance of directing future research efforts towards the detailed investigation of the endocranial anatomy (e.g., brain and osseus labyrinth endocast morphology) of extant taxa that remains surprisingly poorly known.

## Additional files

Additional file 1: List of new and modified characters and scoring changes. (PDF 46 kb)

Additional file 2: Phylogenetic matrix and trees. (ZIP 76 kb)

Additional file 3: Additional figures. (PDF 2190 kb)

## Abbreviations

FMNH: Field Museum of Natural History, Chicago, Illinois, USA
MNHN: Muséum national d’Histoire naturelle, Paris, France
NHMD: Natural History Museum of Denmark, University of Copenhagen, Copenhagen, Denmark
PIMUZ: Paleontological Institute and Museum of the University of Zurich, Zurich, Switzerland
UMMP: University of Michigan Museum of Paleontology, Ann Arbor, Michigan, USA
UMMZ: University of Michigan Museum of Zoology, Ann Arbor, Michigan, USA

## Anatomical abbreviations

I: Olfactory nerve
II: Optic nerve
III: Oculomotor nerve
IV: Trochlear nerve
V: Trigeminal nerve
Vmand: Openings for mandibular canal
Vmx: Maxillary ramus of trigeminal nerve
Vopts: Superficial ophthalmic ramus of trigeminal nerve
VI: Abducens nerve
VII: Facial nerve
VIIhm: Hyomandibular trunk of facial nerve
IX: Glossopharyngeal nerve
X: Vagus nerve
aamp: Ampulla of anterior semicircular canal
aci: Common branch of internal carotid artery
acv: Anterior cerebral vein
addf: Mandibular adductor fossa
ang: Angular
aon: Aortic notch
aorb: Orbital artery
apal: Palatine artery
aps: Pseudobranchial artery
art: Articular
asc: Anterior semicircular canal
asp: Ascending process of parasphenoid
au: Autopalatine
auf: Autopalatine fossa
aur: Cerebellar auricle
bb: Bony bar (dorsum sellae)
bbr: Basibranchial
bhc: Buccohypophyseal canal
bhf: Buccohypophyseal opening
cbr1: Ceratobranchial 1
cbr2: Ceratobranchial 2
cc: Crus communis
ccar: Common carotid artery
chy: Ceratohyal
clav: Clavicle
crsf: Craniospinal fossa
crsp: Craniospinal process
d: Dentary
damy: Dorsal anterior myodome
dhy: Dermohyal
dlf: Likely origin of dilatator and/or hyomandibular protractor muscles
dpal: Dermopalatine
dpt: Dermopterotic
dsph: Dermosphenotic
ep: Epiphysis
epi: Epibranchial
epo?: Epiotic-like ossification
eps: Efferent pseudobranchial artery
exsc: Median extrascapular
fb: Fossa bridgei
fm: Foramen magnum
fr: Frontal
hamp: Ampulla of horizontal semicircular canal
hh: Hypohyal
hbr1: Hypobranchial 1
hbr2: Hypobranchial 2
hm: Hyomandibula
hmf: Hyomandibular facet
hpc: Hypophyseal chamber
hsc: Horizontal semicircular canal
hyp: Hypophyseal chamber
ica: Ascending branch of internal carotid artery
id: Intramural diverticulum
io: Infraorbital
ioc: Infraorbital canal
iof: Interorbital fenestra
ios: Interorbital septum
jc: Jugular canal
ju: Jugal
jv: Jugular vein
la: Lachrymal
lacp: Potential origin of levator arcus palatine muscle
latg: Lateral gular
lcc: Lateral cranial canal
mcv: Mid-cerebral vein
mdc: Mandibular canal
mk: Meckel’s cartilage
mpt: Metapterygoid
mx: Maxilla
nao: Narial opening
n?: Putative dorsal ramus of IX or X
nbc: Nasobasal canal
nocc: Spinooccipital nerve
nocc/aocc: Spinooccipital nerve or occipital artery
not: Notochord
nsao: Nasalo-antorbital
occ: Occipital crest
oph: Ophthalmic artery
otp: Otic process
pa: Parietal
pals: Palatal shelf
pamp: Ampulla of posterior semicircular canal
paop: Palatal opening of nasobasal canals
papl: Parietal pit line
part: Prearticular
partr: Prearticular ridge
pbr: Pharyngobranchial
pl?: Putative pit line on dermopterotic
pmy: Posterior myodome
pop: Preopercle
popc: Preopercular canal
porp: Postorbital process
prof?: Putative course of profundus nerve
psc: Posterior semicircular canal
psp: Parasphenoid
pspk: Parasphenoid keel
pt-sc?: Putative posttemporal-supracleithrum
q: Quadrate
qj: Quadratojugal
rbr: Branchiostegal ray
rpmx: Rostropremaxilla
sac: Saccular recess
sang: Surangular
sang?: Putative surangular
scla: Sclera
sclt: Sclerotic ring
so: Suborbital
soc: (trace of) Supraorbital canal
sop: Subopercle
spig: Spiracular groove
supo: Supraorbital
tel: Telencephalon
tmpc: Temporal canal
to: Optic tectum
tsf: Tectosynotic fossa
utr: Utricular recess
vamy: Ventral anterior myodome
vamy+prof?: Ventral anterior myodome and potential course of profundus nerve
vl: Vagal lobe
vo: Vomer.
