# Supplementary material for: Internal cranial anatomy of Early Triassic species of †Saurichthys (Actinopterygii: †Saurichthyiformes): implications for the phylogenetic placement of †saurichthyiforms
Source: BMC Evol Biol. 2018 Nov 1;18:161. doi: 10.1186/s12862-018-1264-4 (PMC6211452; doi:10.1186/s12862-018-1264-4)
Supplement: Supplementary file 1 — List of new and modified characters and scoring changes. (PDF 46 kb) [file 12862_2018_1264_MOESM1_ESM.pdf]

Internal cranial anatomy of Early Triassic species of †*Saurichthys* (Actinopterygii: Saurichthyiformes): implications for the phylogenetic placement of †saurichthyiforms

Thodoris Argyriou, Sam Giles, Matt Friedman, Carlo Romano, Ilja Kogan, and Marcelo R. Sánchez-Villagra

Additional file 1: List of new and modified characters and scoring changes

A. List of new and modified characters added to Giles et al. matrix[1]

B. List of taxon scoring changes

A. List of new and modified characters

C.20: Both nostrils accommodated within single ossification: 0=absent, 1=present (new character). In †saurichthyiforms both external nares are completely encompassed within a single ossification, historically referred to as the nasaloantorbital[2-6].

C.24: Anterior junction between supraorbital and infraorbital canal: 0=absent, 1=between external nares, 2=anterior to external nares (new character).

C.44: Bone carrying otic portion of lateral line canal extends past posterior margin of parietals: 0=absent, 1=present (new character).

C.112: Operculum: 0=absent, 1=present (new character).

C.154: Craniospinal process: 0=absent, 1=present (modified from [7])

C.159: Birfurcation of dorsal aorta into lateral dorsal aortae: 0=open in endoskeletal groove, 1=enclosed in canal, 2=below parasphenoid (state 2 added, see description in [1]). We modified this character to capture the variation in crown actinopterygians taxa that lack an aortic canal, or taxa that have an aortic canal, but whose dorsal aortae or common carotids extend ventral to the parasphenoid. In *Acipenser*, foramina for the efferent branchial arteries are variably present [8, 9], which are here accounted for similarly to the lateral dorsal aortae or common carotids, and are coded as polymorphic 1/2.

C.170: Occipital region ossification pattern: 0=basioccipital and exoccipitals as separate ossifications, 1=comineralized (new character). This character applies only to taxa with separate braincase ossifications. Acipenseriforms are coded as ?, due to the putative homologization of the occipital perichondral ossification with a compound exoccipital-epioccipital [9]. The occipital region is comineralized in polypterids [10].

C.177: [G 118] Parasphenoid: 0=terminates at/anterior to ventral otic fissure, 1=extends across ventral otic fissure, 2=extends to basioccipital, 3=extends past the occipital region, below the first 1-3 vertebrae (state 3 added).

C.181: Parasphenoid pierced by ascending common carotids: 0=absent, 1=present (new character). In †saurichthyids and likely †*Yelangichthys* the common carotids bifurcate after piercing the parasphenoid, at the level of the ascending processes (scored as present). Not to be confused with the condition in some sturgeons where the parasphenoid is pierced by the descending 1st and 2nd efferent branchial arteries, but a circulus cephalicus is absent [8, 9].

C.189: Arrangement of olfactory nerve in orbital region: 0=completely enclosed in endoskeletal olfactory canal, 1=traversing the orbit lateral to the interorbital septum, at times leaving a groove on the latter (new character).

C.204: Lateral cranial canal connects to lateral wall of braincase: 0=absent, 1=present (new character).

C.205: Intramural diverticula opening in fossa bridgei: 0=absent, 1=present (new character).

C.212: Trunk squamation: 0=complete coverage or more than six horizontal rows of scales, 1=reduced coverage (six to two scale rows), 2=trunk mostly naked (modified from [5, 7, 11]). We removed the ordering, but combined the reduced states in previous versions of this character to one. Although state 1 conflates different possible states (which need additional taxa to be accounted for), it serves test the historical hypothesis that trunk squamation reduction is homologous amongst †saurichthyiforms, †acipenseriforms and †*Birgeria* [2, 7].

C.228: Position of symplectic: 0=posterior to the posterior margin of quadrate, 1=medial to the posterior margin of quadrate. Modified from [12].

C. 268: Epineural processes: 0=absent, 1=present (from [12]). Most Paleozoic and early Mesozoic non-neopterygian actinopterygians and many teleosts bear strong posterolaterally expanding epineural processes on lateral surfaces of their neural arches.

B. List of taxon scoring changes

†*Acanthodes bronni*

C.113: ? → -

C.114: ? → -

C.115: ? → -

C.116: ? → -

Acipenser brevirostrum

C.113: 2 → -

C.130: 1 → 0

C.142: 0 → 1

C.159: - → 1/2

C.177: 2 → 3

C.198: ? → 1

C.200: ? → 0

C.201: ? → 1

C.202: ? → 0

C.203: 0 → -

C.210: - → 0

C.211: - → 1

C.213: - → 0

C.215: - → 0

C.216: - → 0

C.218: 0 → 1

C.232: 0 → 0/1

Amia calva

C.159: 0 → 2

C.177: 2 → 3

†*Amphicentrum granulosum*

C.73: 1 → 0

C.156: 1 → ?

Atractosteus spatula

C.159: 0 → 2

C.177: 2 → 3

C.188: 0 → 1

†*Birgeria groenlandica*

C.3: 0 → 1

C.4: 0 → -

C.5: 1 → -

C.7: 0 → -

C.9: 0 → -

C.10: 0 → -

C.11: 1 → -

C.12: 0 → -

C.48: 0 → 1

C.65: 0 → 1

C.66: - → ?

C.67: - → 1

C.71: 0 → 1

C.98: - → ?

C.101: 2 → 0

C.113: 1 → ?

C.159: ? → 2

C.185: 1 → ?

C.210: 1 → 0

C.213: ? → 0

C.215: ? → 0

C.222: 0 → ?

C.246: - → ?

C.245: - → ?

†*Boreosomus piveteaui*

C.190: 1 → ?

C.233: 1 → ?

†*Chondrosteus acipenseroides*

C.29: 1 → 0

C.53: 1 → 0

C.142: 0 → ?

C.143: 0 → ?

†*Cladodoides wildungensis*

C.113: ? → -

C.114: ? → -

C.115: ? → -

C.116: ? → -

C.143: 0 → -

Elops hawaiensis

C.159: 0 → 2

†*Fouldenia ischiptera*

C.44: ? → 1

†*Fukangichthys longidorsalis*

C.224: 0 → 1

†*Hulettia americana*

C.159: 0 → 2

C.177: 1 → 2

†*Ichthyokentema purbeckensis*

C.159: 0 → 2

Lepisosteus osseus

C.159: 0 → 2

C.177: 2 → 3

C.188: 0 → 1

†*Leptolepis bronni*

C.156: 1 → 0

C.159: 0 → 2

†*Luederia kempi*

C.188: ? → 0

†*Luganoia lepidosteoides*

C.53: 0 → 1

C.54: - → 0

†*Melanecta annae*

C.38: 1 → ?

†*Mesopoma planti*

C.188: ? → 0

†*Obaichthys decoratus*

C.159: 0 → 2

†*Ozarcus mapesae*

C.113: ? → -

C.115: ? → -

C.143: 0 → -

C.144: 0 → -

†*Peltopleurus lissocephalus*

C.70: 0 → 1

C.241: 1 → ?

C.266: 0 → 1

Polypterus bichir

C.155: ? → 1

C.156: ? → 0

C.157: ? → 0

C.158: ? → 1

C.159: ? → 1

†*Saurichthys madagascariensis*

C.7: 0 → -

C.14: 1 → ?

C.21: 1 → -

C.22: 1 → -

C.63: 0 → ?

C.65: 1 → ?

C.66: 1 → ?

C.67: 1 → ?

C.102: 0 → ?

C.113: - → 2

C.114: 1 → 0

C.115: - → 0

C.116: - → 0

C.133: 0 → ?

C.138: ? → 1

C.144: 0 → -

C.177: 2 → 3

C.193: 1 → ?

C.242: 0 → ?

C.244: 1 → 0

†*Semionotus elegans*

C.177: 1 → ?

†*Watsonulus eugnathoides*

C.114: 1 → 0

C.159: 0 → 2

References

1. Giles S, Xu G-H, Near TJ, Friedman M: **Early members of 'living fossil' lineage imply later origin of modern ray-finned fishes**. *Nature* 2017, **549**(7671):265-268.
2. Stensiö EA: **Triassic fishes from Spitzbergen**, vol. 2. Stockholm: Almqvist & Wiksells Boktryckeri-A.-B.; 1925.
3. Rieppel O: **Die Triasfauna der Tessiner Kalkalpen XXV: die Gattung *Saurichthys* (Pisces, Actinopterygii) aus der mittleren Trias des Monte San Giorgio, Kanton Tessin**. *Schweizerische Paläontologische Abhandlungen* 1985, **108**:1 - 103.
4. Kogan I, Romano C: **Redescription of *Saurichthys madagascariensis* Piveteau, 1945 (Actinopterygii, Early Triassic), with implications for the early saurichthyid morphotype**. *Journal of Vertebrate Paleontology* 2016, **36**(4):e1151886.

5. Wu F, Chang M-m, Sun Y, Xu G: **A new saurichthyiform (Actinopterygii) with a crushing feeding mechanism from the Middle Triassic of Guizhou (China)**. *PloS one* 2013, **8**(12):e81010.
6. Wu F, Sun Y, Xu G, Hao W, Jiang D: **New saurichthyid actinopterygian fishes from the Anisian (Middle Triassic) of southwestern China**. *Acta Palaeontologica Polonica* 2011, **56**(3):581 - 614.
7. Gardiner B, Schaeffer B, Masserie J: **A review of the lower actinopterygian phylogeny**. *Zoological Journal of the Linnean Society* 2005, **144**:511 - 525.
8. Marinelli W, Strenger A: **Vergleichende Anatomie und Morphologie der Wirbeltiere. IV. Lieferung**. Wien: Franz Deuticke; 1973.
9. Hilton E, Grande L, Bemis W: **Skeletal anatomy of the shortnose sturgeon *Acipenser brevirostrum* Lesueur, 1818, and the systematics of sturgeons (Acipenseriformes, Acipenseridae)**. *Fieldiana: Life and Earth Sciences* 2011, **3**:1 - 168.
10. Allis EP: **The Cranial Anatomy of *Polypterus*, with Special Reference to *Polypterus bichir***. *Journal of Anatomy* 1922, **56**(Pt 3-4):189-294.143.
11. Maxwell EE, Romano C, Wu F, Furrer H: **Two new species of *Saurichthys* (Actinopterygii: Saurichthyidae) from the Middle Triassic of Monte San Giorgio, Switzerland, with implications for character evolution in the genus**. *Zoological Journal of the Linnean Society* 2015, **173**(4):887-912.
12. Arratia G: **Morphology, taxonomy, and phylogeny of Triassic pholidophorid fishes (Actinopterygii, Teleostei)**. *Journal of Vertebrate Paleontology* 2013, **33**(sup1):1-138.
