# Supplementary material for: Internal cranial anatomy of Early Triassic species of †Saurichthys (Actinopterygii: †Saurichthyiformes): implications for the phylogenetic placement of †saurichthyiforms
Source: BMC Evol Biol. 2018 Nov 1;18:161. doi: 10.1186/s12862-018-1264-4 (PMC6211452; doi:10.1186/s12862-018-1264-4)
Supplement: Supplementary file 3 — Additional figures. (PDF 2190 kb) [file 12862_2018_1264_MOESM3_ESM.pdf]

Internal cranial anatomy of Early Triassic species of †*Saurichthys* (Actinopterygii: Saurichthyiformes): implications for the phylogenetic placement of †saurichthyiforms

Thodoris Argyriou, Sam Giles, Matt Friedman, Carlo Romano, Ilja Kogan, and Marcelo R. Sánchez-Villagra

Additional file 3: Additional figures

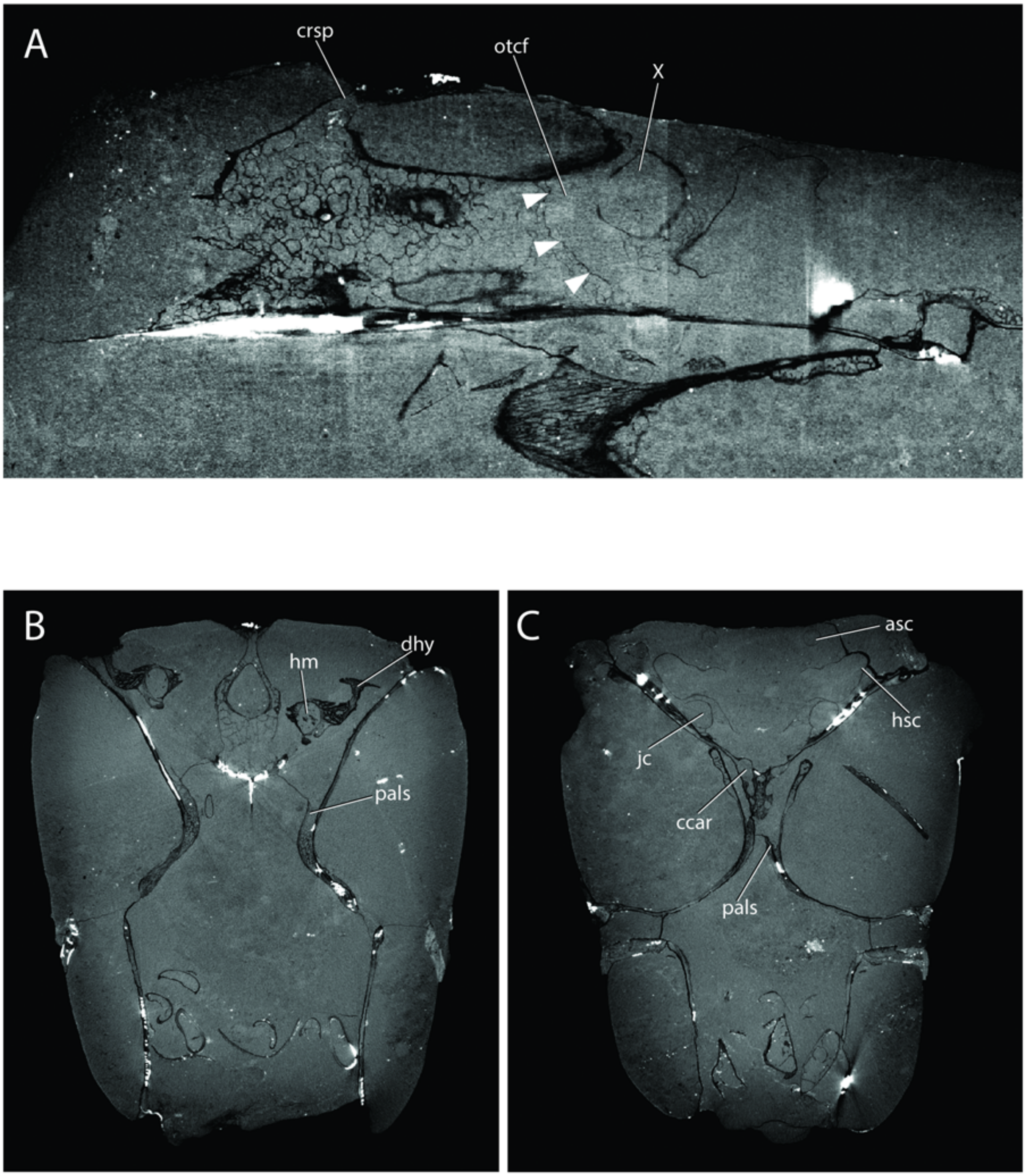

A
crsp
otcf
x
B
hm
dhy
pals
C
asc
hsc
jc
ccar
pals

Additional figure S1. Tomographs of †*Saurichthys* sp. (NHMD_157546_A). **A)** Sagittal tomograph of the occipital region (anterior to the right), showing the presence of the weakly mineralized oticooccipital fissure. The posterior boundary of the fissure (indicated by white arrows) is delineated by the start of the cancellous bone of the occipital region, whereas the anterior boundary is delineated by the perichondral lining of the vagus canal; **B)** Coronal tomograph of the occipital region, showing hyomandibula with fused dermohyal; **C)** Coronal tomograph of the anterior otic region, showing the semicircular canals, the jugular canal and the entrance of the common carotids. Abbreviations: **X,** vagus nerve; **asc,** anterior semicircular canal; **ccar,** entrance of common carotids; **crsp,** craniospinal process; **dhy,** dermohyal; **hm,** hyomandibula; **hsc,** horizontal semicircular canal; **jc,** jugular canal; **otcf,** oticooccipital fissure; **pals,** median palatal shelf.

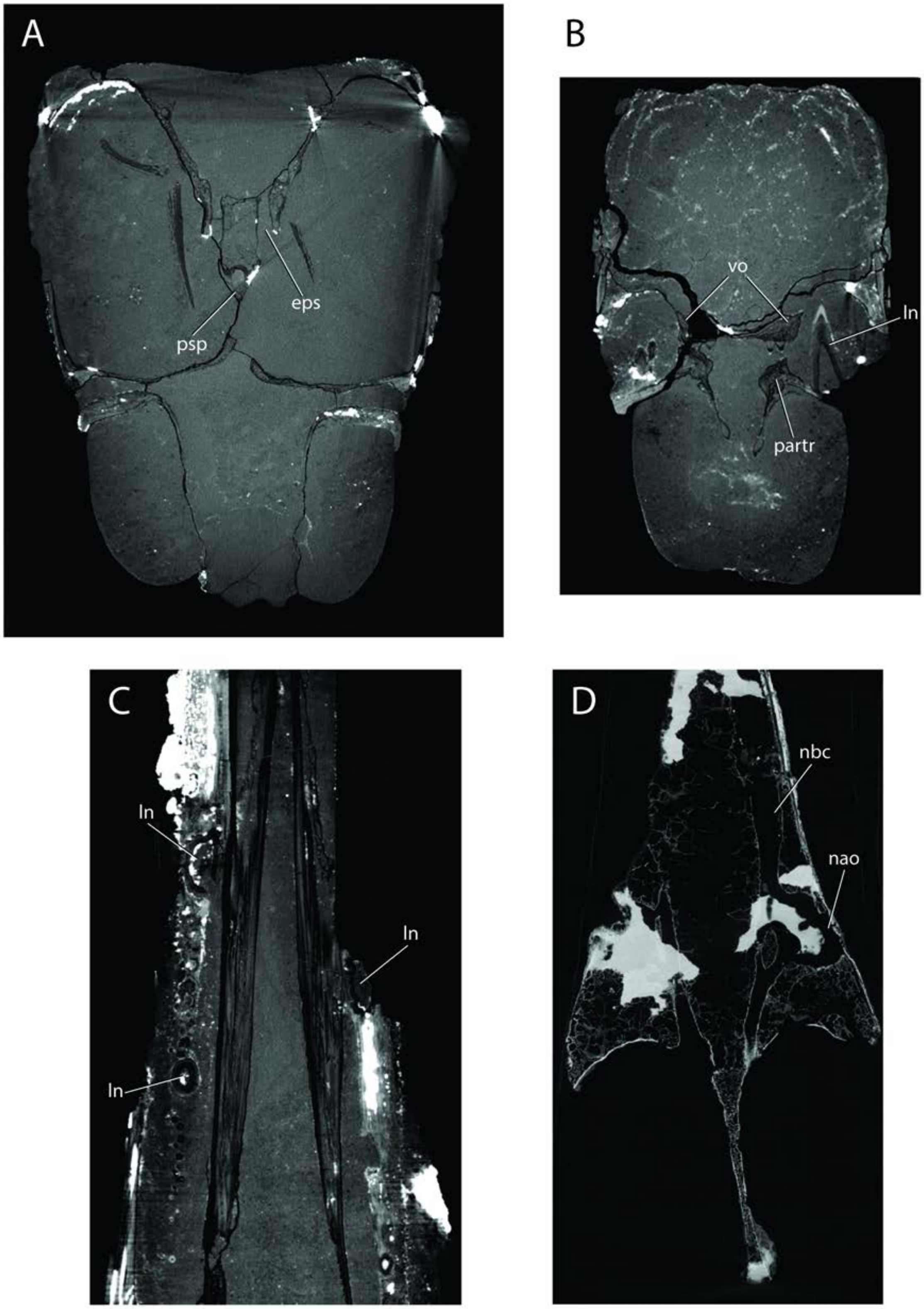
A
psp
eps
B
vo
ln
partr
C
ln
ln
ln
D
nbc
nao

Additional figure S2. Tomographs of †*Saurichthys* sp. (NHMD_157546_A) (**A–C**) and †*Saurichthys nepalensis* (MNHN F 1980-5) (**D**). **A)** Coronal of the sphenoid (s.l.) region, showing the passage of the efferent psedobranchial arteries in relation to the parasphenoid; **B)** Coronal tomograph of ethmoidal region showing the paired vomers, the prearticular and a laniary of the lower jaw; **C)** Axial tomograph of ethmoidal region showing the arrangement of dentition and the presence of plicidentine in the laniaries; **D)** Axial tomograph of ethmoidal region showing the course of the olfactory and nasobasal canals. Abbreviations: **I,** olfactory nerve; **X,** vagus nerve; **asc,** anterior semicircular canal; **ccar,** entrance of common carotids; **eps,** foramen for the passage of the efferent pseudobranchial artery; **ln,** laniary; **nbc,** nasobasal canal; **partr,** prearticular ridge; **vo,** vomer.

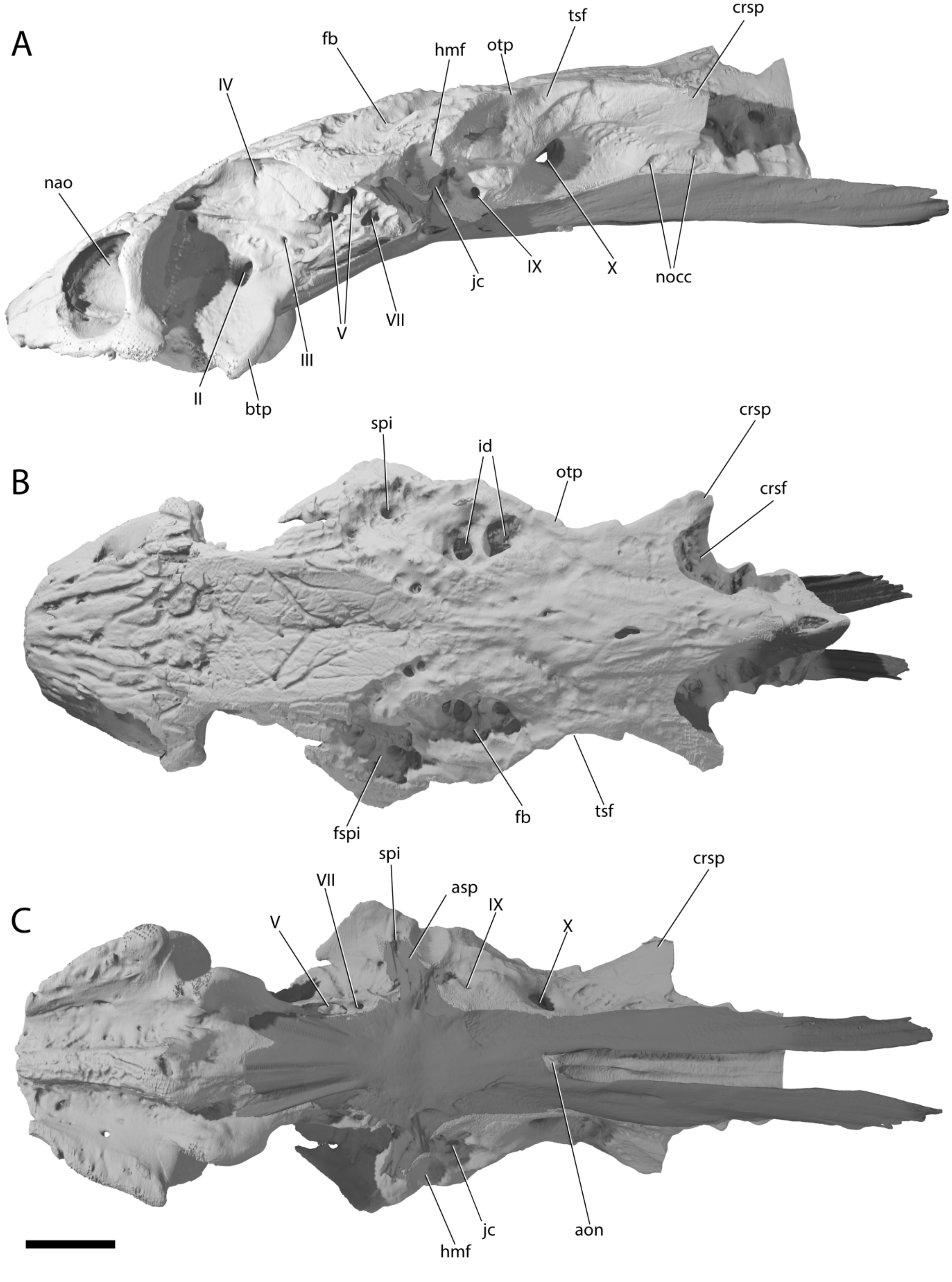
A
fb
hmf
otp
tsf
crsp
IV
nao
jc
IX
X
nocc
V
VII
III
II
btp
B
spi
id
otp
crsp
crsf
fspi
fb
tsf
C
V
VII
spi
asp
IX
X
crsp
hmf
jc
aon

Additional figure S3. Digital rendering of braincase and parasphenoid of *Acipenser brevirostrum* (FMNH 113538) in: **A)** left lateral; **B)** dorsal; **C)** ventral views. Parasphenoid in dark gray shade. Abbreviations: **II,** optic nerve; **III,** oculomotor nerve; **IV,** trochlear nerve; **V,** trigeminal nerve; **VII,** facial nerve; **IX,** glossopharyngeal nerve; **X,** vagus nerve; **aon,** aortic notch; **asp,** ascending process of parasphenoid; **btp,** basitrabecular process; **crsf,** craniospinal fossa; **crsp,** craniospinal process; **fb,** fossa bridgei; **fspi,** spiracular fossa; **hmf,** hyomandibular facet; **id,** intramural diverticulum; **jc,** jugular canal; **nao,** nasal opening; **nocc,** spinooccipital nerve; **otp,** otic process; **spi,** spiracular opening; **tsf,** tectosynotic fossa. Scale bar equals 2cm.

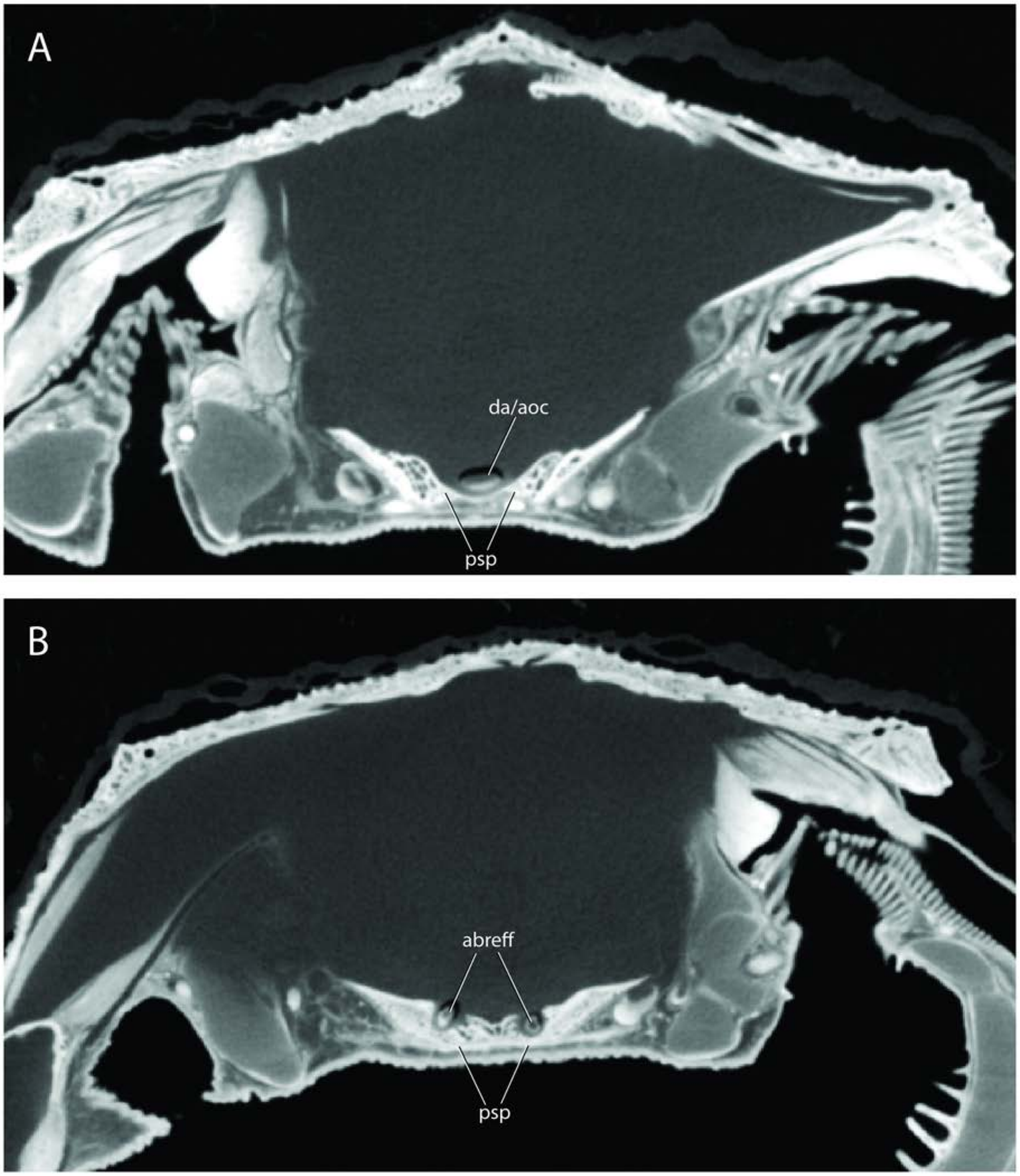
A
da/aoc
psp
B
abreff
psp

Additional figure S4. Coronal tomographs of the occipital region of PTA-stained *Acipenser brevirostrum* (UMMZ 64250), showing aspects of basicranial circulation. **A)** showing dorsal aorta enclosed in endoskeletal aortic canal; **B)** showing bifurcating efferent branchial arteries piercing parasphenoid. Abbreviations: **abreff,** efferent branchial artery; **da/aoc,** dorsal aorta/aortic canal; **psp,** parasphenoid.
